# Polar and subpolar shelf seas dominate enhanced coastal CO_2_ uptake during marine heatwaves

**DOI:** 10.1038/s41467-026-77165-0

**Published:** 2026-09-01

**Authors:** Zhentao Hu, Moritz Mathis, Hongmei Li, Tatiana Ilyina, Yoana G. Voynova, Vlad A. Macovei, Feng Zhou, Qicheng Meng, Corinna Schrum, Wenyan Zhang

**Affiliations:** 1 Institute of Coastal Systems – Analysis and Modeling, Helmholtz-Zentrum Hereon, Geesthacht, Germany; 2 Institute of Oceanography, Center for Earth System Research and Sustainability, University of Hamburg, Hamburg, Germany; 3 Marine Sciences Department, Federal Maritime and Hydrographic Agency, Hamburg, Germany; 4 Department of Climate Variability, Max Planck Institute for Meteorology, Hamburg, Germany; 5 Department of Coastal Productivity, Institute of Carbon Cycles, Helmholtz-Zentrum Hereon, Geesthacht, Germany; 6 State Key Laboratory of Satellite Ocean Environment Dynamics, Second Institute of Oceanography, Ministry of Natural Resources, Hangzhou, China; 7 Observation and Research Station of Yangtze River Delta Marine Ecosystems, Ministry of Natural Resources, Zhoushan, China

**Keywords:** Carbon cycle, Carbon cycle, Physical oceanography, Natural hazards, Environmental impact

## Abstract

Marine heatwaves (MHWs) perturb air–sea CO_2_ fluxes, but their cumulative impact on the global coastal carbon sink remains poorly understood. Using four observation-based CO_2_ flux datasets, with priority given to a global coastal product, we find that MHWs enhance net CO_2_ uptake in global shelf seas by 11.0 ± 1.6% from 1985 to 2020, in contrast to reduced uptake in the open ocean. The enhancement is largely attributable to polar and subpolar shelf seas, where frequent MHWs coincide with sea-ice loss and non-thermal reductions in dissolved inorganic carbon (DIC), outweighing reduced uptake or enhanced outgassing in lower-latitude, warming-dominated regions. Analysis with a global ocean biogeochemical model suggests that the observed DIC reductions are largely linked to enhanced biological carbon fixation. Our findings reveal region-specific MHW impacts on coastal carbon dynamics, highlighting a critical role of high-latitude shelf systems in the global carbon budget under ongoing climate change.

## Abstract (web summary)

This study reveals that marine heatwaves enhance the global coastal carbon sink, driven by polar and subpolar shelf seas through sea ice reduction and biological processes, contrasting with weakened CO_2_ uptake in the open ocean.

## Introduction

The coastal ocean is a highly dynamic and biologically productive interface between land and the open ocean^1–4^, covering ~7% of the global ocean surface. The global ocean currently absorbs approximately 3.1 ± 0.5 Pg C yr^−1^ of atmospheric CO_2_^5^. By comparison, the coastal ocean, defined here as the continental shelf sea, is estimated to absorb 0.18–0.25 Pg C yr^−1^ ^6–9^. Recent assessments tend toward the lower bound of this range as expanded observations of sea surface partial pressure of CO_2_ (*p*CO_2_) and improved statistical interpolation methods have refined global estimates^6,8–12^. This sink is highly heterogeneous in space, with polar and subpolar shelf seas showing substantially higher CO_2_ uptake per unit area than other shelf seas and contributing more than 90% of the total uptake due to their large combined area^8^. At the global scale, temperature-driven CO_2_ solubility generates a broad latitudinal pattern, with high-latitude regions acting as CO_2_ sinks and lower-latitude regions functioning as weak sinks or sources^6,7,13^. However, this pattern is locally modulated by non-thermal processes, including ocean circulation, water mass mixing and biogeochemical dynamics^3,4,6,11,14^. In polar shelves, sea ice further regulates air–sea exchange, modifies upper-ocean properties, and influences biological activities^15–21^. Long-term observations suggest that the coastal CO_2_ uptake has strengthened in recent decades^22^, while modeling experiments propose enhanced biological carbon fixation and increasing atmospheric CO_2_ as the main drivers^3^. Nevertheless, this gradual increase is episodically perturbed by extreme climatic events, such as marine heatwaves (MHWs), droughts, and typhoons^23–26^.

MHWs are defined as prolonged periods of anomalously high sea surface temperature (SST)^27–29^. Their frequency, duration, and intensity have increased in both open-ocean and coastal regions over the past decades^30,31^. These events disrupt marine ecosystems^32–37^ and have substantial socioeconomic impacts^38,39^. In coastal waters, where biological productivity and human activity are concentrated, MHWs exhibit strong spatial heterogeneity^30^ and can exert disproportionately large impacts^24,38,40^. Polar shelf seas are particularly sensitive, not only because MHWs are influenced by long-term ocean warming^41,42^, as is the case globally^28,31^, but also because of their close interaction with sea-ice dynamics in both hemispheres^41–45^. In the Arctic, declining sea ice has been associated with changes in MHW characteristics^41^, whereas in the Antarctic shelf seas, an increase in the occurrence of MHWs promotes sea-ice melt and triggers distinct ecological responses, such as increases in net primary production (NPP)^45^.

Despite growing recognition of MHW effects on air–sea CO_2_ fluxes, most quantitative assessments focus on the open ocean or discrete coastal regions, leaving the global coastal response unresolved. Studies in the open ocean suggest that MHWs suppress CO_2_ uptake by ~8% on average^46^, primarily due to weakened uptake at low to mid latitudes and reduced outgassing in the tropical Pacific^46,47^. However, regional exceptions exist. In the northeast Pacific, for example, certain MHWs enhance uptake through local physical mechanisms^48^. Coastal studies similarly report reduced CO_2_ uptake, especially in areas and seasons that are climatologically sinks^24,26^. These reductions have been linked to weakened winds and elevated surface *p*CO_2_ along the US East Coast^24^ and in the Yellow Sea and East China Sea^26^. In Shark Bay, Western Australia, severe seagrass loss during an MHW transformed one of the world’s strongest blue-carbon sinks into a net carbon source^40^. Such responses raise a critical unresolved question: what is the net effect of MHWs on coastal air–sea CO_2_ exchange across regions with heterogeneous responses? Without resolving this question, the resilience of the coastal carbon sink under ongoing extremes cannot be reliably projected.

In this study, we address this gap through a global, multi-dataset assessment of MHW impacts on coastal CO_2_ fluxes. We use four global ocean CO_2_ flux (*F*CO_2_) reconstruction products (Suppl. Table 1)^9,49–51^, including ULB–SOM–FFN–coastalv2, which was specifically developed for the coastal ocean^9^. This product extends temporal coverage and improves the representation of shelf regions and seasonally ice-covered areas, where observations are sparse. To identify the driving mechanisms, we further employ the ICON-Coast global ocean biogeochemical model^3,52^, which explicitly resolves coastal processes and allows quantification of the relative contributions of biological carbon fixation, physical circulation, and sea-ice dynamics during MHWs. This combined approach provides a framework to evaluate the global impact of MHWs on coastal carbon fluxes, characterize its regional heterogeneity, and assess the resilience of the coastal carbon sink under a warming climate.

## Results

### Coastal CO_2_ exchange anomalies during MHW months

Quantifying the impact of MHWs on air–sea CO_2_ exchange is challenging because daily MHW classifications cannot be directly matched with monthly *F*CO_2_ reconstruction products^24,46,47^. To overcome this limitation, we develop a revised definition of “MHW months” following the framework of Edwing et al.^24^, with SST detrended prior to MHW detection and conventional daily MHW metrics^27^ applied to identify MHW months (see Methods). This approach enables consistent identification of MHW months across two independent SST datasets (Suppl. Fig. 1).

**Fig. 1:**
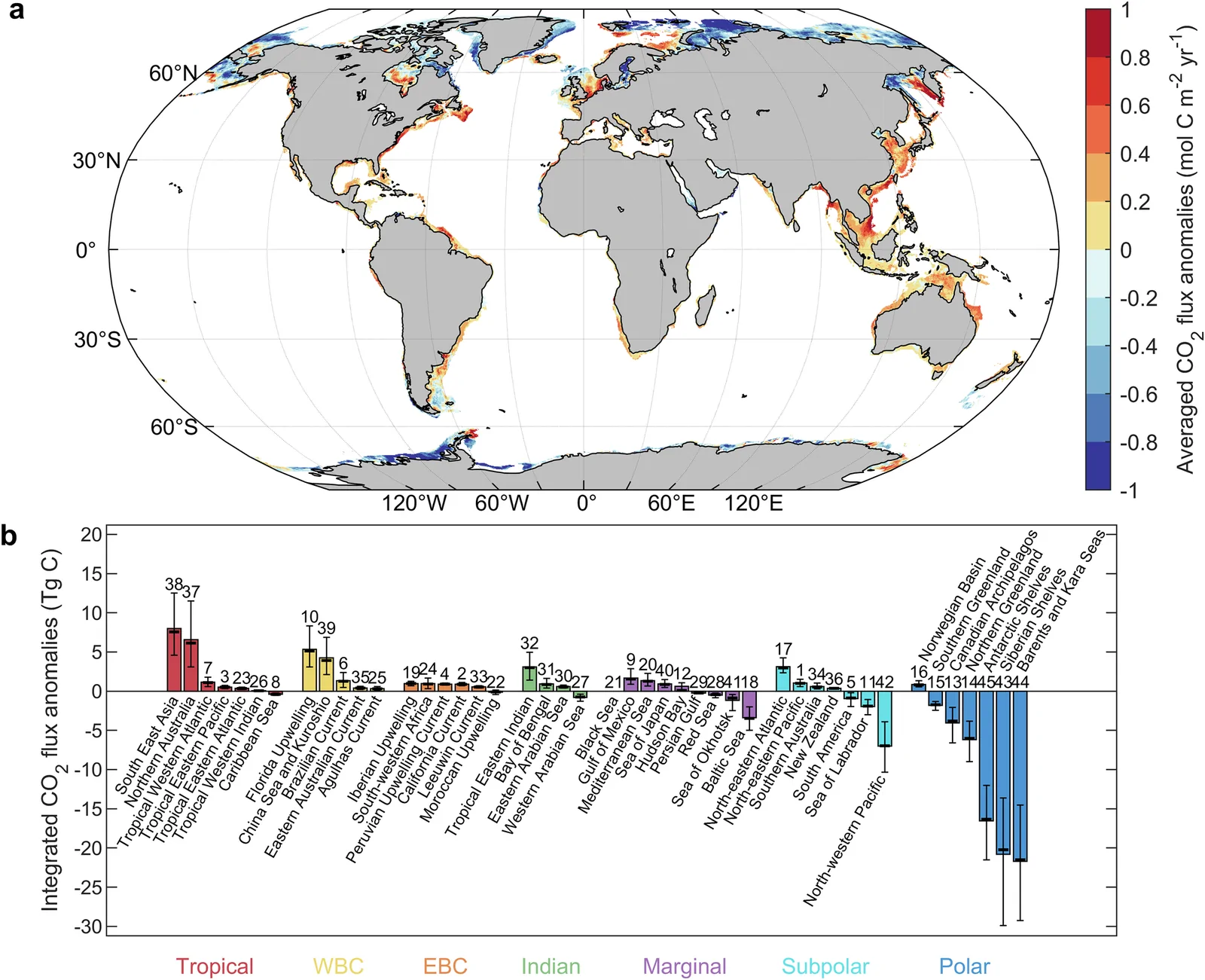
Global coastal CO_2_ flux responses to marine heatwaves (MHWs). **a** Spatial distribution of averaged air–sea CO_2_ flux anomalies (mol C m^−2^ yr^−1^) during MHW months across the global coastal ocean, derived from the observation-based ULB–SOM–FFN–coastalv2 dataset^9^. Positive anomalies indicate enhanced CO_2_ outgassing or reduced uptake, whereas negative anomalies represent enhanced uptake or reduced outgassing. **b** Integrated CO_2_ flux anomalies (Tg C) across 45 coastal segments^56^ from 1985 to 2020 based on the same dataset. Each bar represents the mean anomaly from eleven sensitivity tests (see Methods); error bars denote the full range of estimates. Bold horizontal lines indicate results obtained with a 15-day threshold for defining MHW months, which serves as the benchmark for class ranking. Classes are ordered by total integrated CO_2_ flux anomaly in descending order, with segments within each class further ordered by descending anomaly. WBC and EBC denote western and eastern boundary current regions, respectively. Tropical, Indian, Marginal, Subpolar and Polar denote the corresponding coastal regions. Segment names and their serial numbers are shown.

At the global scale, the climatological spatial sign pattern of coastal *F*CO_2_ largely persists during MHW months, as indicated by the high-resolution (0.25° × 0.25°) ULB–SOM–FFN–coastalv2 product^9^ (Suppl. Fig. 2a,b). High- and temperate-latitude shelf seas remain CO_2_ sinks, while low-latitude regions act as sources. However, the magnitude of *F*CO_2_ anomalies exhibits strong regional variability. Positive anomalies, indicating enhanced CO_2_ outgassing or reduced uptake, prevail in most coastal regions, while negative anomalies, representing enhanced uptake or reduced outgassing, are largely confined to high-latitude, ice-influenced shelf seas (Fig. 1a and Suppl. Fig. 3). These high-latitude shelf seas also display greater variability, reflected in larger standard deviations (Suppl. Fig. 2c). Although anomalies occur broadly, only a subset of regions exhibit statistically significant mean anomalies (Suppl. Fig. 2d), highlighting the strong regional heterogeneity of coastal responses to MHWs. The absence of significant anomalies in many regions (Suppl. Fig. 2d) does not indicate a lack of response but rather the coexistence of competing mechanisms, as the net *F*CO_2_ response to MHWs is strongly influenced by the “tug of war” between thermal and non-thermal drivers^46,47,53^.

Given the strong regional heterogeneity in the frequency of MHW months across the global coastal ocean (Suppl. Fig. 1a, b), particularly the high frequency observed in the Arctic, we quantify the integrated *F*CO_2_ anomaly for the global coastal ocean relative to the trend-preserving climatology (see Methods), while accounting for both the frequency of MHW months and their spatial coverage. Specifically, for each grid cell and month identified as an MHW month, the *F*CO_2_ anomaly is multiplied by the corresponding grid-cell area and converted to carbon units (Tg C). The total integrated anomaly is then obtained by summing across all MHW months and all coastal grid cells, ensuring that both spatial coverage and MHW month occurrence are properly represented. Over the period 1985–2020, the integrated *F*CO_2_ anomaly is –40.02 Tg C, corresponding to an 11.0 ± 1.6% enhancement in global coastal CO_2_ uptake. This pattern is further reinforced by three additional datasets (Suppl. Fig. 4). CMEMS-LSCE-FFNN shows the strongest anomaly (–41.57 Tg C, 12.3 ± 3.0% increase), OceanSODA-ETHZ indicates –18.93 Tg C (4.7 ± 1.1% increase), and SeaFlux records –35.99 Tg C (8.8 ± 0.6% increase for 1990–2019). Collectively, these results provide robust evidence that MHWs enhance integrated coastal CO_2_ uptake. The reported percentage anomalies are calculated by normalizing the integrated anomalies against the corresponding integrated climatological fluxes only for MHW-affected coastal areas and MHW months, following the methodology of Mignot et al.^47^ and Li et al.^46^. When referenced to the cumulative CO_2_ uptake of the global coastal ocean^9^ over 1985–2020 and all months, the reported anomaly of –40.02 Tg C corresponds to ~0.62% of the coastal total uptake, and ~0.05% when referenced to the global ocean^54^. Although this fractional contribution is small, it represents a systematic and measurable response of coastal carbon cycling to MHW events. This response contrasts sharply with the open ocean, where MHWs suppress integrated global CO_2_ uptake by ~8% on average, although the sign and magnitude of the response vary across products^46^. Although rising temperatures are generally known to reduce the solubility of CO_2_ in seawater and thus promote outgassing^55^, our findings indicate that the integrated net response of coastal systems is the opposite, implying stronger compensating feedback in the coastal ocean relative to the open ocean^46,47^.

To better understand regional impacts, we further quantified integrated *F*CO_2_ anomalies (Fig. 1b) across 45 coastal ocean segments (Suppl. Fig. 5) following the definition by Laruelle et al.^56^ (see Methods). In tropical shelf seas and western boundary current (WBC) systems, anomalies are predominantly positive and exhibit the largest increases among all classes, reflecting enhanced outgassing or reduced uptake during MHW months. These patterns are consistent with previous findings from the US East Coast^24^ (region 10 of Margins and CATchment Segmentation^56^; MARCATS 10), the Yellow Sea and East China Sea^26,57^ (MARCATS 39), and Shark Bay^40^ (MARCATS 33). In contrast, most polar and subpolar shelf seas exhibit negative anomalies, suggesting enhanced uptake or reduced outgassing, potentially associated with processes unique to sea-ice regions. On interannual timescales, both spatial integrals and spatial means of *F*CO_2_ anomalies in the polar and subpolar shelf seas exhibit significantly stronger correlations (*P* < 0.05) with global anomalies than those in other regions (Suppl. Fig. 6a, b). These results demonstrate that the global enhancement of coastal CO_2_ uptake during MHW months is dominated by the polar and subpolar regions.

In tropical regions, MHWs amplify coastal CO_2_ outgassing but suppress open ocean outgassing^46,47,58^ (Suppl. Fig. 3), despite both being climatological sources of atmospheric CO_2_. At higher latitudes, polar and subpolar coastal shelf seas exhibit enhanced CO_2_ uptake during MHW months, similar to the open ocean at the same latitudes (Suppl. Fig. 3). In terms of the integrated response, however, the polar and subpolar coastal shelf seas contribute most strongly to the coastal response (Fig. 1b), whereas the open North Pacific contributes most strongly to the open-ocean response^46,47^. Although both are climatological CO_2_ sinks, MHWs enhance CO_2_ uptake in the former (Fig. 1b) but weaken CO_2_ uptake in the latter^46,47^ (Suppl. Fig. 3). These results indicate that coastal and open-ocean systems with similar dominant regional contributions and climatological roles can respond differently to MHWs. Understanding the mechanisms underlying these differences is therefore essential for assessing the contribution of coastal oceans to the global carbon budget during ongoing MHWs.

### Drivers of coastal CO_2_ exchange anomalies during MHW months

To identify the mechanisms driving the regional contrasts in coastal responses, we applied Reynolds decomposition (Eq. 4; see Methods) to isolate the contributions of four key drivers: sea ice cover, wind speed, the ocean–atmosphere CO_2_ gradient (Δ*p*CO_2_), and residual terms (Suppl. Fig. 7a–d). Among these, Δ*p*CO_2_ emerges as the dominant driver of *F*CO_2_ anomalies, as evidenced by the strongest magnitude (Suppl. Fig. 7c) and the highest correlation (*r* = 0.80, *P* < 0.001) with the time series of integrated *F*CO_2_ anomalies (Suppl. Fig. 6c). Δ*p*CO_2_ can be further separated into the thermal and non-thermal contributions of oceanic *p*CO_2_ (*p*CO_2,sea_) and the contribution of atmospheric *p*CO_2_ (*p*CO_2,air_) (Suppl. Fig. 7e–g). The thermal contribution of *p*CO_2,sea_ consistently enhances CO_2_ outgassing by reducing solubility in warmer seawater during MHW months (Suppl. Fig. 7e), a response that is overall statistically significant (Suppl. Fig. 8e). However, the thermal forcing is often offset by non-thermal processes or reductions in sea ice cover, particularly in regions where mean *F*CO_2_ anomalies are not statistically significant (Suppl. Fig. 2d).

**Fig. 2:**
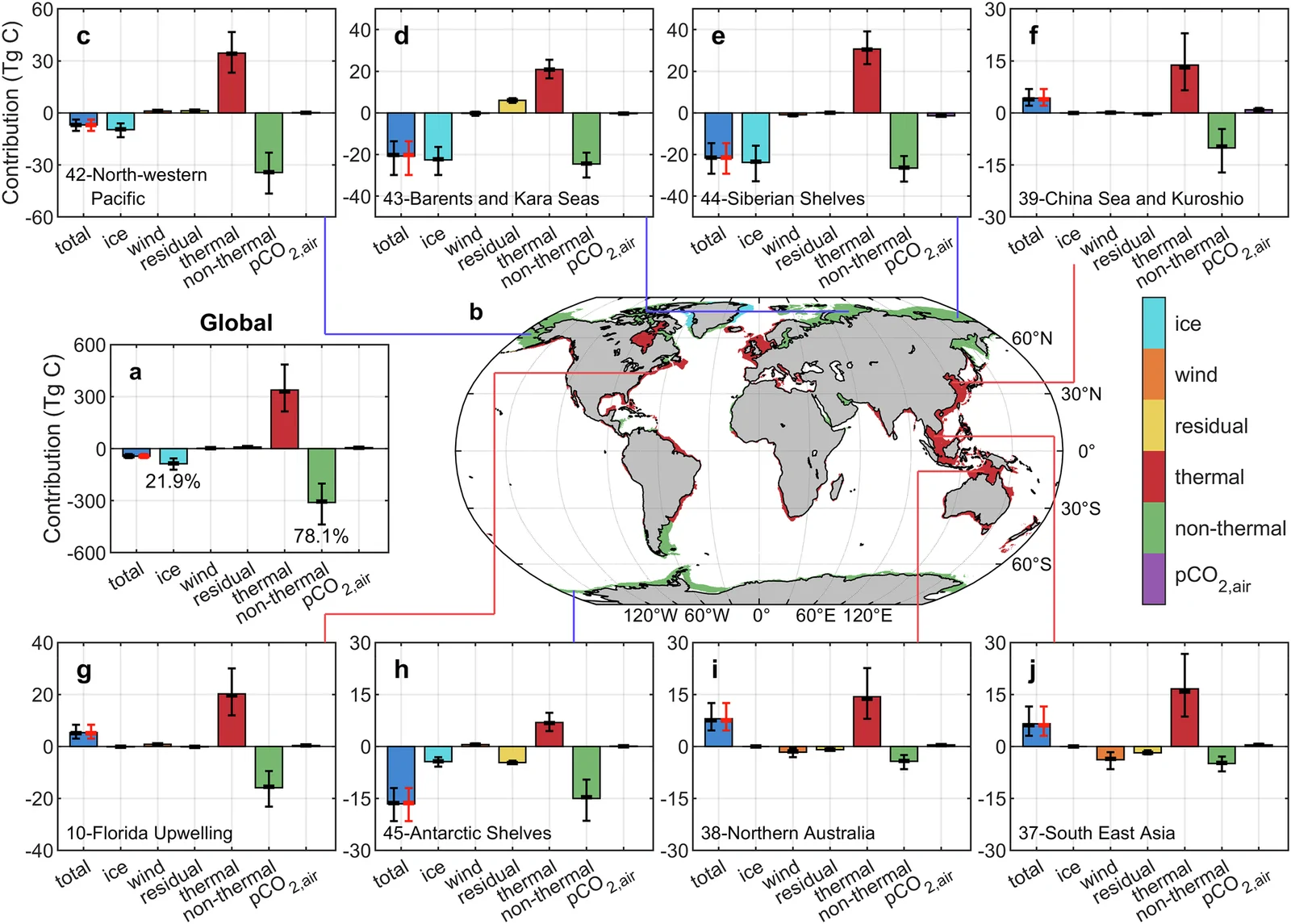
Drivers of air–sea CO_2_ exchange anomalies during marine heatwave (MHW) months from 1985 to 2020. **a** Contributions of individual drivers to global coastal CO_2_ flux (*F*CO_2_) anomalies during MHW months, including sea-ice extent, wind speed, residual terms, the thermal and non-thermal component of the partial pressure of CO_2_ in the seawater (*p*CO_2,sea_), and in the atmosphere (*p*CO_2,air_). **b** Global distribution of the dominant drivers of CO_2_ exchange anomalies. Each grid cell is assigned to the driver that explains the largest portion of the local response. **c–j** Regional breakdown of driver contributions to integrated CO_2_ flux anomalies in typical coastal regions. Bars represent averages from eleven sensitivity tests, and black error bars indicate the full ranges. Bold horizontal lines denote results using a 15-day MHW month threshold. In the “total” column, black error bars represent the sums of all contribution terms from Reynolds decomposition, and red error bars indicate observed anomalies. The close agreement between these terms implies that the decomposition provides a reliable approximation of the integrated *F*CO_2_ anomalies. Each panel is labelled with the corresponding MARCATS region and its identification number. All data shown are derived from the observation-based ULB–SOM–FFN–coastalv2 dataset^9^.

Globally, the thermal contribution of *p*CO_2,sea_ during 1985–2020 is largely compensated by negative contributions from sea-ice reduction (21.9%) and non-thermal anomalies of *p*CO_2,sea_ (78.1%) (Fig. 2a and Suppl. Fig. 6c). Nevertheless, the thermal effect remains the primary driver in most MARCATS regions (Fig. 2b), particularly in tropical and WBC systems such as Northern Australia, Southeast Asia, the Florida Upwelling, and the China Sea and Kuroshio region (Fig. 2f, g, i, j). These patterns align with previous regional observations along the US East Coast^24^ and in the Yellow Sea and East China Sea system^26^. In contrast, high-latitude coastal regions with seasonal sea ice cover are governed by different dynamics. Here, the combined effects of sea ice loss and non-thermal *p*CO_2,sea_ processes exceed the thermal contribution, resulting in enhanced CO_2_ uptake during MHW months. This compensatory response is most evident in the Siberian Shelves, the Barents and Kara Seas, the Antarctic Shelves, and the Northwestern Pacific (Fig. 2c–e, h). Contributions from wind, residual terms, and *p*CO_2,air_ are negligible at the global scale (Fig. 2a and Suppl. Fig. 6c), reinforcing that oceanic processes dominate coastal *F*CO_2_ anomalies during MHW months, as also reported for most of the open ocean^46,47^.

In open oceans, the response of the oceanic carbon sink to anomalous warming is also mainly governed by the balance of thermal and non-thermal drivers^46,47,53^. In the tropical Pacific open ocean, which functions as a climatological CO_2_ source, advection-driven non-thermal anomalies in dissolved inorganic carbon (DIC) dominate over thermal effects^47^, whereas in the North Pacific open ocean, a climatological CO_2_ sink, thermal forcing is the primary driver^46,47^. Open oceans thus exhibit patterns opposite to those of coastal systems with similar climatological roles. This contrast indicates that the hierarchy of physical and biogeochemical controls differs fundamentally between coastal and open-ocean systems. Such heterogeneity emphasizes the need to resolve how sea-ice dynamics and non-thermal mechanisms shape the enhanced CO_2_ uptake observed in high-latitude shelf seas during MHW months.

### Reduction in sea ice cover during MHW months

**Fig. 3:**
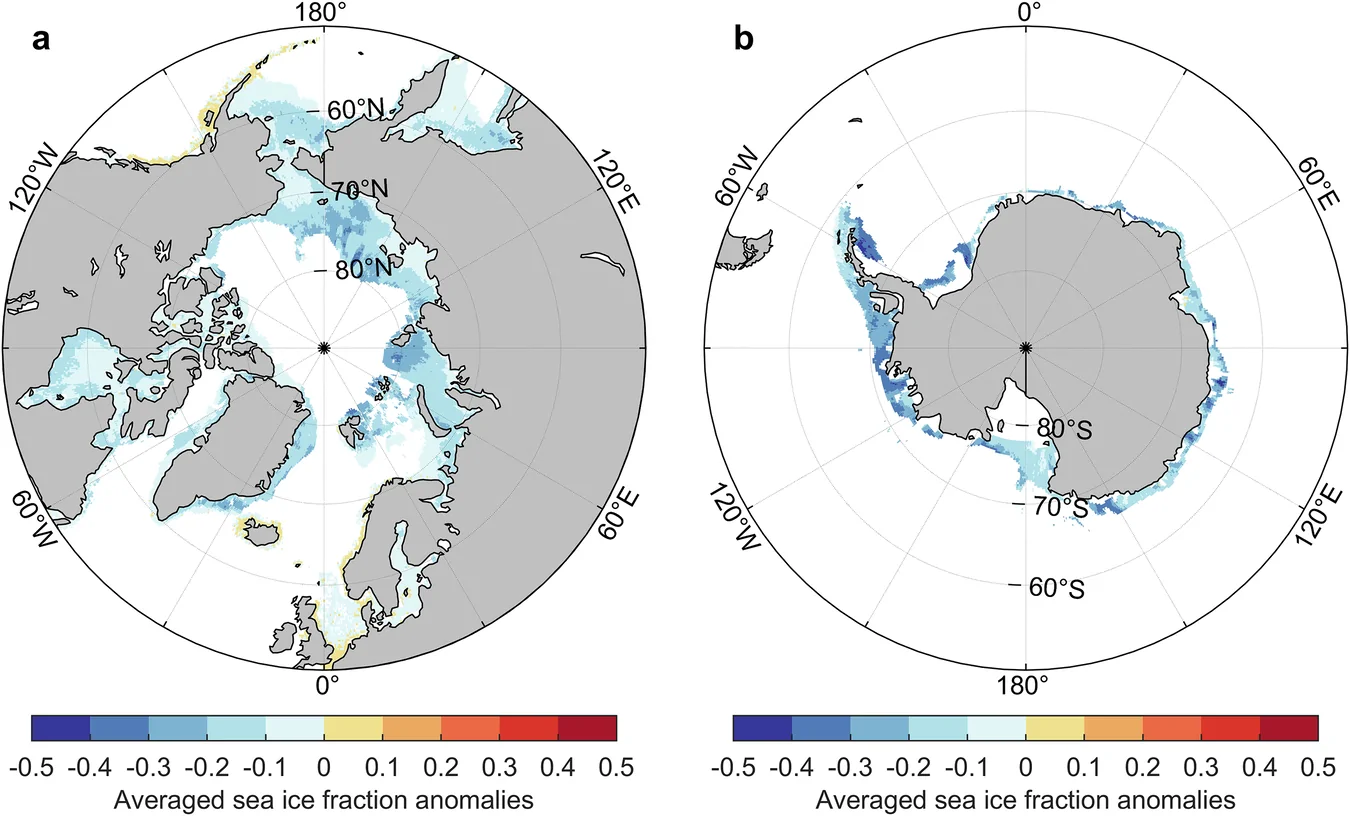
Sea ice reductions during marine heatwave (MHW) months. **a**, **b** Spatial distribution of averaged sea ice fraction anomalies during MHW months (unitless) in the Arctic and subpolar Northern Hemisphere (**a**) and the Antarctic region (**b**). All data shown are derived from the observation-based ULB–SOM–FFN–coastalv2 dataset^9^.

MHWs in polar regions are typically accompanied by substantial sea ice loss^41–45^. Analysis of averaged sea ice fraction anomalies during MHW months reveals widespread reductions in sea ice cover, particularly in the Siberian Shelves, the Barents and Kara Seas, the Antarctic Shelves, and the Northwestern Pacific (Fig. 3). These hotspots of sea ice reduction align with the strongest negative integrated *F*CO_2_ anomalies (Fig. 1b), highlighting the critical role of sea ice loss in amplifying CO_2_ uptake at high latitudes during MHW months. Without the influence of sea ice, the coastal response to MHWs would instead exhibit a consistent decrease in net CO_2_ uptake (Fig. 2a).

The mechanisms linking sea ice reduction to enhanced CO_2_ uptake are multifaceted. Loss of sea ice expands the open-water area exposed to the atmosphere and freshens surface waters, both of which facilitate CO_2_ absorption^20,59^. At the same time, reduced ice cover allows greater light penetration into the upper ocean, stimulating phytoplankton photosynthesis and thereby amplifying the non-thermal *p*CO_2,sea_ anomalies^60,61^. Collectively, these processes reinforce the drawdown of atmospheric CO_2_ during MHW months.

### Biological carbon fixation anomalies during MHW months

**Fig. 4:**
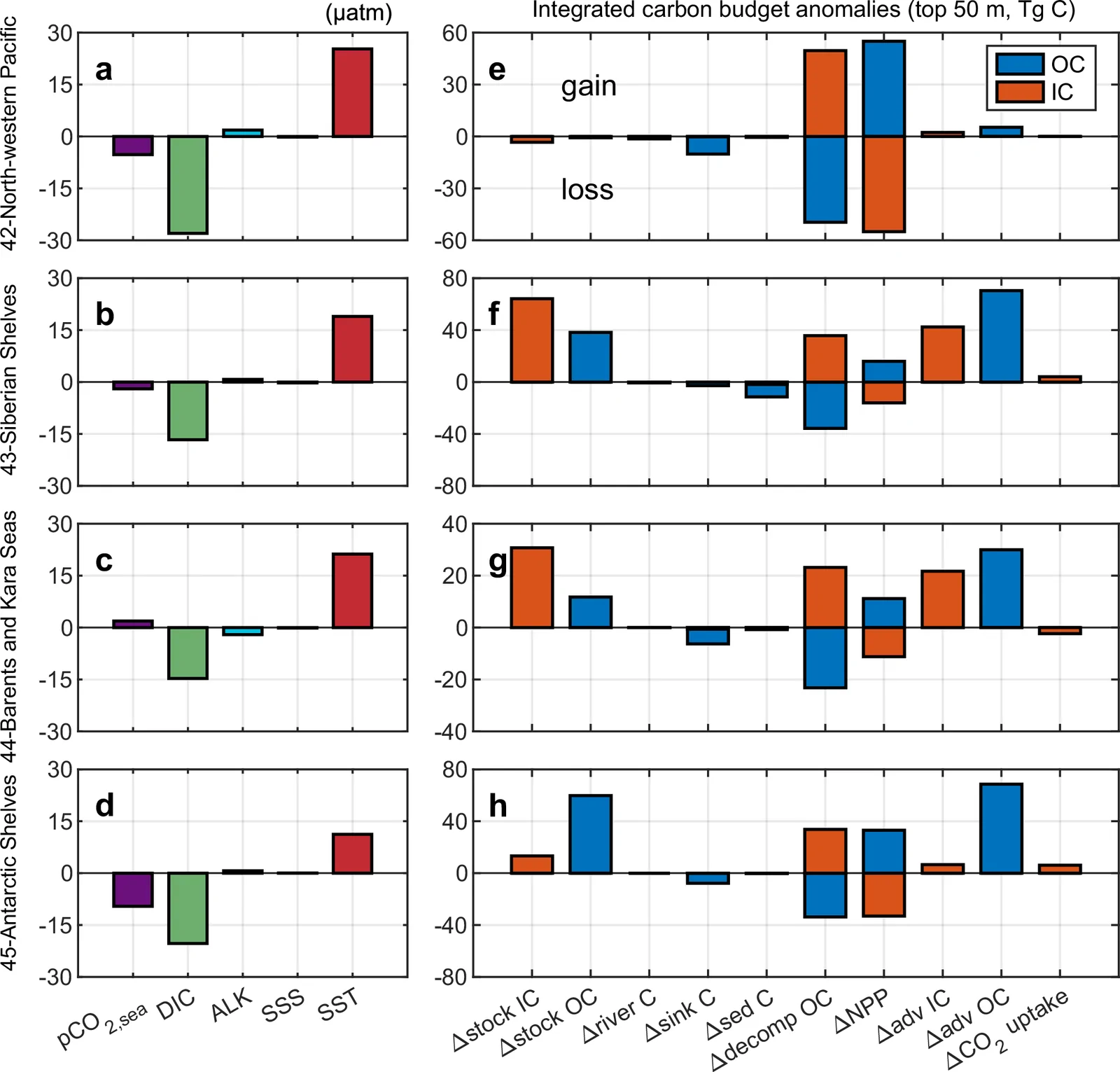
Mechanisms driving non-thermal anomalies in the partial pressure of CO_2_ in the seawater (*p*CO_2,sea_) and the coastal carbon budget during marine heatwave (MHW) months from 1985 to 2020. **a**–**d** Relative contributions of dissolved inorganic carbon (DIC), Alkalinity (ALK), and sea surface salinity (SSS) to non-thermal *p*CO_2,sea_ anomalies in four typical coastal regions: (**a**) the North-western Pacific, (**b**) the Siberian Shelves, (**c**) the Barents and Kara Seas, and (**d**) the Antarctic Shelves, derived from the observation-based CMEMS-LSCE dataset^62^. The sea surface temperature (SST) contributions are also shown in the fifth column. Purple, green, cyan, grey and red bars represent the non-thermal *p*CO_2,sea_ anomaly and the contributions of DIC, ALK, SSS, and SST, respectively. **e**–**h** Integrated anomalies (Tg C) in the upper-ocean (top 50 m) carbon budget of inorganic carbon (IC) and organic carbon (OC) during MHW months for the same regions, based on the ICON-Coast model^52^. Blue and orange bars represent the OC and IC budget terms, respectively.

Non-thermal anomalies of *p*CO_2,sea_ exert a stronger influence on global coastal CO_2_ uptake during MHW months than sea ice loss (Fig. 2a). These anomalies arise from variations in DIC, Alkalinity (ALK), and sea surface salinity (SSS)^14^. Reconstructions from CMEMS-LSCE^62^ reveal that negative DIC anomalies consistently dominate the negative non-thermal *p*CO_2,sea_ anomalies across all coastal regions, with the strongest effects in the North-western Pacific, the Siberian Shelves, the Barents and Kara Seas, and the Antarctic Shelves (Fig. 4a–d). Although the contributions of DIC, ALK, and SSS are estimated using approximate sensitivity coefficients, the signs of their effects remain consistent because the sensitivity coefficients in Eq. (7) vary within a limited range, with *γ*_*DIC*_ ranging approximately from 9 to 15 and *γ*_*ALK*_ from −9 to −15^14^. Even when the most extreme plausible coefficients are applied (${\gamma }_{{DIC}}=9$ and ${\gamma }_{{ALK}}=-14$) for all regions, the DIC contribution still dominates. For example, under these extreme coefficients, the average DIC-induced contribution in the Barents and Kara Seas is approximately −10.17 *μ*atm, whereas the corresponding ALK-induced contribution is about −2.18 *μ*atm, which is substantially smaller. These results confirm that DIC acts as the principal regulator of CO_2_ uptake anomalies. This pattern mirrors findings from the open ocean, where DIC also outweighs ALK and SSS in determining the variability of non-thermal *p*CO_2,sea_^46,53^.

The distribution and variability of DIC are shaped by the combined influence of hydrographic conditions, large-scale ocean circulation, biological production, and air–sea CO_2_ exchanges^47^. To identify the dominant processes, we analyze simulation results from ICON-Coast^52^, a global ocean-biogeochemistry model with an improved representation of coastal carbon dynamics compared to current-generation global Earth system models. ICON-Coast reproduces key observational benchmarks, including integrated coastal CO_2_ uptake, sea-ice extent, and satellite-derived NPP (see Methods), thereby providing confidence in its ability to capture the drivers of coastal biogeochemical variability.

Upper ocean carbon budgets derived from model results suggest that polar and subpolar shelf seas consistently respond to MHW months with marked increases in NPP (Fig. 4e–h). These productivity enhancements are expected to reduce surface DIC and *p*CO_2_, thereby favoring enhanced atmospheric CO_2_ uptake. While simulated surface DIC anomalies broadly exhibit negative anomalies that agree qualitatively with the observed negative DIC anomalies (Suppl. Table 2), changes in the vertically integrated carbon budget terms provide a more comprehensive picture about the net biological response in the upper water column. Anomalies in inorganic carbon (IC) transport and organic matter decomposition outweigh the effects of enhanced biological carbon fixation, resulting in net positive DIC accumulation in several high-latitude regions despite lower DIC concentrations at the surface. Yet, the model does not fully reproduce the observed CO_2_ uptake response, probably due to biases in the individual physical and biogeochemical CO_2_ flux contributions. The increases in NPP are stimulated by warmer surface waters and enhanced light availability following sea-ice loss, which expands open-water area by ~30% during MHW months, as indicated by the model results. Nutrient supply changes are minimal (–0.5%, derived from the model), suggesting that MHW-driven productivity gains are largely sustained by improved light and thermal conditions rather than nutrient inputs. Previous studies reveal that oceanic background nutrient levels regulate phytoplankton responses to MHWs, with weaker blooms in oligotrophic waters and stronger ones in nutrient-rich waters^63–67^. Polar shelf seas are generally characterized by high nutrients^68,69^, providing favorable conditions for the enhanced phytoplankton blooms observed during MHWs^45,70,71^.

Phytoplankton, as a key driver of carbon fixation in marine ecosystems, plays an important role in carbon cycling^72^. Its response to MHWs can propagate through higher trophic levels, influencing zooplankton, fish, seabirds, and marine mammals^73^. The impact of MHWs on phytoplankton shows a clear latitudinal pattern, with chlorophyll generally declining in tropical and mid-latitude regions but increasing at high latitudes^74^. In nutrient-rich polar and subpolar regions with deep mixed layers, MHWs can directly alter upper-ocean stratification^43,45,74,75^, modifying light and nutrient availability and extending the residence time of phytoplankton in the euphotic zone^73^. Recent MHWs in the subpolar North Pacific have been associated with shifts in dominant phytoplankton functional groups^76,77^. In the Antarctic coastal ocean, MHWs can enhance stratification and trigger iron release from melting sea ice, fertilizing waters that are high in nutrients but low in chlorophyll, stimulating phytoplankton growth along the ice edge, and thereby enhancing CO_2_ uptake^45,78^.

While NPP gains are largely offset by enhanced carbon transport and organic carbon (OC) decomposition in the upper water column (Fig. 4e–h), in regions such as the North-western Pacific shelves, NPP increases surpass concurrent increases in OC decomposition, yielding a net positive shift in ecosystem production. This strong biological control contrasts sharply with the mechanism in the open ocean, where negative DIC anomalies primarily arise from horizontal advection^47^. The divergence highlights the disproportionately high productivity of polar and subpolar shelf seas and their critical role in regulating the global carbon cycle during extreme warming events.

## Discussion

In typical tropical and WBC shelf seas, where *F*CO_2_ anomalies are dominated by thermal effects (Fig. 2f, g, i, j), the negative non-thermal *p*CO_2,sea_ anomalies are nevertheless still primarily driven by negative DIC anomalies (Suppl. Fig. 9a–d). However, the biological responses to MHWs in tropical and WBC shelf seas differ fundamentally from those at high latitudes. Overall, advective transport of IC plays the dominant role, whereas the contribution of biological carbon fixation depends strongly on the background nutrient conditions of the ocean. In tropical shelf seas, MHWs suppress net biological carbon fixation by elevating water temperatures and reducing nutrient supply (Suppl. Fig. 9f, g). In contrast, the suppression is weaker in WBC regions, and net productivity is even enhanced in the China Sea and Kuroshio region (Suppl. Fig. 9e, h). Tropical shelf seas are generally nutrient poor^79–81^, providing unfavorable conditions for intense phytoplankton blooms during MHWs^82–84^, whereas WBC regions are generally nutrient-rich^85–87^, supporting favorable conditions for phytoplankton blooms^63^.

Previous studies on the impact of MHWs and anomalously high SST on air–sea CO_2_ exchange have overwhelmingly focused on non-polar oceans, leaving high-latitude regions largely unexplored owing to sparse observations and large uncertainties^46,47,53^. The ULB–SOM–FFN–coastalv2 dataset, developed specifically for coastal regions, addresses this limitation by providing improved predictors and validation, enabling a more robust assessment of coastal carbon fluxes^9^. Building on the framework of Laruelle et al.^6^, which offered one of the first reliable estimates^4^ of coastal CO_2_ uptake at the lower end of reported sink values, this dataset overcomes the common underrepresentation of shelf dynamics in low-resolution reconstructions and captures the contribution of high-latitude shelf seas.

To further strengthen the robustness of our findings, we conduct sensitivity tests using eleven alternative thresholds for defining MHW months. Although the magnitude of flux anomalies varied, the sign, spatial distribution, and dominant contribution from typical coastal oceans remain consistent. These results confirm that the observed enhancement of coastal CO_2_ uptake during MHW months is not a methodological artifact, but rather a robust global feature. Some limitations remain, for example, MHWs spanning two consecutive months but lasting fewer than 15 days per month are excluded, indicating the need for higher temporal-resolution flux reconstructions. Despite remaining uncertainties, the consistency across multiple datasets, definitions, and independent model analyses strengthens the credibility of our conclusions and highlights the necessity of systematically incorporating coastal products into carbon-cycle assessments.

Roobaert et al.^9^ highlighted that reconstructed coastal *p*CO_2_ products may contain biases, particularly in regions with sparse observations or seasonal sea-ice cover. In three of the ten biogeochemical provinces (P1, P5, and P7), the root mean square error (RMSE) exceeds 35 *μ*atm between reconstructed *p*CO_2_ and SOCAT observations^9^. To evaluate the potential influence of such biases on our results, we propagated the upper-bound RMSE into the uncertainty of the air–sea CO_2_ flux anomaly (Suppl. Note 1), thereby deriving a conservative estimate. When aggregated within each of the 45 coastal segments, the resulting uncertainty is relatively small compared with the estimated *F*CO_2_ anomaly (Suppl. Fig. 10). When further aggregated across all coastal regions, the combined uncertainty remains limited (0.91 Tg C), largely because the large number of contributing grid cells reduces the overall uncertainty through statistical aggregation. In addition to reconstruction errors, the coastal flux product itself includes uncertainties associated with Δ*p*CO_2_, gas transfer velocity, wind speed, and sea-ice cover. Roobaert et al.^9^ estimated that the combined effect of these factors results in an uncertainty of approximately 0.01 Pg C yr^−1^ for the integrated coastal air–sea CO_2_ flux. Although MHW months occur more frequently in recent decades with improved observational coverage, where uncertainties are lower than this long-term mean value^9^, we adopt a conservative estimate by assuming temporal and spatial independence of uncertainties and scaling the variance according to the fraction of the coastal domain affected by MHW months (Suppl. Note 1). Under this assumption, the upper-bound uncertainty of 0.01 Pg C yr^−1^ corresponds to an uncertainty of 17.26 Tg C for the integrated CO_2_ flux anomaly during MHW months. The resulting signal-to-noise ratio (SNR) is 2.38, suggesting that the enhanced coastal CO_2_ uptake during MHW months remains relatively robust despite the reconstruction uncertainties.

## Methods

### Segmentation and classification of the coastal ocean

In this study, the coastal ocean is defined using a “narrow” definition, extending to the shelf break while excluding estuaries and inland water bodies^11^. This definition encompasses a total surface area of approximately 28 million km^2^. To capture regional heterogeneity, we adopt the MARCATS framework^56^, which divides global continental shelf seas into 45 regions based on shared climatic and hydrological characteristics. These regions are further grouped into seven major classes^88^: eastern boundary current (EBC), WBC, Indian margins, subpolar margins, polar margins, marginal seas, and tropical margins (Suppl. Fig. 5). This framework accounts for the morphological heterogeneity of the coastal ocean and avoids biases introduced by fixed-distance or depth-based definitions^56^. Within this framework, regional analyses of spatial variations in *F*CO_2_ and their underlying mechanisms were conducted.

### Sea surface temperature datasets and marine heatwave detection

To evaluate the impacts of MHWs on coastal carbon sinks, we employed two global high-resolution SST datasets: the Optimum Interpolation Sea Surface Temperature version 2.1 (OISST v2.1) and the Operational Sea Surface Temperature and Ice Analysis (OSTIA) reprocessed product. OISST v2.1 dataset integrates infrared satellite measurements from the Advanced Very High-Resolution Radiometer (AVHRR) with in situ observations from ships, buoys, and Argo floats^89^. It provides daily SST fields at 0.25° × 0.25° spatial resolution from September 1981 to present^89,90^. Due to its extensive coverage and reliability, OISST v2.1 has been widely used in MHW studies^27,29,46,47^. OSTIA reprocessed (produced by re-running the analysis system on historical observations to generate a consistent long-term climate record) product offers daily, gap-free foundation SST (temperature below the diurnal warm layer and free of diurnal variability) and sea ice concentration data at a higher spatial resolution of 0.05° × 0.05°^91,92^. For this study, OSTIA data were subsequently aggregated onto a 0.25° × 0.25° grid using grid-cell averaging.

MHWs can be defined either relative to the historical climatology or after removing the long-term warming trend to isolate short-term variability, depending on the research objective^93^. In this study, our primary objective is to investigate the anomalous air–sea CO_2_ flux response to MHWs arising from internal climate variability. Therefore, SST data from 1982 to 2020 were detrended prior to MHW detection to remove the long-term warming trend, after which MHWs were identified for the period 1985–2020. MHWs were detected following the widely accepted definition proposed by Hobday et al.^27^, which identifies an MHW as a discrete period of at least five consecutive days with SSTs exceeding the seasonally varying 90th percentile threshold for a given grid cell. To minimize the effects of short-term variability, two events separated by fewer than two days were treated as a single event. The climatological threshold was calculated based on the 1985–2020 reference period, consistent with Hobday et al.^27^, who recommend a baseline climatology of at least 30 years. The MATLAB toolbox *m_mhw*^94^ was used to identify and characterize each event, yielding metrics including onset date, duration, and mean intensity. Notably, applying detrending prior to MHW detection increased the estimated enhancement of global coastal CO_2_ uptake during MHW months from 4.3% to 11.0%. This reflects the exclusion of relatively weak events associated with smaller *F*CO_2_ anomalies and highlights the sensitivity of the estimated response to the choice of MHW detection approach.

In polar regions, the 90th percentile threshold is often very close to the climatological mean, which makes minor SST anomalies near the freezing point sufficient to trigger classification as an MHW. Consequently, many global studies assessing the impacts of MHWs on CO_2_ exchange have excluded the polar regions^46,47^. To address this limitation, previous studies have proposed additional constraints, such as filtering out grid cells with very low SST or SST standard deviation^30,41,44^, applying sea ice concentration (SIC) thresholds^42^, introducing the concept of long-term mean summer temperature (LMST) to avoid detecting weak winter MHWs with low absolute SST^41,45^, or introducing minimum intensity thresholds to avoid detecting weak anomalies near freezing^43^. In this study, we adopted two constraints to improve detection robustness in polar and subpolar regions. First, grid cells with SST standard deviation below 0.2 °C were excluded because they typically exhibit high SIC and low SST variability, yielding less reliable results^41^. Second, MHW events with mean intensity of 0.1 °C or lower in OISST (0.4 °C or lower in OSTIA) were filtered out to eliminate weak anomalies^43^, as OSTIA-derived MHWs are generally more frequent and intense in low-SIC marginal seas^95^.

### Definition of MHW months

To match the monthly resolution of the *p*CO_2,sea_ and *F*CO_2_ data, we defined MHW months by aggregating daily MHW metrics within each month, following the approach of Edwing et al.^24^. For each grid cell, the number of MHW days was calculated for each month during 1985–2020. A month was classified as an MHW month only if it contained at least 15 MHW days, ensuring that the thermal perturbations were strong enough to induce ecological impacts and influence monthly CO_2_ fluxes^24,96^. This criterion ensured that MHW months represented statistically significant thermal anomalies (Suppl. Fig. 8e), while also maintaining a sufficient sample size for robust assessment of carbon cycle responses.

### Observation-based products of *F*CO_2_ and *p*CO_2,sea_

The efficiency of oceanic CO_2_ exchange is determined by *F*CO_2_, which is primarily governed by the *p*CO_2_ gradient between surface ocean and atmosphere. The Surface Ocean CO_2_ Atlas (SOCAT)^97^ has greatly expanded observational coverage, but gaps remain in space and time, particularly in coastal and ice-affected regions. To address these limitations, reconstruction methods using interpolation techniques and machine-learning approaches have been developed to generate continuous spatiotemporal fields of *p*CO_2,sea_ and *F*CO_2_^9,49–51,62^. These approaches extend coverage to regions where observations are sparse^9,11^ and improve the accuracy of carbon flux estimates for both open and coastal ocean^11,50^.

In this study, we employed five observation-based products (Suppl. Table 1), all aimed at providing observation-constrained estimates of surface ocean *p*CO_2_ and air–sea CO_2_ flux, differing in methodology, spatial resolution, and reconstructed variables. ULB–SOM–FFN–coastalv2^9^ combines Self-Organizing Maps (SOM) network with Feed-Forward Network (FFN), specifically targeting the coastal ocean at 0.25° × 0.25° resolution. It extends the work of Laruelle et al.^11^ by including a broader set of predictors and enhanced validation. Its high resolution captures fine-scale variability in coastal air–sea CO_2_ exchange^9,11^, though uncertainties remain elevated in regions with strong spatiotemporal variability and sparse observations.

The other four datasets, mainly developed for global ocean applications, were used to complement and cross-validate the coastal regions. Combining these products into an ensemble framework enhances confidence in the robustness of coastal flux anomalies, particularly in data-sparse high-latitude shelf seas. CMEMS-LSCE-FFNN^49^ employs an ensemble of feed-forward neural networks trained on SOCAT observations and environmental predictors, providing global reconstructions at 0.25° resolution, although uncertainties remain higher in coastal regions and data-sparse regions such as the Southern Ocean and upwelling systems. OceanSODA-ETHZ^50^ employs the Geospatial Random Cluster Ensemble Regression (GRaCER) framework to reconstruct multiple carbonate variables (e.g., *p*CO_2_, DIC, ALK, and pH) at 1° resolution. By incorporating coastal observations and multiple clusters, it improves the representation of coastal variability and extends the coverage into the coastal margin, providing a useful basis for examining coastal carbon flux variability, including under MHW conditions, although uncertainties remain high due to strong spatiotemporal variability and representation errors.

SeaFlux^51^ differs fundamentally from the other products in that it does not generate a new *p*CO_2_ reconstruction but instead provides a harmonized framework for calculating air–sea CO_2_ fluxes from multiple existing *p*CO_2_ products. By standardizing gas-exchange parameterizations, wind products, and flux calculations, SeaFlux enables more consistent comparisons among different flux estimates while reducing methodological discrepancies, although the resulting fluxes remain dependent on the quality and coverage of the input *p*CO_2_ datasets. Finally, CMEMS‑LSCE^62^ provides a 0.25° monthly reconstruction of *p*CO_2_, ALK, DIC, pH, and carbonate saturation states with internally consistent multi-variable estimates suitable for assessing coastal carbon fluxes. It also improves nearshore coverage and representation of the coastal–open ocean continuum, although uncertainties remain high in highly dynamic coastal environments.

### Calculation of air–sea CO_2_ flux

The ULB–SOM–FFN–coastalv2 dataset provides monthly *F*CO_2_ estimates from 1982 to 2020 based on the gas exchange formula:

1 $$F{{{{\rm{CO}}}}}_{2}=\left(k{K}_{0}\right)\times \left(1-{ice}\right)\times \Delta p{{{\rm{C}}}}{{{{\rm{O}}}}}_{2}$$

where *F*CO_2_ (mol C m^−2^ yr^−1^) represents the air–sea CO_2_ flux. Positive values indicate CO_2_ outgassing from the ocean, whereas negative values indicate CO_2_ uptake by the ocean. The gas transfer velocity, *k* (m yr⁻^1^) is calculated as a function of wind speed and the Schmidt number following Wanninkhof et al.^98^. The solubility of CO_2_ in seawater, *K*_*0*_ (mol m^−3^ μatm^−1^), is derived from SST and SSS using the equations of Weiss et al.^55^. The term *ice* is a dimensionless coefficient representing sea ice cover (0 = ice-free; 1 = complete cover). Δ*p*CO_2_ (μatm) is the difference between *p*CO_2,sea_ and *p*CO_2,air_.

### Calculation of trend-preserving climatology and monthly anomalies

Monthly anomalies were calculated to isolate the short-term impacts of MHWs while removing the influence of long-term changes associated with global warming and rising atmospheric CO_2_^99^. For each variable and grid cell, a trend-preserving climatology was calculated over the 1985–2020 period^47,53^ (see Suppl. Fig. 11 for an example). The long-term linear trend was first removed from the time series, and the monthly climatology was then calculated from the detrended data to isolate the mean seasonal cycle. The linear trend was subsequently added back to the seasonal cycle to obtain a trend-preserving climatology. Anomalies during MHW months were then defined as the deviations from the corresponding climatological values. These anomalies specifically capture the instantaneous effects of MHWs relative to the long-term expectation. Consistent with this approach, all climatological fields in this study, denoted with an overbar, retained the long-term trend to ensure methodological consistency.

### Reynolds decomposition of *F*CO_2_ anomalies

The contributions of different drivers to *F*CO_2_ anomalies were quantified using the Reynolds decomposition framework following Couldrey et al.^100^. This framework allows the decomposition of the monthly anomaly of a variable *y*, expressed as a function of three forcing components (*y* = *abc*). This framework is applicable to Eq. (1), where *y* represents *F*CO_2_. The anomaly (*y*′) is calculated as:

2 $$y^{\prime}=y-\bar{y}={abc}-\overline{{abc}}=(a^{\prime}+\bar{a})(b^{\prime}+\bar{b})(c^{\prime}+\bar{c})-{\overline{(a^{\prime}+\bar{a})(b^{\prime}+\bar{b})(c^{\prime}+\bar{c})}}$$

Expanding and rearranging yields the following decomposition:

3 $${y}^{{\prime} }=	 \bar{a}\bar{b}\bar{c}+a^{\prime} \bar{b}\bar{c}+\bar{a}{b}^{{\prime} }\bar{c}+\bar{a}\bar{b}{c}^{{\prime} }+\bar{a}{b}^{{\prime} }{c}^{{\prime} }+{a}^{{\prime} }\bar{b}{c}^{{\prime} }+{a}^{{\prime} }{b}^{{\prime} }\bar{c}+{a}^{{\prime} }{b}^{{\prime} }{c}^{{\prime} }\\ 	 -\overline{\bar{a}\bar{b}\bar{c}+{a}^{{\prime} }\bar{b}\bar{c}+\bar{a}{b}^{{\prime} }\bar{c}+\bar{a}\bar{b}{c}^{{\prime} }+\bar{a}{b}^{{\prime} }{c}^{{\prime} }+{a}^{{\prime} }\bar{b}{c}^{{\prime} }+\,{a}^{{\prime} }{b}^{{\prime} }\bar{c}+\,{a}^{{\prime} }{b}^{{\prime} }{c}^{{\prime} }}\\=	 \underbrace{\bar{a}\bar{b}\bar{c}-\overline{\bar{a}\bar{b}\bar{c}}}_{{zero}-{order\; term}}+\underbrace{{a}^{{\prime} }\bar{b}\bar{c}-\overline{{a}^{{\prime} }\bar{b}\bar{c}}+\bar{a}{b}^{{\prime} }\bar{c}-\overline{\bar{a}{b}^{{\prime} }\bar{c}}+\bar{a}\bar{b}{c}^{{\prime} }-\overline{\bar{a}\bar{b}{c}^{{\prime} }}}_{f{irst}-{order\; terms}}\\ 	+\underbrace{\bar{a}{b}^{{\prime} }{c}^{{\prime} }-\overline{\bar{a}{b}^{{\prime} }{c}^{{\prime} }}+{a}^{{\prime} }\bar{b}{c}^{{\prime} }-\overline{{a}^{{\prime} }\bar{b}{c}^{{\prime} }}+\,{a}^{{\prime} }{b}^{{\prime} }\bar{c}-\overline{{a}^{{\prime} }{b}^{{\prime} }\bar{c}}}_{{second}-{order\; terms}}+\underbrace{a^{\prime} b^{\prime} c^{\prime} -\overline{a^{\prime} b^{\prime} c^{\prime} }}_{{third}-{order\; term}}$$

This decomposition separates *y*′ into four components: a zero-order term, first-order terms (contributions of individual anomalies *a*′, *b*′, *c*′), second-order terms (contributions from interactions between pairs of anomalies), and third-order term (contribution of triple interactions). The anomalies *a*′, *b*′, *c*′ are defined relative to their climatological states ($\bar{a},\bar{b},\bar{c}$), which include the long-term trend and hence differ between years.

In this study, *y* corresponds to monthly *F*CO_2_. The variable *a* represents combined effect of *k* and *K*_*0*_, which is primarily driven by wind speed and largely independent of SST^101,102^. The term *b* and *c* represent sea ice fraction and Δ*pCO*_2_, respectively. Substituting these definitions into the expanded Eq. (3), the anomaly $F{{CO}}_{2}^{\prime}$ can be expressed as:

4 $$F{{CO}}_{2}^{{\prime} }=	 \underbrace{\overline{\left(k{K}_{0}\right)}\overline{\left(1-{ice}\right)}\overline{\Delta {{pCO}}_{2}}-\overline{\overline{(k{K}_{0})}\overline{\left(1-{ice}\right)}\overline{\Delta {{pCO}}_{2}}}}_{{zero}-{order}\,{term}}\\ 	+\underbrace{{\left(k{K}_{0}\right)}^{{\prime} }\overline{\left(1-{ice}\right)}\overline{\Delta {{pCO}}_{2}}-\overline{{\left(k{K}_{0}\right)}^{{\prime} }\overline{\left(1-{ice}\right)}\overline{\Delta {{pCO}}_{2}}}}_{{wind}\,{speed}\,{contribution}}\\ 	+\underbrace{\overline{\left(k{K}_{0}\right)}{\left(1-{ice}\right)}^{{\prime} }\overline{\Delta {{pCO}}_{2}}-\overline{\overline{\left(k{K}_{0}\right)}{\left(1-{ice}\right)}^{{\prime} }\overline{\Delta {{pCO}}_{2}}}}_{{ice}\,{contribution}}\\ 	+\underbrace{\overline{\left(k{K}_{0}\right)}\overline{\left(1-{ice}\right)}\Delta {{pCO}}_{2}^{{\prime} }-\overline{\overline{\left(k{K}_{0}\right)}\overline{\left(1-{ice}\right)}\Delta {{pCO}}_{2}^{{\prime} }}}_{\Delta {pC}{O}_{2}\,{contribution}}\\ 	+\underbrace{\overline{\left(k{K}_{0}\right)}{\left(1-{ice}\right)}^{{\prime} }\Delta {{pCO}}_{2}^{{\prime} }-\overline{\overline{\left(k{K}_{0}\right)}{\left(1-{ice}\right)}^{{\prime} }\Delta {{pCO}}_{2}^{{\prime} }}}_{{second}-{order}\,{term}}\\ 	+\underbrace{{\left(k{K}_{0}\right)}^{{\prime} }\overline{\left(1-{ice}\right)}\Delta {{pCO}}_{2}^{{\prime} }-\overline{{\left(k{K}_{0}\right)}^{{\prime} }\overline{\left(1-{ice}\right)}\Delta {{pCO}}_{2}^{{\prime} }}}_{{second}-{order}\,{term}}\\ 	+\underbrace{{\left(k{K}_{0}\right)}^{{\prime} }{\left(1-{ice}\right)}^{{\prime} }\overline{\Delta {{pCO}}_{2}}-\overline{{\left(k{K}_{0}\right)}^{{\prime} }{\left(1-{ice}\right)}^{{\prime} }\overline{\Delta {{pCO}}_{2}}}}_{{second}-{order}\,{term}}\\ 	+\underbrace{{\left(k{K}_{0}\right)}^{{\prime} }{\left(1-{ice}\right)}^{{\prime} }\Delta {{pCO}}_{2}^{{\prime} }-\overline{{\left(k{K}_{0}\right)}^{{\prime} }{\left(1-{ice}\right)}^{{\prime} }\Delta {{pCO}}_{2}^{{\prime} }}}_{{third}-{order}\,{term}}$$

Here, the zero-order term represents baseline contributions from mean states of the drivers (see Suppl. Fig. 12 for an example). The first-order terms capture the individual contributions of anomalies in wind speed, sea ice cover, and Δ*pCO*_2_. The second- and third-order terms reflect interactions among two or more driver anomalies. In this study, the zero-order term, second order and third-order terms are collectively referred to as residual terms.

### Decomposition of oceanic *p*CO_2,sea_ anomalies

Generally, *p*CO_2,sea_ is the dominant driver of Δ*p*CO_2_ and thus strongly regulates *F*CO_2_^103^. To isolate the influence of MHW-related SST anomalies, we decomposed *p*CO_2,sea_′ into thermal ($p{{CO}}_{2,T}^{{\prime} }$) and non-thermal ($p{{CO}}_{2,\,NT}^{{\prime} }$) components:

5 $$p{{CO}}_{2,\,T}^{{\prime} }=\overline{{{pCO}}_{2}}\times \exp \left(0.0423\left({SST}-\overline{{SST}}\right)\right)-\overline{{{pCO}}_{2}}$$

6 $$p{{CO}}_{2,{NT}}^{{\prime} }=p{{CO}}_{2}^{{\prime} }-p{{CO}}_{2,T}^{{\prime} }$$

where $\overline{{{pCO}}_{2}}$ and $\overline{{SST}}$ denote monthly climatology including linear trends. The coefficient 0.0423 °C^−1^ represents the temperature sensitivity of *p*CO_2,sea_^104,105^. The thermal component reflects the variability directly attributable to SST anomalies during MHW months, while the non-thermal component captures concurrent changes in DIC, ALK, and SSS. Following Müller et al.^53^, we defined $p{{CO}}_{2,T}^{{\prime} }$ relative to the climatological baseline including a linear trend, thereby capturing small, non-seasonal anomalies characteristic of event-driven variability. This definition differs from the classical thermal component, which represents the full seasonal cycle of *p*CO_2,sea_^105,106^. Similarly, our definition of $p{{CO}}_{2,NT}^{{\prime} }$ resembles the residual *pCO*_2_ used in previous studies^53,107,108^, but explicitly excludes the classical seasonal cycle. To quantify the thermal and non-thermal contributions to $F{{CO}}_{2}^{\prime}$, the terms ${pC}{O}_{2,T}^{{\prime} }$ and ${pC}{O}_{2,{NT}}^{{\prime} }$ were substituted into the first-order terms in Eq. (4), where Δ*p*CO_2_ is the difference between *p*CO_2,sea_ and *p*CO_2,air_.

The contributions of DIC, ALK, and SSS to *p*CO_2,sea_′ were further calculated as:

7 $$p{{CO}}_{2,X}^{{\prime} }={\gamma }_{X}\times \Delta X\times \overline{{{pCO}}_{2}}/\bar{X}$$

where *X* represents DIC, ALK, or SSS; $\bar{X}$ is the monthly climatology of *X*; ∆*X* represents the corresponding anomaly; and *γ*_*X*_ is the sensitivity coefficient of *p*CO_2,sea_ to change in *X*. For simplicity, we applied the approximation ${\gamma }_{D{IC}}\approx {-\gamma }_{A{LK}}$, as the surface-ocean values of *γ*_*DIC*_ and *γ*_*ALK*_ are of nearly equal magnitude with opposite sign^109^. In high-latitude coastal regions (MARCATS 12–16 and 41–45), *γ*_*DIC*_ = 13 and *γ*_*ALK*_ = −13, while in other regions, *γ*_*DIC*_ = 11 and *γ*_*ALK*_ = −11^14^. *γ*_*SSS*_ is equal to 1. These coefficients were adopted as approximate values for the present analysis.

### Sensitivity test

To evaluate the sensitivity of our results to the choice of MHW month definition, we repeated analyses by varying the minimum duration threshold of MHW month definition from 11 to 20 days. For each threshold, anomalies in *F*CO_2_, sea ice, wind speed, and *p*CO_2_ were recalculated. Across all cases, the enhancement of coastal CO_2_ uptake during MHW months remained consistent (Suppl. Fig. 4). The magnitude of anomalies decreased slightly with longer thresholds, reflecting a trade-off between the inclusion of more frequent but weaker events at shorter thresholds and fewer but more intense events at longer thresholds. Nevertheless, the spatial patterns of responses and the dominance of shelf seas remained stable across all definitions. The 15-day threshold yielded results close to the average of the eleven sensitivity experiments (Figs. 1b, 2). We therefore adopted this threshold in Figs. 3, 4 as a representative and robust baseline for subsequent analyses.

### Numerical model description

ICON-Coast is a global ocean-biogeochemistry model that seamlessly integrates coastal and open-ocean dynamics within a single framework^52^. It is built on an unstructured triangular mesh that allows telescoping grid refinement, with fine spatial resolution in coastal regions transitions smoothly to coarser resolution in the open ocean^110^. This unified approach naturally captures two-way coupling between coastal shelves and the open ocean at global scale. In the configuration used here, horizontal grid spacing varies from 80 km in open ocean areas to 10 km near coastlines and topographic gradients. Vertically, the model uses 40 z-coordinate levels, with 10 m resolution in the upper 100 m and increasing layer thickness below. The uppermost 16 m include a free surface to represent tidal elevations and sea-ice draft. ICON-Coast incorporates tidal dynamics, including bottom drag, and enhanced parameterizations tailored for coastal environments. The model extends traditional modules of the biogeochemistry component HAMOCC to explicitly include coast-specific processes such as sediment resuspension, temperature-dependent remineralization in the water column and sediment, river inputs including terrestrial organic carbon and nutrients, and variable sinking velocities of aggregated particulate matter^3,52^.

The model was forced with ERA20C (1900–1939) and ERA5 (1940–2020) reanalysis atmospheric data^111,112^, historical time-varying atmospheric *p*CO_2_, as well as atmospheric N deposition^113^ and dust sources^114^. Land-derived inputs to the ocean comprise freshwater runoff^115^, carbon and nutrient fluxes from about 2000 catchments and 850 major rivers^116^, as well as permafrost organic carbon due to Arctic coastal erosion^117^. The model was initialized by the coarser-resolution control run of Mathis et al.^3^ and spun up for 100 years in full high-resolution biogeochemistry mode with looped 1900–1919 forcing. Starting from this state, we ran a historical hindcast over 1900–2020 and used the period 1985–2020 to assess the impact of MHWs on coastal ocean CO_2_ uptake. To account for potential long-term drift, we continued the spin-up simulation by another 120 years as control run.

### Model evaluation

MHW statistics are evaluated by comparing the simulated spatial distribution of MHW-month occurrence and the temporal variability of the integrated annual area affected by MHW months against OISST/OSTIA-based estimates. Overall, the model reproduces the global spatial distribution of MHW-month occurrence reasonably well, although the total frequency of MHW months in high-latitude regions is generally underestimated compared to observations (Suppl. Fig. 13a). This underestimation is likely related to the relatively thick uppermost model layer (~16 m), which dampens near-surface temperature extremes and reduces the occurrence of simulated MHWs relative to satellite-derived SST products representing the ocean skin layer. Despite these regional biases, the model reproduces the interannual variability of the integrated MHW-month area well (Suppl. Fig. 13b). The integrated MHW-month area combines the effects of MHW frequency, duration, and spatial extent, and therefore provides a comprehensive metric for evaluating the model performance. The close agreement between the simulated and observed time series of integrated MHW-month area supports the applicability of the model framework for investigating MHW-driven carbon cycle responses.

The globally integrated CO_2_ uptake by the coastal ocean simulated with ICON-Coast is 0.23 Pg C yr^−1^ for the period 2011–2020, which falls within the contemporary range of 0.18–0.45 Pg C yr^−1^ estimated from observations and models^3,7,8,118,119^. In terms of MHW-induced anomalies, the model reproduces the overall spatial pattern: most coastal regions are characterized by net upward *F*CO_2_ anomalies (Suppl. Figs. 3b, 14), consistent with observation-based estimates in low- and mid-latitude shelves (Fig. 1a and Suppl. Fig. 3). Towards higher latitudes, the magnitude of simulated *F*CO_2_ anomalies decreases, and in some regions the sign shifts to a net downward flux (Suppl. Fig. 3). Although this feature is qualitatively consistent with observational products, the spatial extent of downward anomalies is underestimated. Overall, the magnitudes of both net *F*CO_2_ anomalies and the individual contributions are generally weaker in the ICON-Coast model. It simulates +419 Tg C from thermal *p*CO_2,sea_ anomalies, –277 Tg C from non-thermal *p*CO_2,sea_ anomalies, and –125 Tg C from sea ice anomalies, compared to the observation-based estimates of +334 Tg C, –304 Tg C, and –85 Tg C, respectively. As a result, the integrated enhancement of coastal ocean CO_2_ uptake during MHW months inferred from observations is not fully reproduced by the model.

Despite these biases, the spatial distributions and zonal mean plots of thermal and non-thermal *p*CO_2,sea_ contributions show overall good qualitative agreement with observational estimates (Suppl. Figs. 15, 16). Moreover, the model simulates a coastal primary production of 5.0 Pg C yr^−1^ for 2001–2020, which is consistent with the satellite-derived range of 4.3–9.7 Pg C yr^−1^ (Suppl. Fig. 17). Both the primary production anomalies during MHW months (Suppl. Fig. 18) and the extent of sea ice cover (Suppl. Fig. 19) are realistically represented. On this basis, we employ the model to further quantify the non-thermal *F*CO_2_ contributions associated with anomalies in the circulation and biological carbon fixation by calculating the coastal carbon budget of the upper 50 m. In Fig. 4e–h, anomalies in the OC or IC stocks result from anomalies in river inputs, vertical sinking of particulate carbon, sedimentation of particulate carbon, decomposition of particulate and dissolved OC, net primary production, advective import of IC and advective export of OC (including anomalies due to circulation and vertical mixing), and CO_2_ uptake from the atmosphere. To account for model biases, we emphasize the relative contributions and provide absolute numbers for completeness.

## Supplementary information


Supplementary Information
Supplementary Dataset
Transparent Peer Review file


## Source data


Source Data


## Data Availability

The Segmentation dataset^120^ can be accessed at https://doi.org/10.5281/zenodo.8326096. The OISST v2.1 product is available from https://www.ncei.noaa.gov/products/optimum-interpolation-sst, and the OSTIA dataset^92^ is distributed by the Copernicus Marine Service at https://doi.org/10.48670/moi-00168. The MATLAB toolbox *m_mhw* is openly available at https://github.com/ZijieZhaoMMHW/m_mhw1.0. The ULB–SOM–FFN–coastalv2 dataset^121^ is provided by NOAA NCEI at https://doi.org/10.25921/4sde-p068. The CMEMS-LSCE-FFNN dataset^49^ is available from the Copernicus Marine Service at https://doi.org/10.48670/moi-00047. The OceanSODA-ETHZ dataset^122^ is archived at https://doi.org/10.25921/m5wx-ja34. The SeaFlux version 2021.04 dataset^123^ is available at https://doi.org/10.5281/zenodo.5482547. The CMEMS-LSCE product^62^ can be accessed at https://doi.org/10.14768/a2f0891b-763a-49e9-af1b-78ed78b16982. Source data are provided with this paper.

## Code availability

All code used to perform the analyses and generate the figures in this study is available via Zenodo at https://doi.org/10.5281/zenodo.21773605 (ref. ^124^). The ICON-Coast model code is available to individuals under specific license agreements (https://mpimet.mpg.de/en/science/modeling-with-icon/code-availability). By downloading the ICON-Coast source code, users agree to the terms of the corresponding license. The source code of ICON-Coast^125^ used in this study can be obtained from the Zenodo archive https://doi.org/10.5281/zenodo.6630353.
