# Supplementary material for: Polar and subpolar shelf seas dominate enhanced coastal CO2 uptake during marine heatwaves
Source: Nat Commun. 2026 Sep 1;17:9346. doi: 10.1038/s41467-026-77165-0 (PMC13534472; doi:10.1038/s41467-026-77165-0)
Supplement: Supplementary file 1 [file 41467_2026_77165_MOESM1_ESM.pdf]

Supplementary Information for

Polar and subpolar shelf seas dominate enhanced coastal CO_2 uptake during marine heatwaves

Zhentao Hu[1,2], Moritz Mathis[1,3], Hongmei Li[1,4], Tatiana Ilyina[1,2,4], Yoana G. Voynova[5], Vlad A. Macovei[5], Feng Zhou[6,7], Qicheng Meng[6,7], Corinna Schrum[1,2] & Wenyan Zhang[1]*

[1]Institute of Coastal Systems – Analysis and Modeling, Helmholtz-Zentrum Hereon, Geesthacht, Germany.

[2]Institute of Oceanography, Center for Earth System Research and Sustainability, University of Hamburg, Hamburg, Germany.

[3]Marine Sciences Department, Federal Maritime and Hydrographic Agency, Hamburg, Germany.

[4]Department of Climate Variability, Max Planck Institute for Meteorology, Hamburg, Germany.

[5]Department of Coastal Productivity, Institute of Carbon Cycles, Helmholtz-Zentrum Hereon, Geesthacht, Germany.

[6]State Key Laboratory of Satellite Ocean Environment Dynamics, Second Institute of Oceanography, Ministry of Natural Resources, Hangzhou, China.

[7]Observation and Research Station of Yangtze River Delta Marine Ecosystems, Ministry of Natural Resources, Zhoushan, China.

*Corresponding author: Wenyan Zhang (wenyan.zhang@hereon.de)

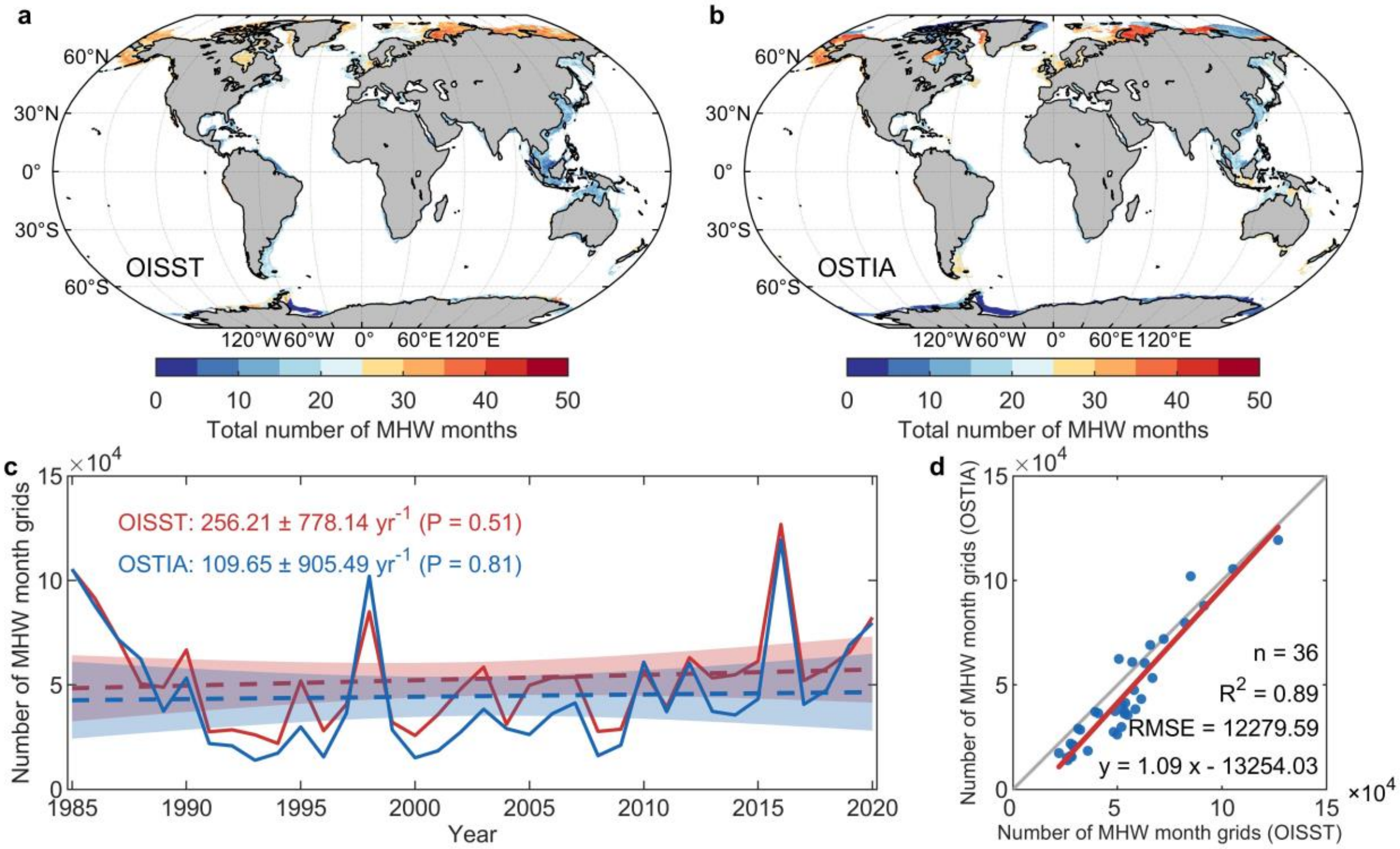

Supplementary Fig. 1 | Consistency in MHW month detection between OISST and OSTIA datasets (1985–2020). The global distribution of the total number of MHW months in coastal regions by the OISST dataset (**a**) and the OSTIA dataset (**b**). (**c**) Time series of the annual total number of grid cells identified as MHW months (OISST data in red and OSTIA data in blue). Dashed lines indicate linear trends, and shaded bands represent 95% confidence intervals. Trend slopes and corresponding *P* values are provided in the panel. (**d**) Scatterplot comparison of monthly grid cell counts between the two datasets. Data volume, R^2, RMSE, and regression lines are provided.

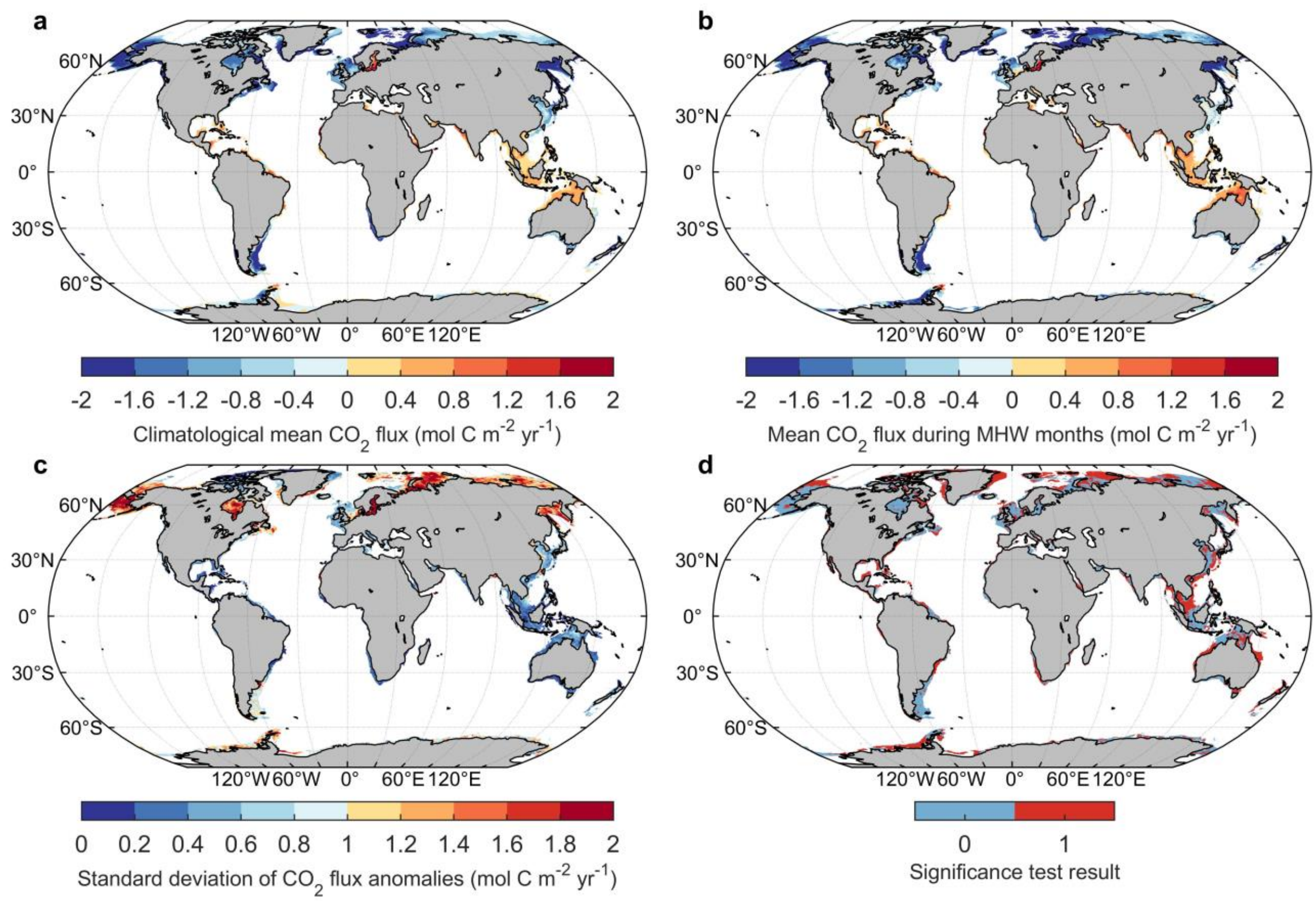

Supplementary Fig. 2 | Global coastal FCO$_2$ dynamics under MHWs: climatology, extremes, variability, and significance. Global coastal distributions of (**a**) the climatological mean FCO$_2$, (**b**) the mean FCO$_2$ during MHW months, (**c**) the standard deviation of FCO$_2$ anomalies, and (**d**) the results of statistical tests assessing whether mean FCO$_2$ anomalies differ significantly from zero (one-sample t-test or Wilcoxon signed-rank test depending on data normality, $P < 0.05$). In panels (**a**) and (**b**), positive (negative) FCO$_2$ values indicate CO_2 outgassing from the ocean to the atmosphere (CO_2 uptake by the ocean). All data shown are derived from the observation-based ULB–SOM–FFN–coastalv2 dataset[1].

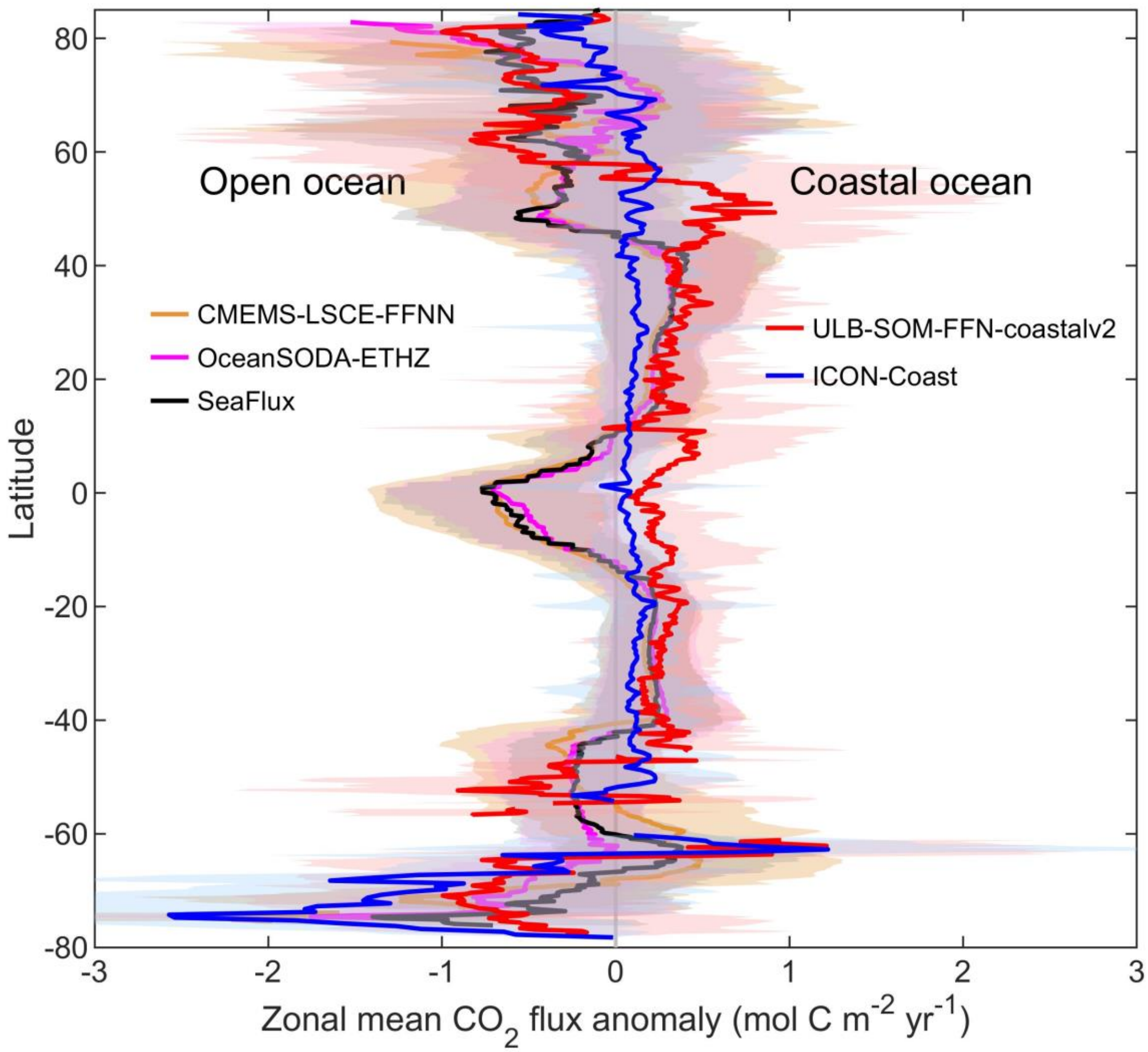

Supplementary Fig. 3 | Zonal mean CO_2 flux anomalies during MHW months (1985–2020) in the open ocean and coastal ocean. Zonal mean CO_2 flux anomalies are shown for the open ocean (derived from CMEMS-LSCE-FFNN, OceanSODA-ETHZ, and SeaFlux) and the coastal ocean (derived from ULB–SOM–FFN–coastalv2 and ICON-Coast). Only grid cells exhibiting statistically significant $F\mathrm{CO}_2$ anomalies are included in the zonal-mean calculation for both ocean domains. Positive anomalies indicate enhanced CO_2 outgassing or reduced uptake, whereas negative anomalies indicate enhanced uptake or reduced outgassing. Shading indicates ±1 standard deviation of the anomalies.

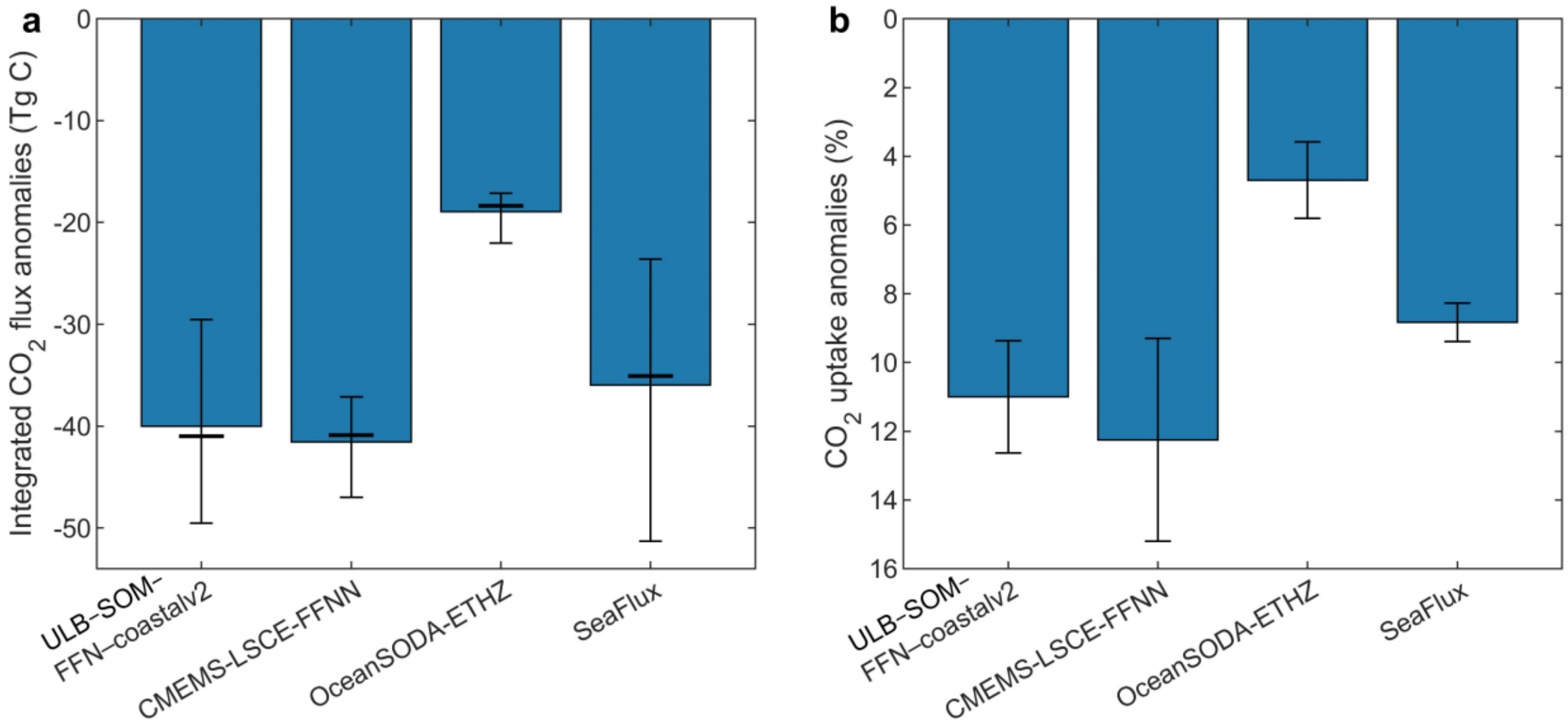

Supplementary Fig. 4 | Integrated *F*CO_2 anomalies and percentages associated with MHWs across datasets. Panel (**a**) shows integrated air–sea *F*CO_2 anomalies over the 1985–2020 period, and panel (**b**) presents the corresponding percentage anomalies induced by MHWs, based on four *F*CO_2 datasets: ULB–SOM–FFN–coastalv2, CMEMS-LSCE-FFNN, OceanSODA-ETHZ, and SeaFlux (Supplementary Table 1). Negative anomalies indicate enhanced oceanic CO_2 uptake during MHW months. Bars represent averages from eleven sensitivity tests. Black error bars indicate the full range in (**a**) and ±1 standard deviation in (**b**). Bold horizontal lines in (**a**) denote results using a 15-day MHW threshold. Percentage anomalies were calculated by normalizing the integrated anomalies against the corresponding integrated climatological fluxes.

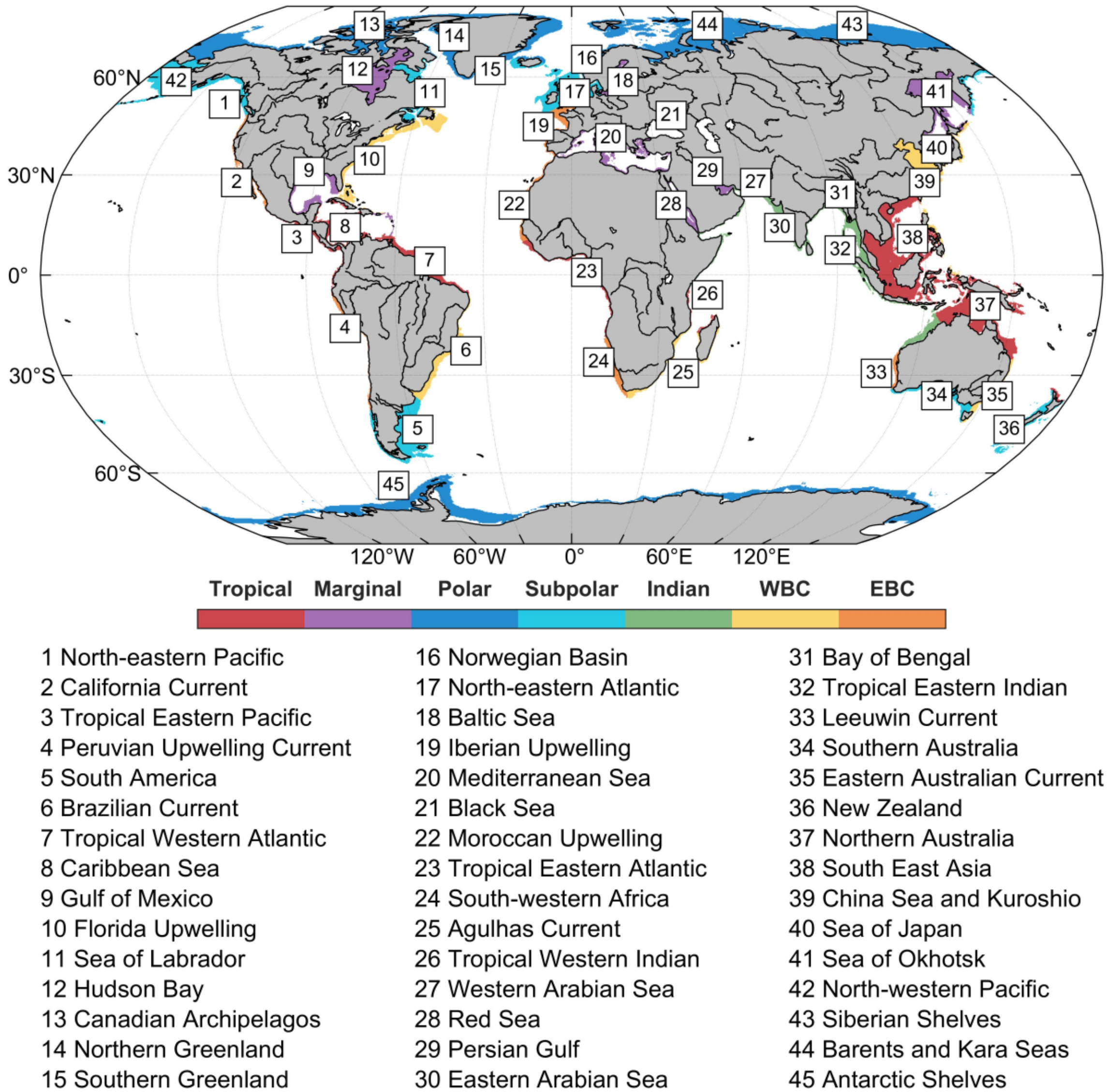

Supplementary Fig. 5 | Segmentation of the coastal ocean. Global continental shelf seas divided into 45 MARCATS regions[2], grouped into seven categories: eastern boundary currents (EBC), western boundary currents (WBC), Indian margins, subpolar margins, polar margins, marginal seas, and tropical margins. The figure is adopted from Roobaert et al.[3], with major rivers added for context.

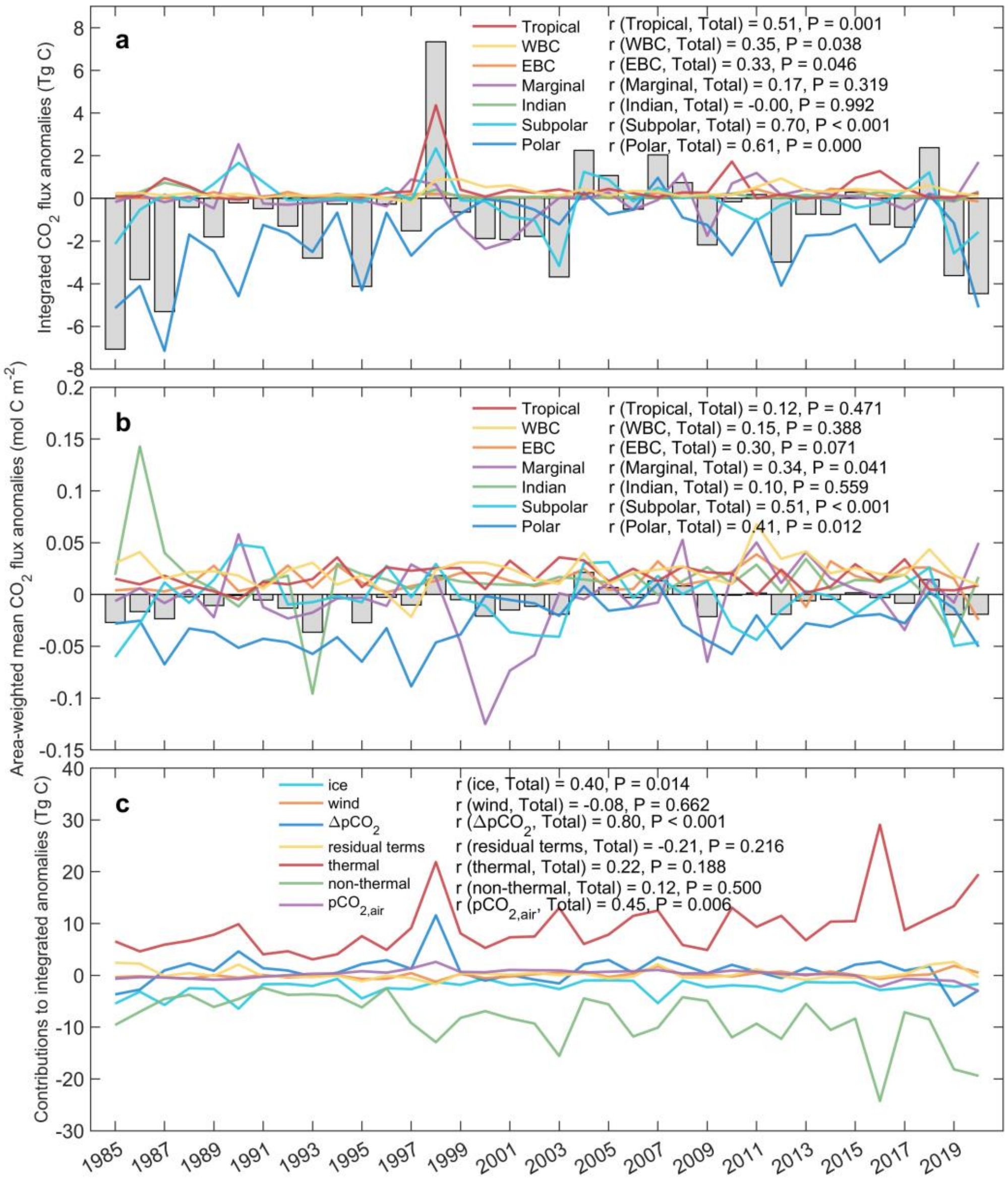

Supplementary Fig. 6 | Temporal dominance of regions and factors contributing to integrated FCO₂ anomalies. (**a**) Time series of integrated FCO_2 anomalies for the global coastal ocean (bars) and across seven distinct regional classes. Correlation coefficients and P values are provided. (**b**) same as (**a**), but for area-weighted mean FCO_2 anomalies. (**c**) Time series of integrated FCO_2 anomalies driven by seven factors, with correlation coefficients and P values indicated. Positive anomalies indicate contributions from enhanced CO_2 outgassing or reduced uptake, whereas negative anomalies indicate contributions from enhanced uptake or reduced outgassing. All data shown are derived from the observation-based ULB–SOM–FFN–coastalv2 dataset[1].

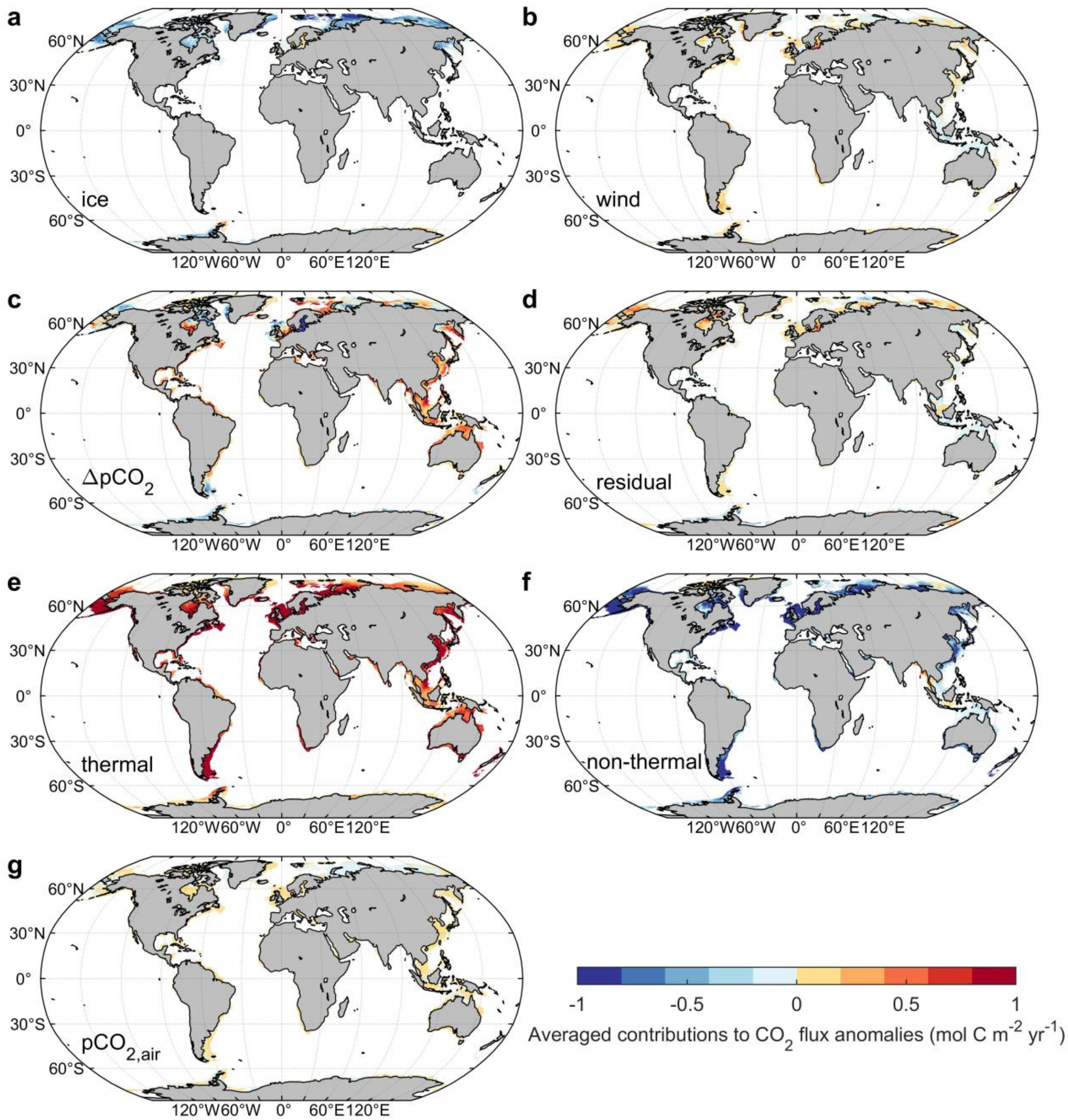

Supplementary Fig. 7 | Averaged contributions to FCO$_2$ anomalies. Global coastal distribution of the main drivers of FCO$_2$ anomalies (1985–2020), including sea ice content (**a**), wind speed (**b**), ΔpCO$_2$ (**c**), residual terms (**d**), thermal component of pCO$_{2,\mathrm{sea}}$ (**e**), non-thermal component of pCO$_{2,\mathrm{sea}}$ (**f**), and pCO$_{2,\mathrm{air}}$ (**g**). Positive anomalies indicate contributions from enhanced CO$_2$ outgassing or reduced uptake, whereas negative anomalies indicate contributions from enhanced uptake or reduced outgassing. All data shown are derived from the observation-based ULB–SOM–FFN–coastalv2 dataset[1].

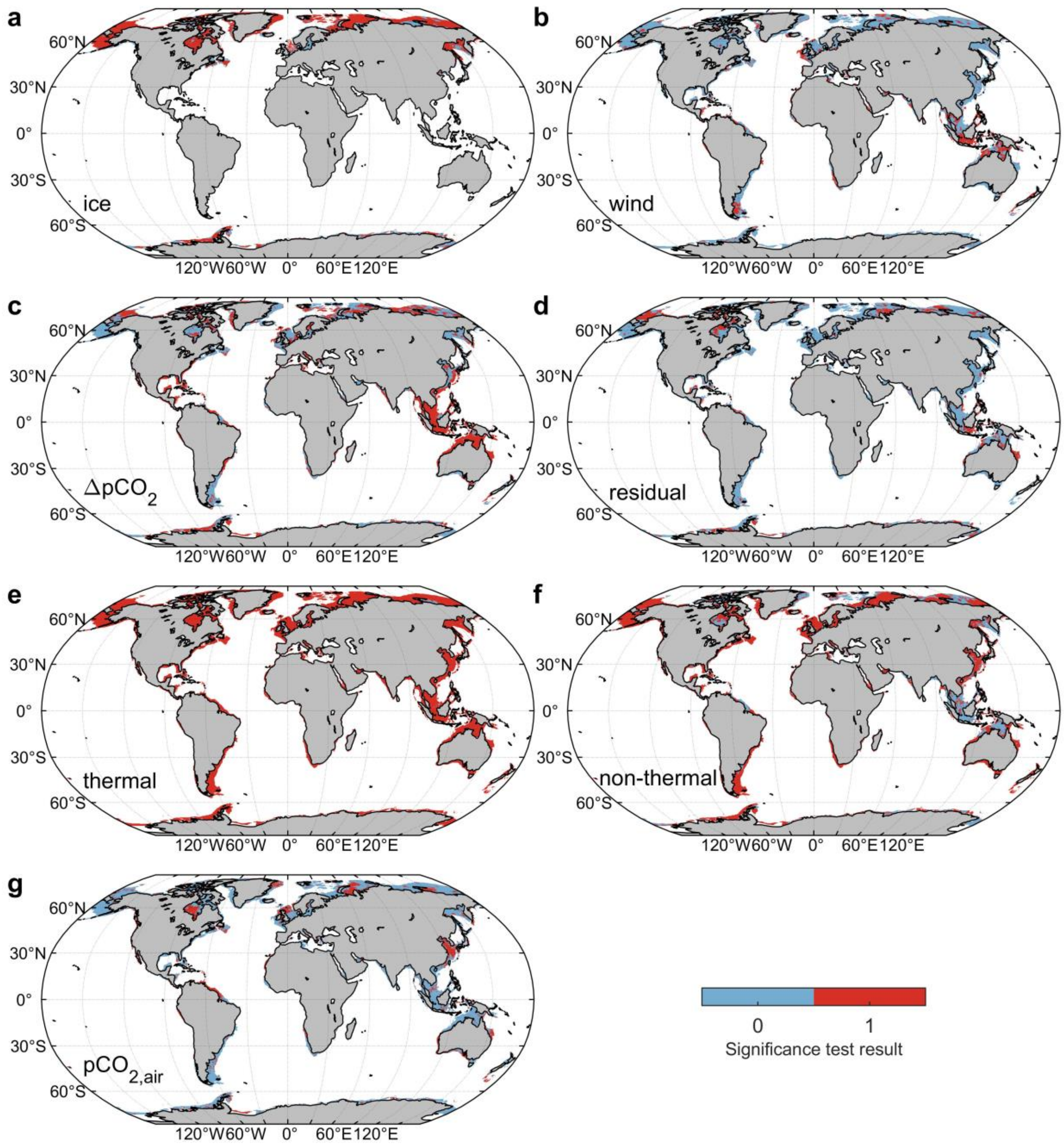

Supplementary Fig. 8 | Hypothesis test results for driver contributions (*h* values). Same as Supplementary Fig. 7 but showing the *h* values from statistical tests evaluating whether the contribution of each driver differs significantly from zero (one-sample t-test or Wilcoxon signed-rank test depending on data normality, $P < 0.05$). All data shown are derived from the observation-based ULB–SOM–FFN–coastalv2 dataset[1].

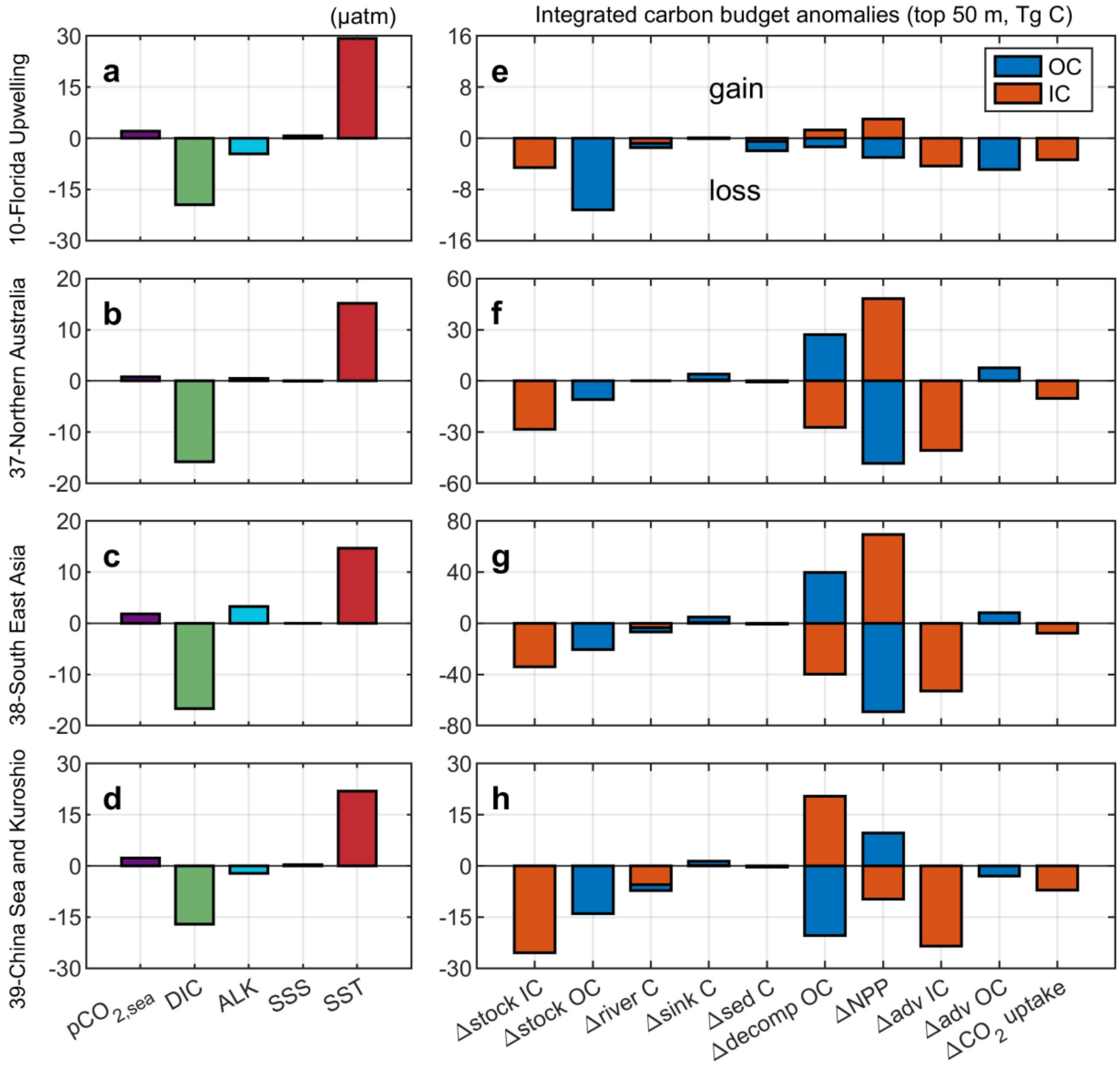

Supplementary Fig. 9 | Mechanisms driving non-thermal pCO$_{2,sea}$ anomalies and coastal carbon budget during MHW months from 1985 to 2020. Same as Fig. 4, but shown for (**a**,**e**) Florida Upwelling, (**b**,**f**) Northern Australia, (**c**,**g**) Southeast Asia, and (**d**,**h**) China Sea and Kuroshio.

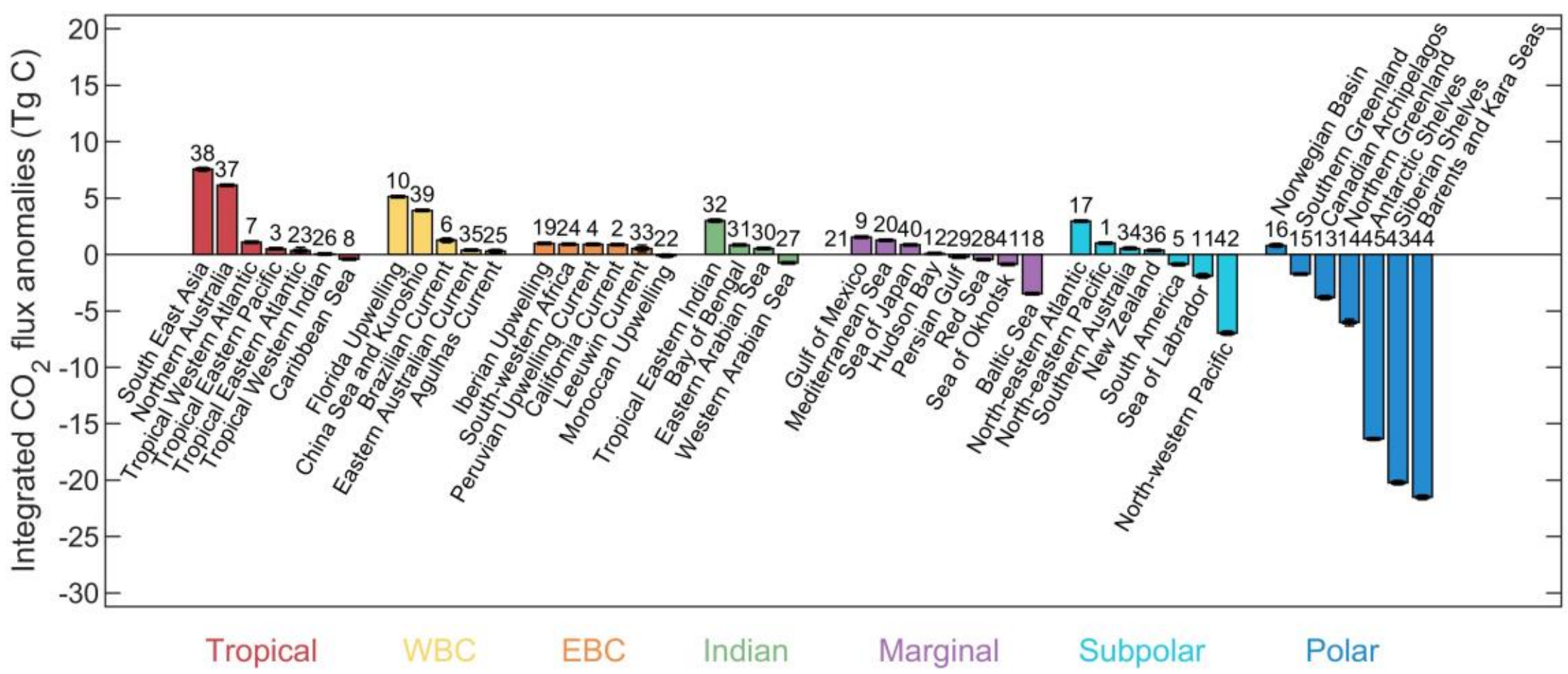

Supplementary Fig. 10 | Uncertainty of integrated coastal *F*CO_2 anomalies associated with reconstructed coastal *p*CO_2 RMSE. Bars represent the integrated CO_2 flux anomalies calculated using a 15-day threshold to define MHW months. Error bars represent uncertainties derived from the RMSE between reconstructed *p*CO_2 and SOCAT observations[1] for each of the 45 coastal segments[2]. Classes are ordered by total integrated CO_2 flux anomaly in descending order, with segments within each class further ordered by descending anomaly. Segment names and their serial numbers are shown.

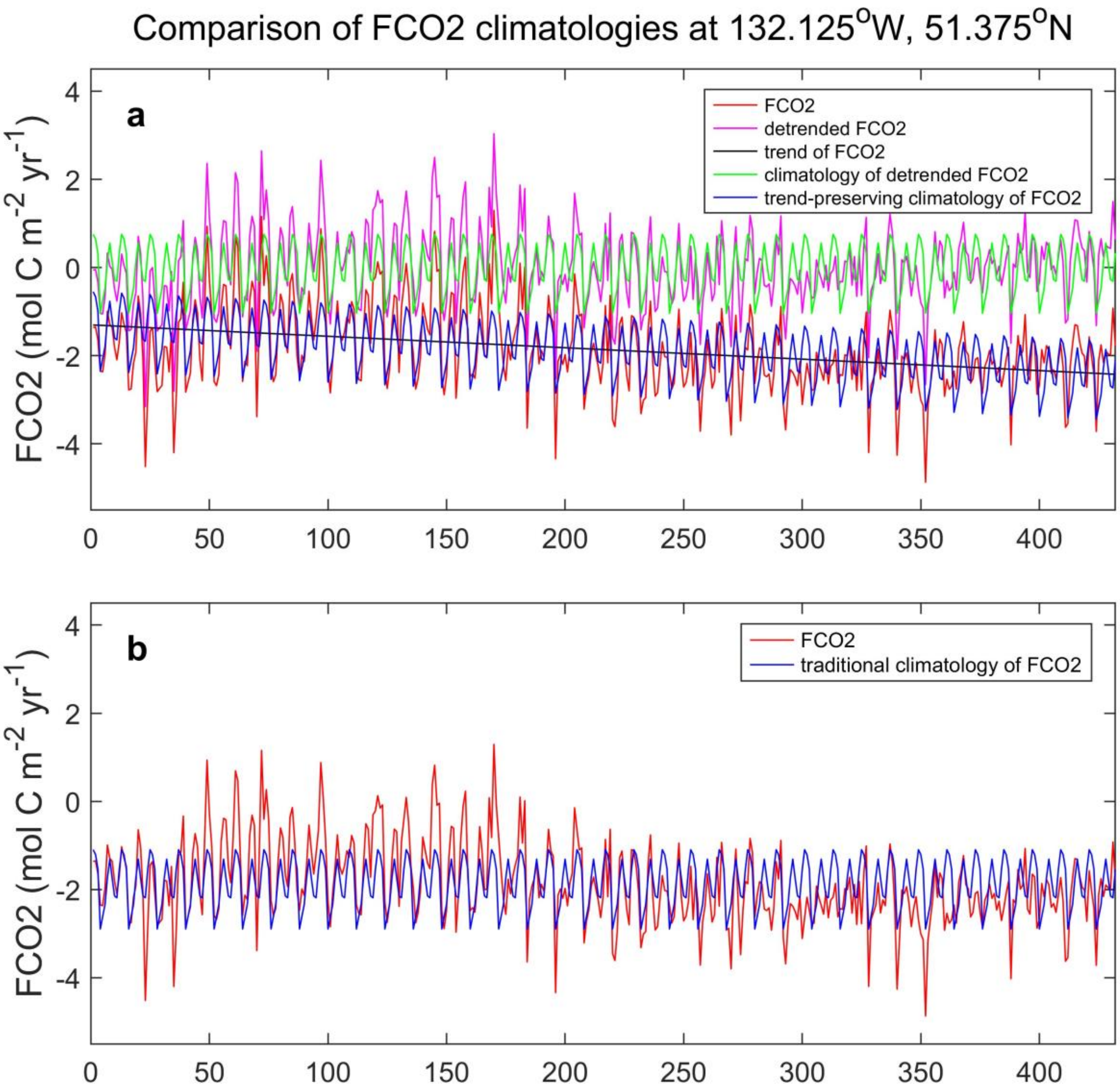

Supplementary Fig. 11 | Comparison of trend-preserving and traditional *F*CO_2 climatologies. **a**, Example of the trend-preserving climatology calculation for the *F*CO_2 time series at 132.125°W, 51.375°N derived from the ULB–SOM–FFN–coastalv2 dataset. The original *F*CO_2 time series (red) is first detrended (magenta), and the linear trend (black) is subsequently added back to the climatology of the detrended time series (green) to reconstruct the trend-preserving climatology (blue). **b**, Traditional climatology calculated directly from the original *F*CO_2 time series without detrending. This example illustrates the difference between the trend-preserving and traditional climatology approaches.

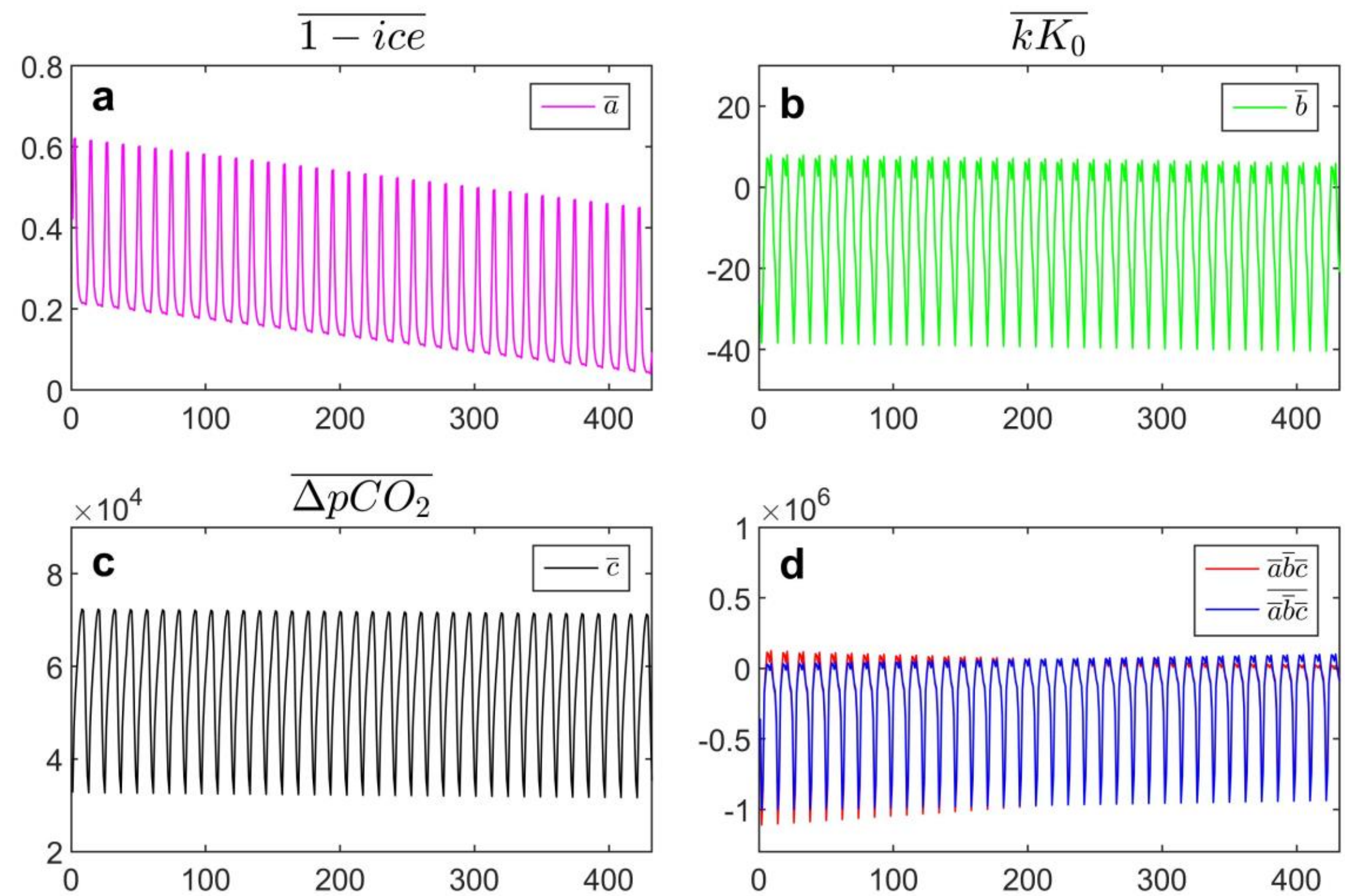

Supplementary Fig. 12 | Example illustrating the zero-order term in the Reynolds decomposition. Example of the Reynolds decomposition for FCO$_2$ at 109.375°W, 71.125°S derived from the ULB–SOM–FFN–coastalv2 dataset. **a**–**c**, Trend-preserving climatological states of $1 - ice$ ($\bar{a}$), kK_0($\bar{b}$), ΔpCO_2 ($\bar{c}$), respectively. **d**, Comparison between the product of the individual climatological states ($\bar{a}\bar{b}\bar{c}$) and the climatology of their product ($\overline{\bar{a}\bar{b}\bar{c}}$), illustrating that $\bar{a}\bar{b}\bar{c} \neq \overline{\bar{a}\bar{b}\bar{c}}$.

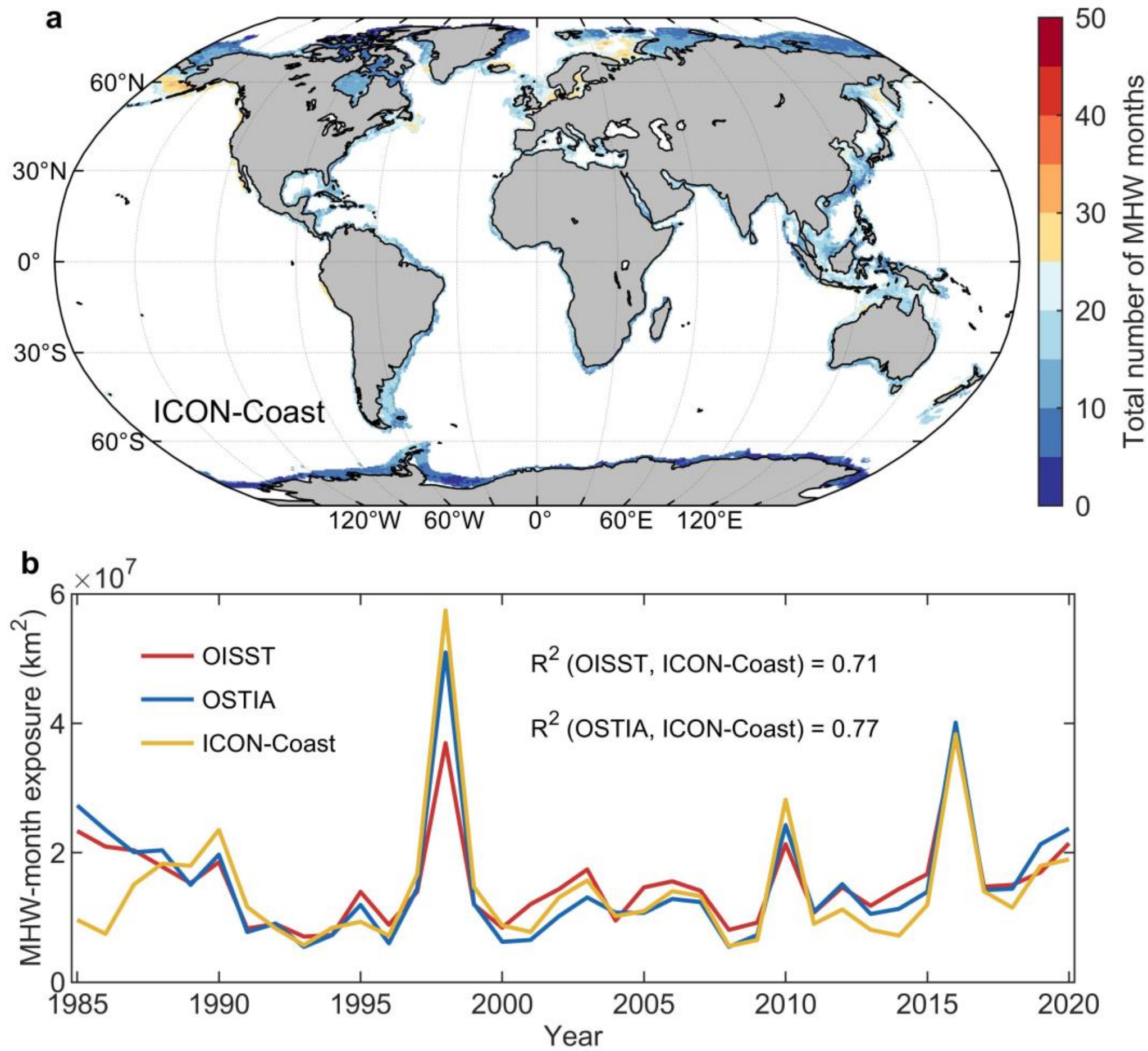

Supplementary Fig. 13 | Model evaluation of MHW-month metrics. (**a**) Global distribution of the total number of MHW months (1985–2020) in coastal regions simulated by ICON-Coast. (**b**) Time series of the integrated MHW-month area from OISST, OSTIA, and ICON-Coast, with R^2 values provided for comparisons between OISST and ICON-Coast, and between OSTIA and ICON-Coast.

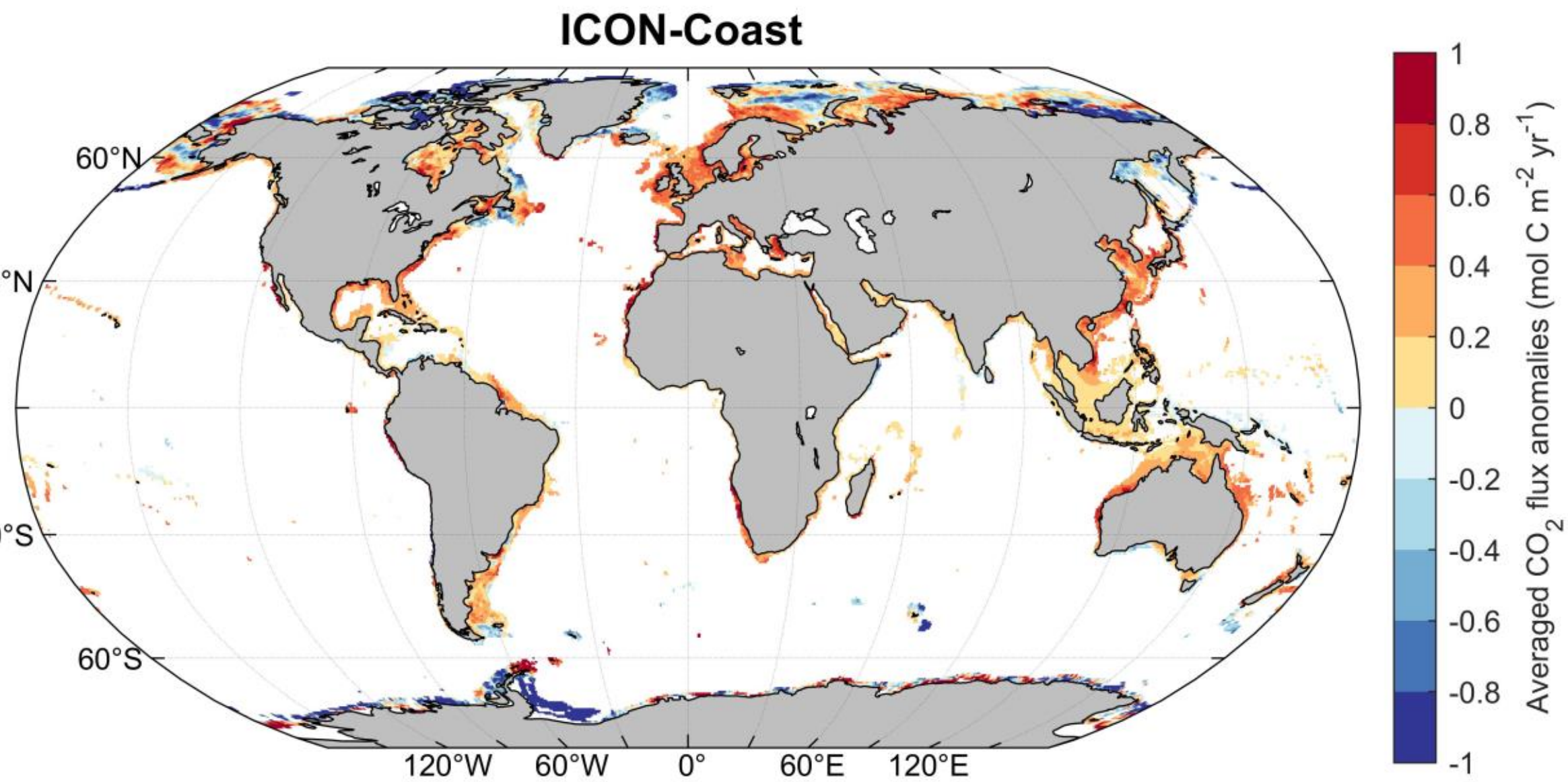

Supplementary Fig. 14 | Coastal $F$$CO_2$ responses to MHW months simulated by ICON-Coast. Spatial distribution of averaged air–sea $F$$CO_2$ anomalies (mol C m^{-2} yr^{-1}) across the global coastal regions, derived from ICON-Coast simulations.

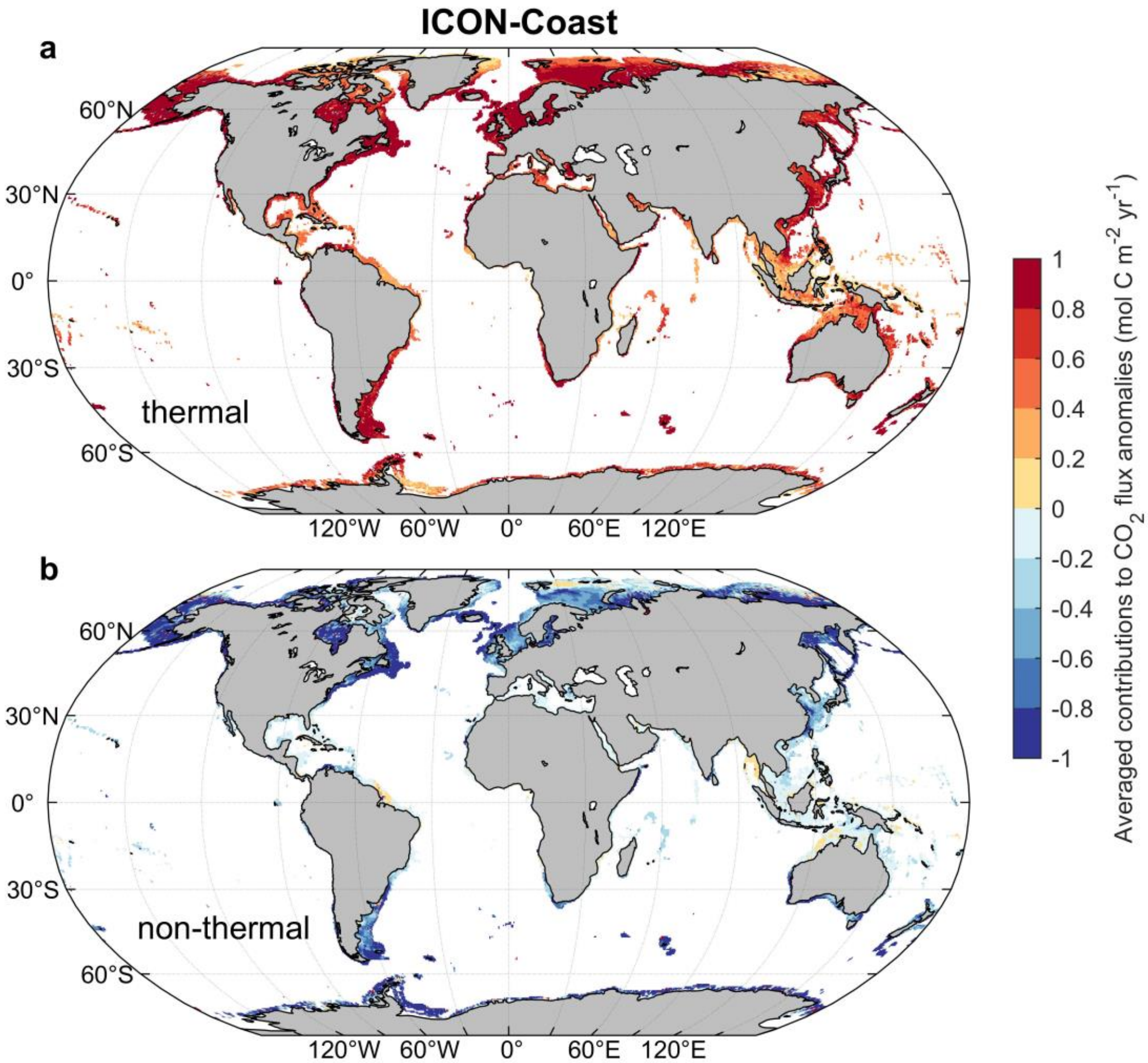

Supplementary Fig. 15 | Averaged contributions to $F$$CO_2$ anomalies simulated by ICON-Coast. Spatial distribution of thermal (**a**) and non-thermal (**b**) components of $p$$CO_{2,sea}$ contributing to air–sea $F$$CO_2$ anomalies (1985–2020), as simulated by ICON-Coast.

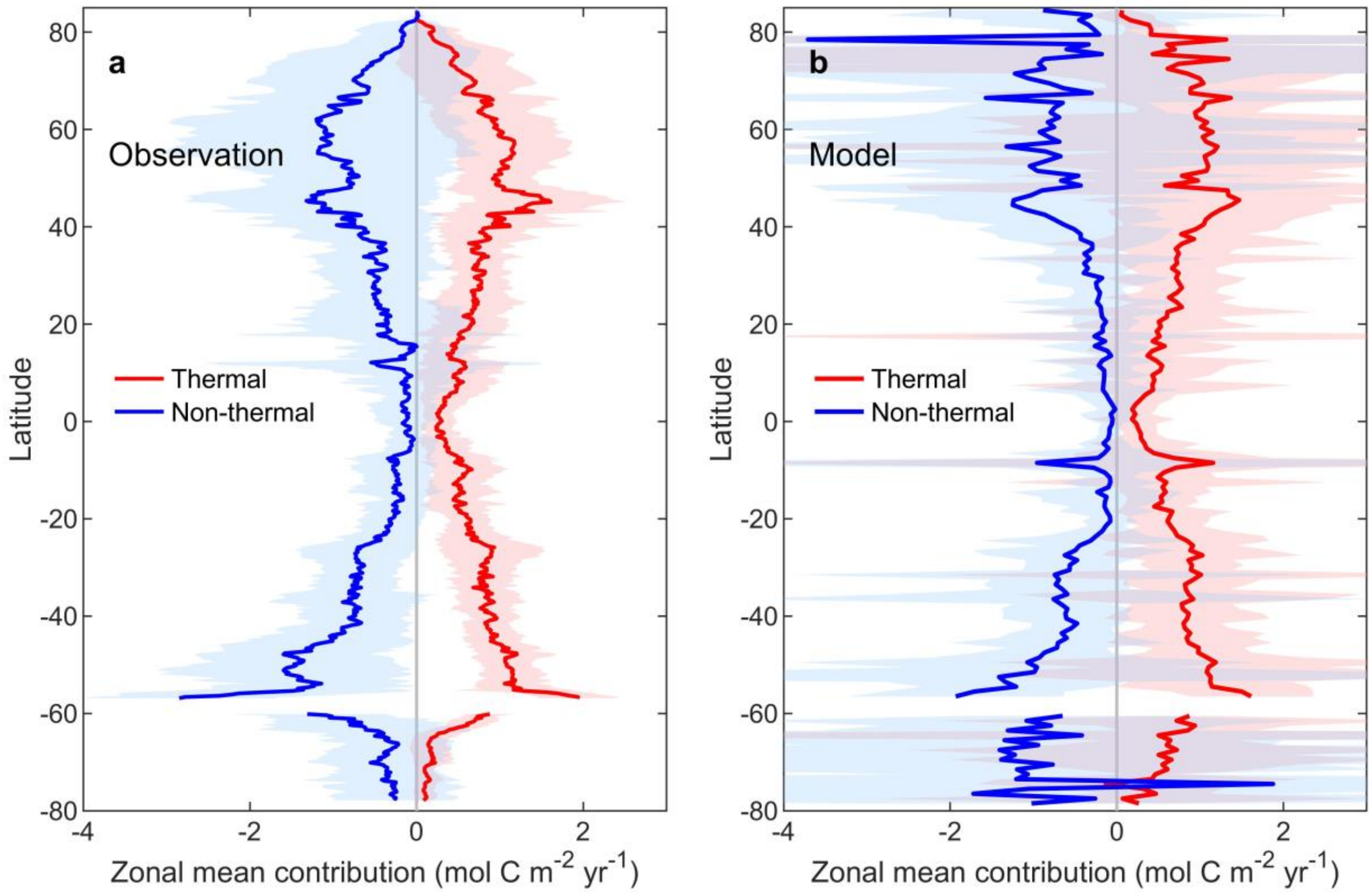

Supplementary Fig. 16 | Zonal mean contributions of thermal and non-thermal $pCO_{2,sea}$ components to FCO_2 anomalies during MHW months. (**a**) Results derived from ULB–SOM–FFN–coastalv2. (**b**) Results derived from ICON-Coast. Shading denotes ±1 standard deviation of the anomalies.

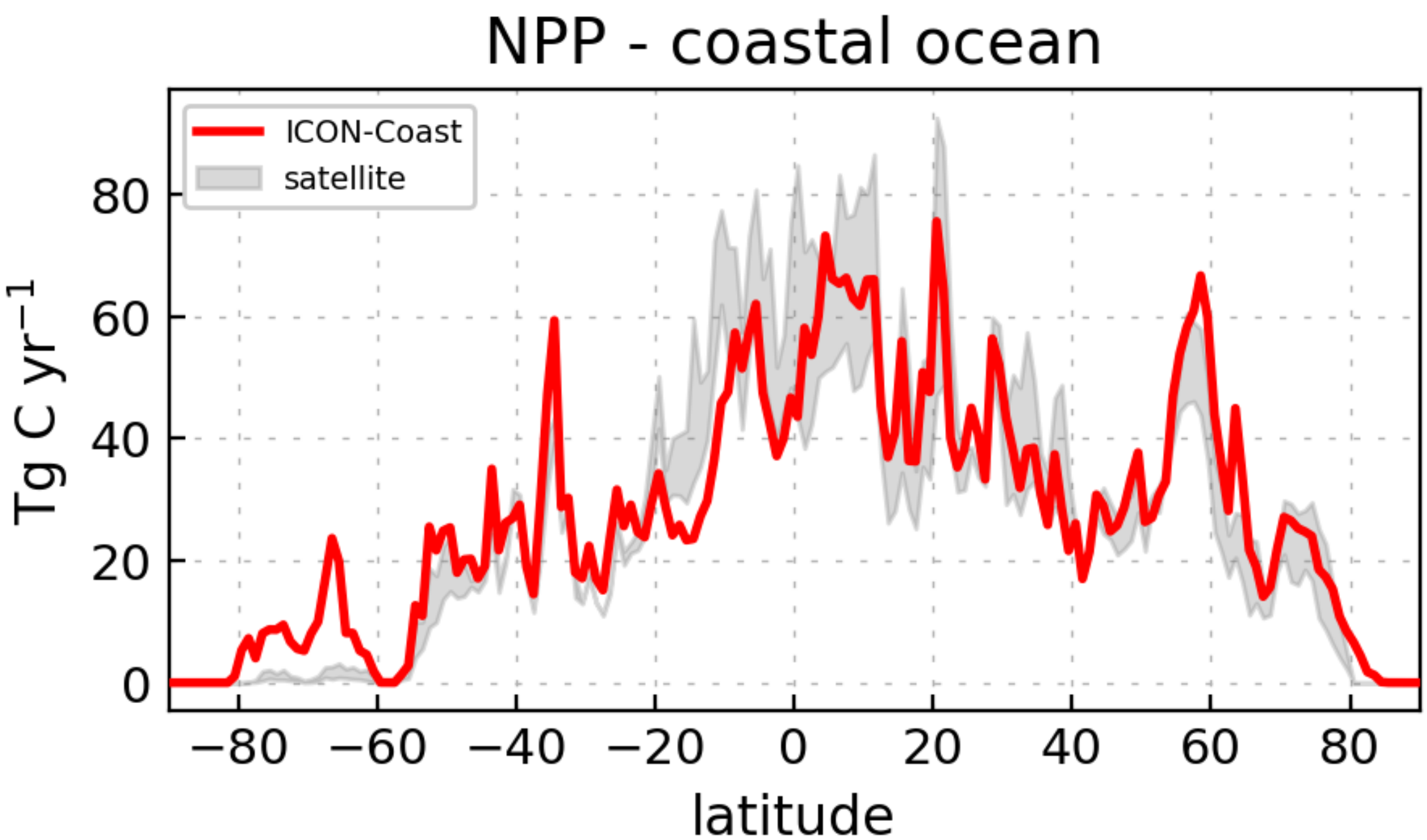

Supplementary Fig. 17 | Primary productivity in the coastal ocean. Zonally integrated annual net primary production in the coastal ocean, simulated by ICON-Coast (2001–2020, red) and calculated from the two satellite products (shaded grey) CBPM-VIIRS (2012–2020) and CAFE-MODIS (2003–2020).

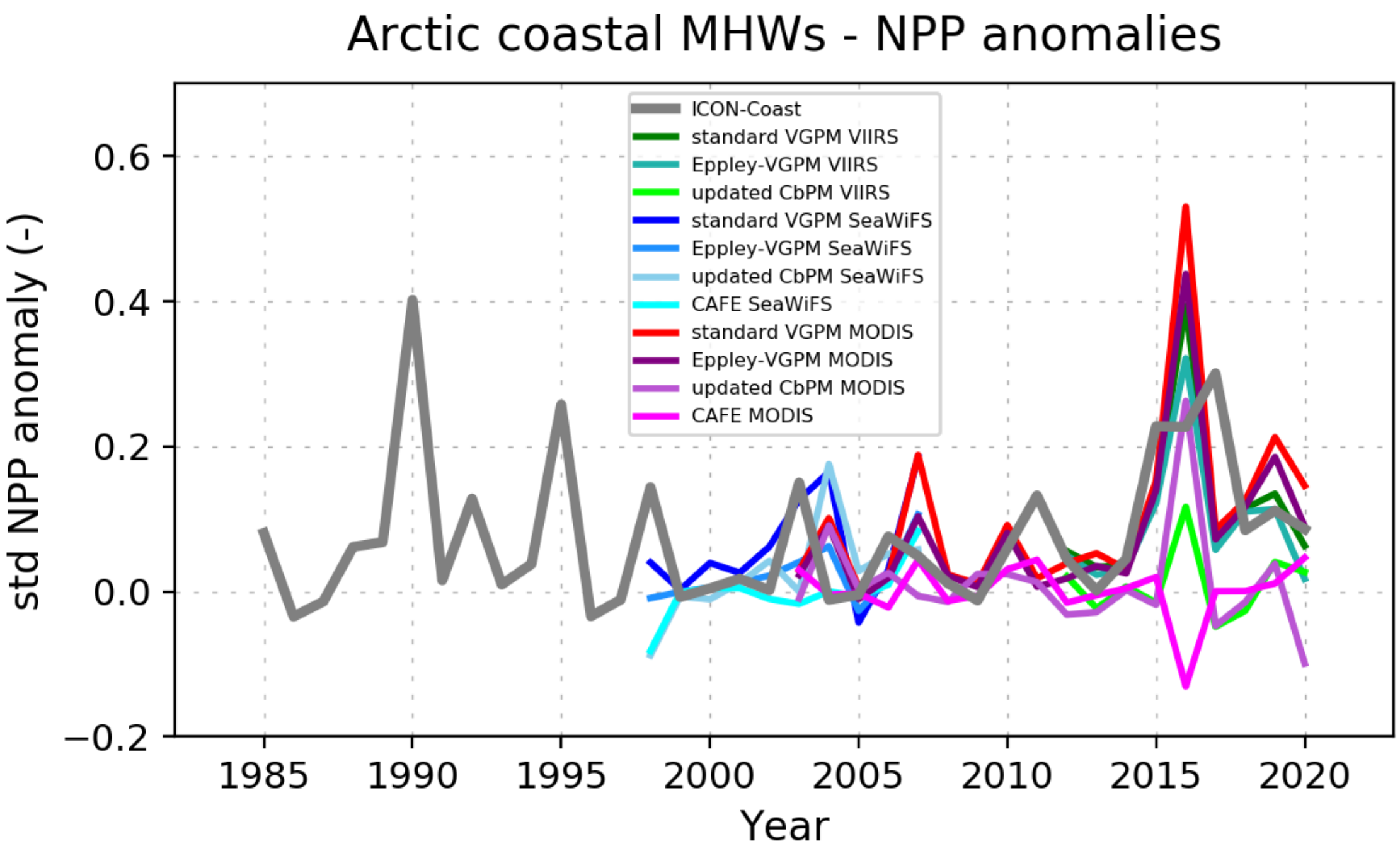

Supplementary Fig. 18 | Impact of MHWs on Arctic primary productivity. Standardized anomalies of annual net primary production (NPP) on Arctic shelves (>66°N) induced by MHWs, simulated by ICON-Coast (grey) and calculated from various satellite products named in the figure legend.

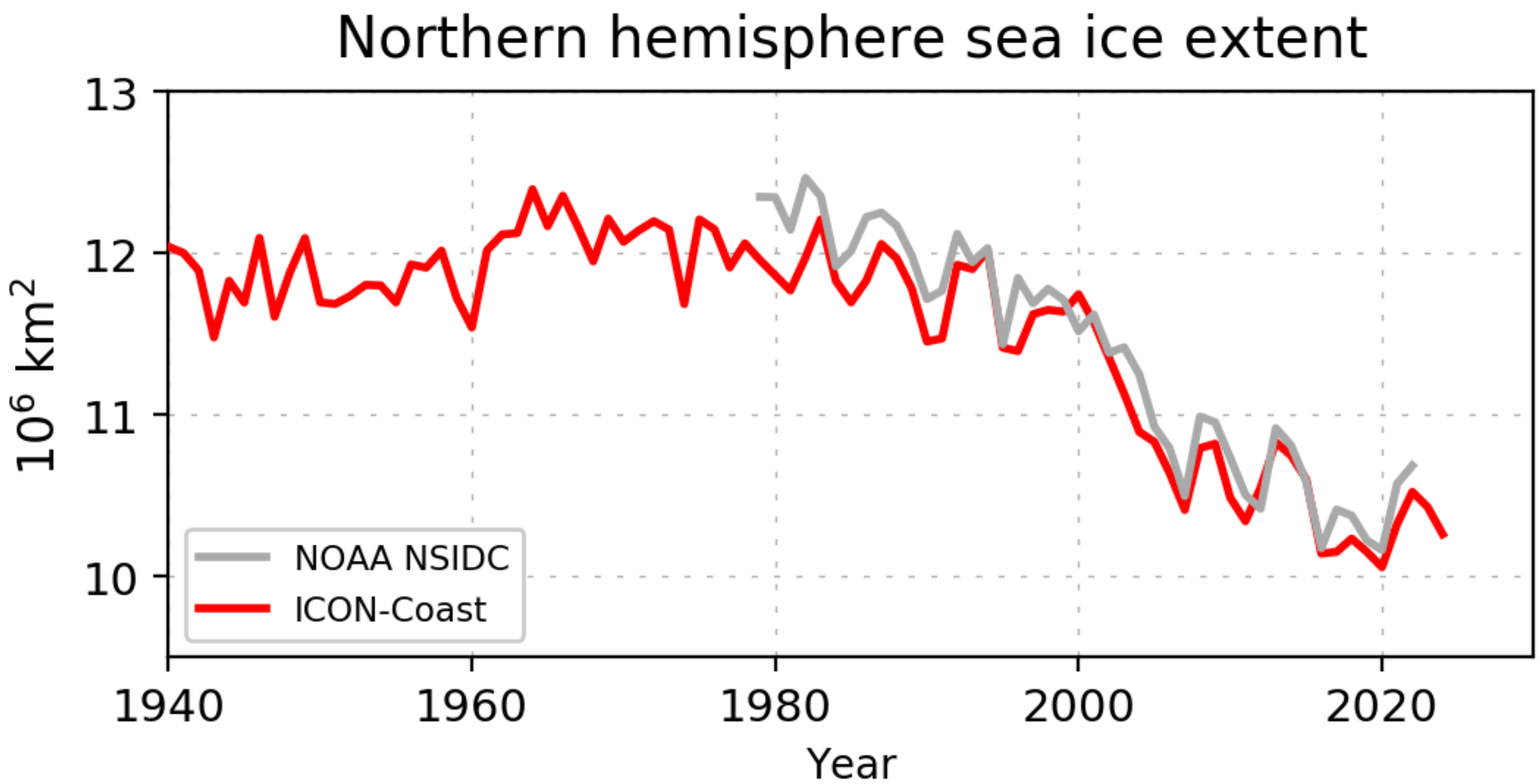

Supplementary Fig. 19 | Sea ice cover in the Arctic Ocean. Annual mean sea ice extent in the northern hemisphere, simulated by ICON-Coast (red) and compared with NOAA-NSIDC remote sensing observations.

Supplementary Table 1 | Details of FCO_2 and $pCO_{2,sea}$ products

**Product name**	**ULB–SOM–FFN–coastalv2**	**CMEMS-LSCE-FFNN**	**OceanSODA-ETHZ**	**SeaFlux version 2021.04**	**CMEMS-LSCE**
**Reference**	Roobaert et al. (2024) (ref. 1)	Chau et al. (2022) (ref. 4)	Gregor et al. (2021) (ref. 5)	Fay et al. (2021) (ref. 6)	Chau et al. (2024) (ref. 7)
**Interpolation method**	A two-step Self-Organizing Maps and Feed-Forward Network method	FFNN model is used to reconstruct pCO_2.	A Geospatial Random Cluster Ensemble Regression (GRaCER) method	Missing areas are filled using MPI-ULB-SOMFFN merged climatology adjusted by monthly ensemble-based scaling.	A modified CMEMS-LSCE-FFNN approach is used to reconstruct pCO_2. A locally interpolated alkalinity regression is used to estimate ALK.
**Available variables**	FCO_2; $pCO_{2,sea}$; ΔpCO_2; k; K_0; *ice*	FCO_2; $pCO_{2,sea}$; DIC; ALK	FCO_2; $pCO_{2,sea}$; k; K_0; *ice*; DIC; ALK; SST; SSS	FCO_2; $pCO_{2,sea}$; $pCO_{2,air}$; k; K_0; *ice*	$pCO_{2,sea}$; DIC; ALK; SST; SSS
**Temporal coverage**	1982–2020	1985–2024	1982–2022	1990–2019	1985–2021
**Resolution**	0.25° × 0.25°; monthly	0.25° × 0.25°; monthly	1° × 1°; monthly	1° × 1°; monthly	0.25° × 0.25°; monthly
**$pCO_{2,sea}$ training data**	SOCATv2022	SOCATv2020	SOCATv2019	SOCAT	SOCATv2022
**SST predictor**	OISST	OSTIA	OSTIA	OISST	OSTIA
**SSS predictor**	EN4	CMEMS	SODA	-	CMEMS
**Wind speed predictor**	ERA5	ERA5	ERA5	-	ERA5

**Sea ice predictor**	NOAA OISST	OSTIA	OSTIA	OISST	-
***p*CO** $\boldsymbol{pCO_{2,air}}$ **predictor**	xCO_2 from the ESRL surface MBL CO_2 product; ERA5 sea-level pressure; a vapor correction from Dickson (2007)	xCO_2 from CAMS	xCO_2 from NOAA: ATM; sea level pressure from ERA5	xCO_2 from the ESRL; ERA5 sea-level pressure; water vapor correction from Dickson (2007)	xCO_2 from CAMS
**Chlorophyll *a* predictor**	CMEMS	GLOCOLOUR	GLOCOLOUR	-	CMEMS

To ensure consistency across datasets, all datasets and predictor fields originally at 1° × 1° resolution were regridded to a uniform 0.25° × 0.25° resolution using grid-cell averaging before analysis. The SeaFlux version 2021.04 dataset integrates six global pCO_2 products with five wind reanalysis datasets, yielding 30 individual FCO_2 reconstructions. For our analyses, we used the ensemble mean of these reconstructions. Since each of the five datasets was derived from a corresponding SST dataset, the respective SST dataset was used to identify MHW months and to calculate anomalies for each dataset.

Supplementary Table 2 | Simulated anomalies in surface DIC and non-thermal $pCO_{2,sea}$ during MHW months for the representative MARCATS segments.

MARCATS	Surface DIC anomalies (mg C m^{-3})	Non-thermal $pCO_{2,sea}$ anomalies (µatm)
**10**	–9.1	–4.9
**37**	+1.7	–0.5
**38**	–7.0	–0.9
**39**	–17.4	–3.2
**42**	–24.0	–5.2
**43**	–37.0	–6.3
**44**	–3.2	–2.8
**45**	–37.4	–6.5

Values represent the mean anomalies averaged over all MHW months in each MARCATS segment.

Supplementary Note 1 | Uncertainty estimation of integrated FCO_2 anomaly during MHW months.

Following Roobaert et al.[1], two complementary sources of uncertainty were considered: (1) the reconstruction error of pCO_2, quantified by the RMSE between reconstructed pCO_2 and SOCAT observations, and (2) the uncertainty of the FCO_2 product, which accounts for uncertainties associated with ΔpCO_2, gas transfer velocity, wind speed, and sea-ice cover. These two components were used to estimate the uncertainty of the integrated FCO_2 anomaly during MHW months.

In three of the ten biogeochemical provinces (P1, P5, and P7), the RMSE exceeds 35 μatm[1]. Because the air–sea CO_2 flux is proportional to the air–sea pCO_2 difference (ΔpCO_2), uncertainties in pCO_2 translate directly into uncertainties in the estimated flux. The associated flux uncertainty can therefore be expressed as

$$\sigma_{FCO_2} = k \cdot K_0 \cdot (1 - ice) \cdot RMSE \quad (1)$$

Because the FCO_2 anomaly is defined as the difference between the instantaneous flux and its climatological value, uncertainties from both terms contribute to the anomaly uncertainty. Because both the instantaneous flux and its climatological value are derived from the same reconstructed product, they are expected to have comparable uncertainties. The resulting anomaly uncertainty can be approximated as

$$\sigma_{FCO_2,anom} = \sqrt{2} \cdot \sigma_{FCO_2} \quad (2)$$

For each grid cell and each MHW month, the anomaly uncertainty was converted to carbon units by multiplying by the grid-cell area (A_i) and converting from mol C to mass units. The total uncertainty associated with the integrated FCO_2 anomaly during MHW months was then obtained by combining grid-cell uncertainties under the assumption that errors are spatially independent:

$$\sigma_{total} = \sqrt{\sum_i (\sigma_{FCO_2,anom,i} \cdot A_i)^2} \quad (3)$$

In addition to reconstruction errors, we further considered the uncertainty of the integrated coastal air–sea CO_2 flux reported by Roobaert et al.[1], which is estimated to be 0.01 Pg C yr^{-1} for the global coastal domain. To apply this annual uncertainty to monthly estimates, we assumed that

uncertainties are temporally independent between months, with variances additive over time. Under this assumption, the variance of the monthly integrated flux can be expressed as

$$Var_{month} = \frac{(0.01)^2}{12} \quad (4)$$

Because MHW months occur only over a fraction of the coastal domain in each month, the uncertainty was further scaled according to the proportion of coastal area affected by MHW months. Let A_{MHW} denote the total area of grid cells experiencing MHW months during the 36-year study period, and A_{tot} the total coastal area. The variance associated with the integrated $F\mathrm{CO_2}$ anomaly is therefore

$$Var_{MHW} = Var_{month} \times \frac{A_{MHW}}{A_{tot}} \quad (5)$$

Since the $F\mathrm{CO_2}$ anomaly is defined as the difference between instantaneous flux and its climatological value, the variance of the anomaly becomes

$$Var_{anom} = 2 \times Var_{MHW} \quad (6)$$

The corresponding uncertainty of the integrated $F\mathrm{CO_2}$ anomaly is therefore

$$\sigma_{anom} = \sqrt{2 \times Var_{MHW}} \quad (7)$$

Finally, the robustness of the estimated $F\mathrm{CO_2}$ anomaly during MHW months was evaluated using the signal-to-noise ratio (SNR):

$$SNR = \frac{|F_{anom}|}{\sigma_{anom}} \quad (8)$$

where F_{anom} is the integrated $F\mathrm{CO_2}$ anomaly during MHW months and σ_{anom} is the corresponding uncertainty.

Supplementary References

1. Roobaert, A., Regnier, P., Landschützer, P. & Laruelle, G. G. A novel sea surface pCO_2-product for the global coastal ocean resolving trends over 1982–2020. *Earth Syst. Sci. Data* **16**, 421–441 (2024).
2. Laruelle, G. G. et al. Global multi-scale segmentation of continental and coastal waters from the watersheds to the continental margins. *Hydrol. Earth Syst. Sc.* **17**, 2029–2051 (2013).
3. Roobaert, A. et al. The spatiotemporal dynamics of the sources and sinks of CO_2 in the global coastal ocean. *Glob. Biogeochem. Cycles* **33**, 1693–1714 (2019).
4. Chau, T. T. T., Gehlen, M. & Chevallier, F. A seamless ensemble-based reconstruction of surface ocean pCO_2 and air–sea CO_2 fluxes over the global coastal and open oceans. *Biogeosciences* **19**, 1087–1109 (2022).
5. Gregor, L. & Gruber, N. OceanSODA-ETHZ: a global gridded data set of the surface ocean carbonate system for seasonal to decadal studies of ocean acidification. *Earth Syst. Sci. Data* **13**, 777–808 (2021).
6. Fay, A. R. et al. SeaFlux: harmonization of air–sea CO_2 fluxes from surface pCO_2 data products using a standardized approach. *Earth Syst. Sci. Data* **13**, 4693–4710 (2021).
7. Chau, T.-T.-T., Gehlen, M., Metzl, N. & Chevallier, F. CMEMS-LSCE: a global, 0.25°, monthly reconstruction of the surface ocean carbonate system. *Earth Syst. Sci. Data* **16**, 121–160 (2024).
