# Supplementary material for: Polar and subpolar shelf seas dominate enhanced coastal CO2 uptake during marine heatwaves
Source: Nat Commun. 2026 Sep 1;17:9346. doi: 10.1038/s41467-026-77165-0 (PMC13534472; doi:10.1038/s41467-026-77165-0)
Supplement: Supplementary file 2 [file 41467_2026_77165_MOESM3_ESM.pdf]

nature portfolio

Peer Review File

Polar and subpolar shelf seas dominate enhanced coastal CO2 uptake during marine heatwaves

Corresponding Author: Dr Wenyan Zhang

This file contains all reviewer reports in order by version, followed by all author rebuttals in order by version.

Version 0:

Reviewer comments:

Reviewer #1

(Remarks to the Author)
Review of NCOMMS-25-92847-T: “Polar and subpolar shelf seas dominate enhanced coastal CO2 uptake during marine heatwaves”

Overall assessment

This study analyzes observations and a model simulation to assess the effect of coastal marine heatwaves (MHWs) on air-sea carbon fluxes. Using a high-resolution coastal CO2 flux product, the authors show that MHWs increase net global coastal ocean carbon uptake by ca. 4%. This net effect arises from a negative (uptake) contribution from high latitudes, and a positive (outgassing) contribution from lower latitudes. The drivers (oceanic and atmospheric, thermal and non-thermal) of these fluxes are analyzed using observations and a model, showing that the non-thermal contribution is key.

Overall, this study presents novel and interesting results concerning the impact of MHWs on the carbon cycle. The findings contrast with previous results for the open ocean. The employed observational product and ocean model are well suited for this application. The text is well-written and easy to follow.

I recommend this article for publication in Nature Communications once the comments below have been addressed.

General comments

MHW months: it is understandable that the MHW detection has to be coarsened to “MHW months” due to the temporal resolution of FCO2 data. However, since the ICON model run in this study was presumably saved at daily resolution, it would be helpful to assess the impact of the temporal coarseness of the FCO2 data by reproducing the main results at daily resolution (e.g., doing composites of FCO2 anomalies during MHW days, not MHW months).

All figures: please state prominently in each Figure caption whether the data shown is from observations (Figs. 1-3) or from a model (Fig. 4). This includes the Extended Data figures

Model results: Figure 4e-h is the only part where the model output is analyzed, but is only directly discussed over the span of around 15 lines (267-272 and 279-286). This merits more discussion.

Model evaluation: in addition to the model’s skill in simulating carbon uptake and MHW-carbon coupling (as done on Ln. 702-726), the MHW statistics themselves should also be validated (frequency, duration, intensity, and impact of monthly averaging as mentioned above)

Net effect: it would be interesting to state the net effect not only relative to the global coastal ocean (4.3%), but also relative to the global ocean, to get a sense of the overall impact.

Specific comments & technical corrections

Ln. 24: “non-thermal DIC”: it could be more appropriate to say “non-thermal reductions in DIC”, since “thermal” does not refer to the DIC itself, but rather to the processes affecting it

Ln. 47: not sure if “constrained” is the right word here, maybe “perturbed”?

Ln. 54-57: the “coupling” introduced here is not very clear. MHWs are “coupled” to long-term ocean warming everywhere, not just in polar latitudes. The relation between MHWs and sea ice on the other hand is of course a polar phenomenon. The phrasing could be improved

Ln. 68-70: another question (and result of this study) is the net effect of coastal MHWs on carbon uptake, which is more quantitative than the heterogeneity of the response, which is to be expected

Ln. 96: “positive anomalies”: mention sign convention

Ln. 112: “segments are ordered by descending class”: reword this, e.g. “segments are ordered by descending mean (?) anomaly” or similar.

Ln. 115-117: it is not immediately clear why the high frequency of MHW months in the Arctic motivates the approach stated in the second part of the sentence

Ln. 152: “Equation (4)”: refer also to the Methods section

Ln. 166: “thermal effect” -> the thermal effect

Fig. 2: it would be helpful to add a title to panel 2a) (e.g. “Global”), since it is the most important of the small panels but hard to find at first

Fig. 3b, d: indicate the units

Ln. 226: “significant declines in negative integrated sea-ice anomalies”: the words “negative” and “anomalies” can both be removed without changing the meaning of the sentence (thus making it easier to understand). Replace “anomalies” by “area”

Ln. 234: “increase in sea ice loss”: sea ice area, not loss

Ln. 235: is the increasing sea ice trend in the Antarctic really “driven by a decline in MHW month frequency”? I would agree that they are consistent, but there are other factors driving sea ice trends, not just MHWs

Ln. 247: “the overall signs of their effects remain robust”: I don’t understand what this means, and how it proves the dominance of DIC? The impact of the approximate coefficients should be determined in a more quantitative way

Ln. 257: “IC and OC”: define these terms in the Figure caption

Fig. 4: it should be clearly indicated (e.g. in the first line of the figure caption) whether this is from model output or from observations. On line 246 you indicate that panels a-d are from CMEMS-LSCE, while panels e-h are from ICON (Ln. 268). This is not clear at all from looking at the figure

Ln. 308: “small coastal carbon sink”: you previously stated the the coastal carbon sink is disproportionately large?

Ln. 311: “strength” -> strengthen

Ln. 317: “despite uncertainties remain”: check grammar
Extended Data: the last few figures are not rendered correctly and lack a caption

Eq. 4: it looks like some of the “ice” variables are rendered with a different letter “i” (someone didn’t “dot their i’s” as they say...)

(Remarks on code availability)

Reviewer #2

(Remarks to the Author)
Dear Authors,

your paper is interesting, and mostly talks about trends in some pCO2 and air-sea CO2-flux products (not necessarily marine heat waves, the way they are described).

However, I have a few major comments.

1: how heat waves are defined here seem to me to unfortunately mix up different things (if I am not mistaken). It is applied to monthly averages and there are two main criteria used. In regions where there are large positive trends, the first one will select only months in the warm (recent) part of the time series (that's very clear from the supp mat figures) (a month kept if it have more than 15 days of heat wave day, which is also a strange criteria). The second one tries to minimize that, byt only considering months that stand out 'positive' in the detrended time series. At the end, I dont understand what we are looking at, and I really dont see the added value of having done the large effort of defining a heat wave, etc... from daily SST field. I would like to get convinced that we would not get basically the same results by simply looking at monthly fields and anomalies, and what the added complexity brings us.I personnally feel that the heat wave should be defined in detrended time series. One could at the end look at months with similar criteria which would pass the criterium of the 15 days ... ANd there is anyway a caveat which is that the reconstructed pCO2 products are monthly (and rightly so, as the construction method is eally geared to relatively low frequencies). How will you be confident that this is a quasi linear system relative to heat wave conditions?

2: the analysis is based on a heterogeneous set of products which are poorly described in the paper, and it is a bit hard to get in particular at what are there respective advantages and issues based on what is written in the Supp mat. Also, among those five products, four are explicitely described as extrapolating offshelf pCO2 (fluxes) to the coastal and shelf regions (which is not always the case, actually in those, such as for the Barents Sea). For that reason, they are considered 'inferior' to the fifth product (by Roobaert et al., 2024), but are nonetheless included, which in some ways is a bit contradictory. It is also not clear how the information from the different products are merged (or is it only in Supp Mat). Furthermore, Roobaert et al explicitely mentions the issue of sampling and potential biases and errors in regions with partial or seasonal ice cover (ice rate change been a key parameter in the training and construction of the product) (see the comments on regions 1, and 7). In many ways, these are the regions where the largest impact of the trends (I'll stop calling that heat waves!) is found.

3: part of the reason why regions with partial ice cover are more uncertain (OK: except parts of the Arctic, where the change in time is major...) is that there are not that many data in many of those places and how one defines the sea ice mask has strong implications on the reconstructions... Notice also that SST products have also rather large uncertainties in some of these regions...

Now, I dont have particular objections to what is stated on explaining the changes and it is all logical with the way the products are constructed, and to current knowledge, but that's related to a good extent (for the Arctic for example) to the low frequency pluri-annual variability/trends.

I would suggest that the paper with revisited. If the emphasis is on heat waves (even if combined in monthly categories), then do it in a coherent way which excludes the trends in SST during the selection, and compare those months with non-heat wave month for the same part of the time series. There might be interesting trends in that too! Or, adopt a low frequency approach directly based on the monthly means.

Minor comments:
311: to further strengthen…
318: Despite remaining uncertainties

(Remarks on code availability)

Reviewer #3

(Remarks to the Author)

(Remarks on code availability)

Version 1:

Reviewer comments:

Reviewer #1

(Remarks to the Author)
My comments from the previous round of reviews have been satisfactorily addressed.

(Remarks on code availability)

Reviewer #2

(Remarks to the Author)

I thank the authors to have made a major revision, and to have clearly separate trends from HW events.
I find the detailed response and the revised manuscript and supp. Mat. really appropriate.

One very small comment:

Line460, when mentioning y=abc as a framework, indicate that this is a good framework for equation (1), where y is FCO2…
(I belive that lines 472-475 are fine where they are)

(Remarks on code availability)

Reviewer #3

(Remarks to the Author)

(Remarks on code availability)

Version 2:

Reviewer comments:

Reviewer #3

(Remarks to the Author)

(Remarks on code availability)

Version 3:

Reviewer comments:

Reviewer #3

(Remarks to the Author)

(Remarks on code availability)

Open Access This Peer Review File is licensed under a Creative Commons Attribution 4.0 International License, which permits use, sharing, adaptation, distribution and reproduction in any medium or format, as long as you give appropriate credit to the original author(s) and the source, provide a link to the Creative Commons license, and indicate if changes were made.
In cases where reviewers are anonymous, credit should be given to 'Anonymous Referee' and the source.
The images or other third party material in this Peer Review File are included in the article's Creative Commons license, unless indicated otherwise in a credit line to the material. If material is not included in the article's Creative Commons license and your intended use is not permitted by statutory regulation or exceeds the permitted use, you will need to obtain permission directly from the copyright holder.
To view a copy of this license, visit https://creativecommons.org/licenses/by/4.0/

Response to the reviewers' comments

We sincerely thank the reviewers for their thorough evaluation of our manuscript and for their constructive comments and suggestions, which have significantly improved the quality of this work. Below, we provide point-by-point responses to all comments (cited in black). Our responses are presented in blue, and the corresponding revisions in the manuscript are highlighted in italic and underlined text.

Reviewer #1 (Remarks to the Author):

Review of NCOMMS-25-92847-T: "Polar and subpolar shelf seas dominate enhanced coastal CO2 uptake during marine heatwaves"

Overall assessment

This study analyzes observations and a model simulation to assess the effect of coastal marine heatwaves (MHWs) on air-sea carbon fluxes. Using a high-resolution coastal CO2 flux product, the authors show that MHWs increase net global coastal ocean carbon uptake by ca. 4%. This net effect arises from a negative (uptake) contribution from high latitudes, and a positive (outgassing) contribution from lower latitudes. The drivers (oceanic and atmospheric, thermal and non-thermal) of these fluxes are analyzed using observations and a model, showing that the non-thermal contribution is key.

Overall, this study presents novel and interesting results concerning the impact of MHWs on the carbon cycle. The findings contrast with previous results for the open ocean. The employed observational product and ocean model are well suited for this application. The text is well-written and easy to follow.

I recommend this article for publication in Nature Communications once the comments below have been addressed.

Response: We sincerely thank the reviewer for the positive and encouraging evaluation of our work. In the revised manuscript, SST has been detrended prior to MHW detection. Although detrending SST alters the time-series figures and largely removes the long-term trend, the main

conclusions remain robust, with only moderate quantitative adjustments, such as the change in the integrated CO_2 flux anomaly from 4.3 ± 0.2% to 11.0 ± 1.6%. Detailed point-by-point responses are provided below.

General comments

MHW months: it is understandable that the MHW detection has to be coarsened to "MHW months" due to the temporal resolution of FCO2 data. However, since the ICON model run in this study was presumably saved at daily resolution, it would be helpful to assess the impact of the temporal coarseness of the FCO2 data by reproducing the main results at daily resolution (e.g., doing composites of FCO2 anomalies during MHW days, not MHW months).

Response: Daily model output of SST is indeed available, and we have therefore updated the entire model analysis using MHWs detected from daily model SST. However, daily outputs of FCO_2 and related variables were not saved in the model output. In addition, extending the analysis strictly to MHW days may interfere with the main scientific message of this study.

The net effects of MHWs on FCO_2 or other biogeochemical metrics are not necessarily confined to periods of anomalously high SST; they may persist beyond the event itself or even reach maximum effect after the MHWs[1,2]. Therefore, using MHW months provides a more realistic, general, and inclusive representation of the carbon flux response than limiting the analysis to days with high SST only. This relatively low-frequency approach is consistent with previous studies[3,4] and captures both the instantaneous and delayed responses of CO_2 fluxes to MHWs.

We note that the exact duration and magnitude of these delayed responses remain uncertain and may vary across processes and regions, representing an open question that warrants an in-depth investigation in future studies. A systematic comparison of daily versus monthly responses is therefore beyond the scope of this study.

All figures: please state prominently in each Figure caption whether the data shown is from observations (Figs. 1-3) or from a model (Fig. 4). This includes the Extended Data figures

Response: We thank the reviewer for the comment. All figure captions, including those in the Supplementary Information, have been revised to clearly indicate whether the data are

observational or model-based, using phrases such as "*All data shown are derived from the observation-based ULB–SOM–FFN–coastalv2 dataset*", "*derived from the observation-based CMEMS-LSCE dataset*", or "*based on the ICON-Coast model*", ensuring that this information is clear to readers.

Model results: Figure 4e-h is the only part where the model output is analyzed, but is only directly discussed over the span of around 15 lines (267-272 and 279-286). This merits more discussion.

Response: We thank the reviewer for highlighting this point. To address it, we have expanded the discussion on how MHW-induced changes in upper-ocean stratification affect phytoplankton, as follows (Page 10, Lines 257–268): "*Phytoplankton, as a key driver of carbon fixation in marine ecosystems, plays an important role in carbon cycling[76]. Its response to MHWs can propagate through higher trophic levels, influencing zooplankton, fish, seabirds, and marine mammals[77]. The impact of MHWs on phytoplankton shows a clear latitudinal pattern, with chlorophyll generally declining in tropical and mid-latitude regions but increasing at high latitudes[78]. In nutrient-rich polar and subpolar regions with deep mixed layers, MHWs can directly alter upper-ocean stratification[47,49,78,79], modifying light and nutrient availability and extending the residence time of phytoplankton in the euphotic zone[77]. Recent MHWs in the subpolar North Pacific have been associated with shifts in dominant phytoplankton functional groups[80,81]. In the Antarctic coastal ocean, MHWs can enhance stratification and trigger iron release from melting sea ice, fertilizing waters that are high in nutrients but low in chlorophyll, stimulating phytoplankton growth along the ice edge, and thereby enhancing CO_2 uptake[49,82].*"

Model evaluation: in addition to the model's skill in simulating carbon uptake and MHW-carbon coupling (as done on Ln. 702-726), the MHW statistics themselves should also be validated (frequency, duration, intensity, and impact of monthly averaging as mentioned above)

Response: As our analysis focuses on MHW months, we additionally evaluate the simulated MHW characteristics against OISST/OSTIA estimates by comparing the spatial distribution of MHW-month occurrence and the temporal variability of the integrated annual MHW-month area. The integrated MHW-month area combines the effects of MHW frequency, duration, and spatial extent.

The following text is added to the Model Evaluation section (Page 21, Lines 557–570): “*MHW statistics are evaluated by comparing the simulated spatial distribution of MHW-month occurrence and the temporal variability of the integrated annual area affected by MHW months against OISST/OSTIA-based estimates. Overall, the model reproduces the global spatial distribution of MHW-month occurrence reasonably well, although the total frequency of MHW months in high-latitude regions is generally underestimated compared to observations (Supplementary Fig. 11a). This underestimation is likely related to the relatively thick uppermost model layer (~16 m), which dampens near-surface temperature extremes and reduces the occurrence of simulated MHWs relative to satellite-derived SST products representing the ocean skin layer. Despite these regional biases, the model reproduces the interannual variability of the integrated MHW-month area well (Supplementary Fig. 11b). The integrated MHW-month area combines the effects of MHW frequency, duration, and spatial extent, and therefore provides a comprehensive metric for evaluating the model performance. The close agreement between the simulated and observed time series of integrated MHW-month area supports the applicability of the model framework for investigating MHW-driven carbon cycle responses.*”

“

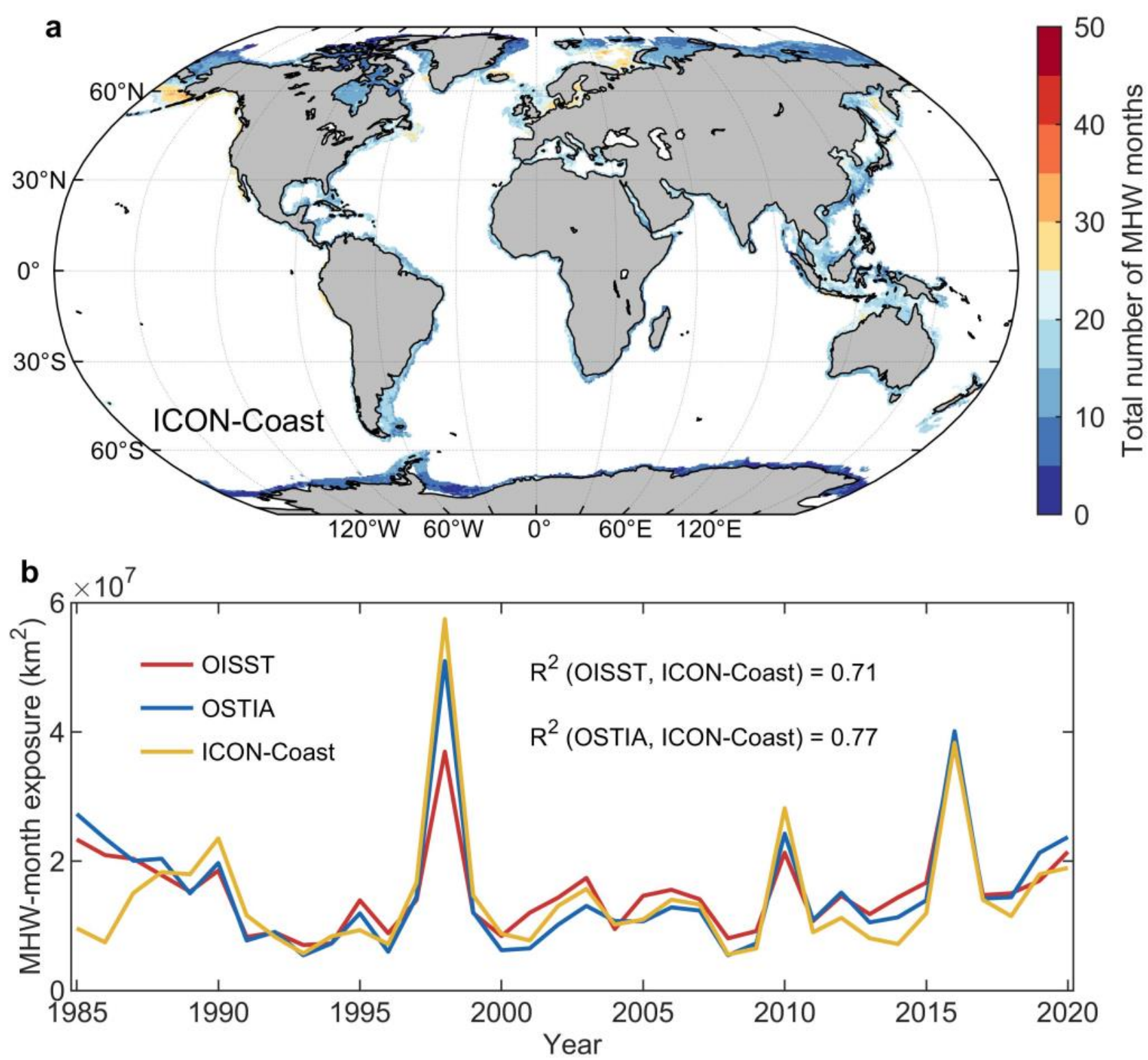

Supplementary Fig. 11 | Model evaluation of MHW-month metrics. (a) *Global distribution of the total number of MHW months (1985–2020) in coastal regions simulated by ICON-Coast.* ***(b)*** *Time series of the integrated MHW-month area from OISST, OSTIA, and ICON-Coast, with R^2 values provided for comparisons between OISST and ICON-Coast, and between OSTIA and ICON-Coast*."

Net effect: it would be interesting to state the net effect not only relative to the global coastal ocean (4.3%), but also relative to the global ocean, to get a sense of the overall impact.

Response: We thank the reviewer for this suggestion. Our analysis is restricted to coastal regions and MHW months, and the anomalies are normalized by a coastal climatology that applies only to MHW-affected coastal areas and MHW months, rather than to all coastal regions and all months over the 36-year period, following the methodology of Mignot et al.[5] and Li et al.[4]. For this reason, the reported enhancement was not directly scaled to the entire global coastal ocean or the global ocean in the first version of the manuscript.

Nevertheless, an order-of-magnitude comparison is informative. Assuming a low annual coastal CO_2 uptake of 0.18 Pg C yr^{-1}[6], the cumulative uptake over 1985–2020 amounts to ~6.5 Pg C, making the reported anomaly of –40.02 Tg C equivalent to ~0.62% of the coastal total uptake. When referenced to a representative global ocean annual uptake of ~2.5 Pg C yr^{-1} (2003–2012)[7], the anomaly corresponds to ~0.05%. Although these fractions appear small at first glance, they represent a systematic and measurable response of coastal carbon cycling to MHW events, which occur in biogeochemically active coastal hotspots.

We have added the content to the main text (Page 5, Lines 125–131): "*The reported percentage anomalies are calculated by normalizing the integrated anomalies against the corresponding climatological sums only for MHW-affected coastal areas and MHW months, following the methodology of Mignot et al.[51] and Li et al.[50]. When referenced to the cumulative CO_2 uptake of the global coastal ocean[11] over 1985–2020 and all months, the reported anomaly of –40.02 Tg C corresponds to ~0.62% of the coastal total uptake, and ~0.05% when referenced to the global ocean[58]. Although this fractional contribution is small, it represents a systematic and measurable response of coastal carbon cycling to MHW events.*"

<u>Specific comments & technical corrections</u>

Ln. 24: “non-thermal DIC”: it could be more appropriate to say “non-thermal reductions in DIC”, since “thermal” does not refer to the DIC itself, but rather to the processes affecting it

Response: Corrected as suggested.

Ln. 47: not sure if “constrained” is the right word here, maybe “perturbed”?

Response: “perturbed” is indeed a more appropriate term here, and the text has been corrected accordingly.

Ln. 54-57: the “coupling” introduced here is not very clear. MHWs are “coupled” to long-term ocean warming everywhere, not just in polar latitudes. The relation between MHWs and sea ice on the other hand is of course a polar phenomenon. The phrasing could be improved

Response: We thank the reviewer for pointing out the inaccuracy in the use of “coupling”. The text has been revised to explicitly separate the global role of long-term ocean warming in shaping MHWs from the polar-specific interactions between MHWs and sea-ice dynamics, and to clarify how these processes differ between the Arctic and the Antarctic coastal ocean.

We have added the following text to revise this statement (Page 3, Lines 57–62): “*<u>Polar shelf seas are particularly sensitive, not only because MHWs are influenced by long-term ocean warming[45,46], as is the case globally[32,35], but also because of their close interaction with sea-ice dynamics in both hemispheres[45-49]. In the Arctic, long-term warming and declining sea ice act to amplify MHW intensity[45,46], whereas in the Antarctic shelf seas, an increase in the occurrence of MHWs promotes sea-ice melt and triggers distinct ecological responses, such as increases in net primary production (NPP)[49].</u>*”

Ln. 68-70: another question (and result of this study) is the net effect of coastal MHWs on carbon uptake, which is more quantitative than the heterogeneity of the response, which is to be expected

Response: We agree that, while heterogeneous regional responses to coastal MHWs are expected, a key contribution of this study is to quantify the integrated net effect on CO_2 uptake. We have

revised the text to emphasize this as a central research question and result, replacing the original sentence with (Page 3, Lines 73–74): "*what is the net effect of MHWs on coastal air–sea CO_2 exchange across regions with heterogeneous responses?*"

Ln. 96: "positive anomalies": mention sign convention

Response: We have clarified the sign convention. The revised sentence now reads (Page 4, Lines 102–105): "*Positive anomalies, indicating enhanced CO_2 outgassing or reduced uptake, prevail in most coastal regions, while negative anomalies, representing enhanced uptake or reduced outgassing, are largely confined to high-latitude, ice-influenced shelf seas (Fig. 1a and Supplementary Fig. 3b).*"

Ln. 112: "segments are ordered by descending class": reword this, e.g. "segments are ordered by descending mean (?) anomaly" or similar.

Response: We have revised the sentence as follows (Page 33, Lines 918–920): "*Classes are ordered by total integrated CO_2 flux anomaly in descending order, with segments within each class further ordered by descending anomaly.*"

Ln. 115-117: it is not immediately clear why the high frequency of MHW months in the Arctic motivates the approach stated in the second part of the sentence

Response: We agree that the original sentence involved a logical jump. By recognizing the particularly high frequency of MHW months in the Arctic, we further realized that the frequency of MHW months is strongly heterogeneous across the global coastal ocean. This heterogeneity means that a simple mean FCO_2 anomaly cannot adequately capture the overall impact of MHWs. We therefore quantify the net effect of MHWs by calculating the integrated FCO_2 anomaly for the global coastal ocean, explicitly accounting for both coastal area and frequency of MHW months.

We also added a description of how area and frequency are accounted for in this calculation. The revised text now reads (Page 5, Lines 112–119): "*Given the strong regional heterogeneity in the frequency of MHW months across the global coastal ocean (Supplementary Fig. 1a,b), particularly the high frequency observed in the Arctic, we quantify the integrated FCO_2 anomaly for the global*

coastal ocean by accounting for both the frequency of MHW months and their spatial coverage. Specifically, for each grid cell and month identified as an MHW month, the FCO_2 anomaly is multiplied by the corresponding grid-cell area and converted to carbon units (Tg). The total integrated anomaly is then obtained by summing across all MHW months and all coastal grid cells, ensuring that both spatial coverage and MHW month occurrence are properly represented."

Ln. 152: "Equation (4)": refer also to the Methods section

Response: We have revised the text to read "*(Equation 4; see Methods)*" (Page 6, Line 163).

Ln. 166: "thermal effect" -> the thermal effect

Response: Corrected as suggested.

Fig. 2: it would be helpful to add a title to panel 2a) (e.g. "Global"), since it is the most important of the small panels but hard to find at first

Response: We have added the title "*Global*" to panel 2a, as suggested.

Fig. 3b, d: indicate the units

Response: The units were km^2. In the revised manuscript, SST has been detrended prior to MHW detection, which substantially reduces the apparent trend in MHW-month frequency. As a result, the previously reported declines in negative integrated sea-ice anomalies are no longer significant. Consequently, the original Figs. 3b,d have been removed to avoid potential misinterpretation.

Ln. 226: "significant declines in negative integrated sea-ice anomalies": the words "negative" and "anomalies" can both be removed without changing the meaning of the sentence (thus making it easier to understand). Replace "anomalies" by "area"

Response: We appreciate the reviewer's suggestion. However, in the original manuscript, the term "negative" cannot be removed and "anomalies" cannot be replaced by "area". We would like to clarify that our analysis is focused on trends in sea-ice anomalies during discrete MHW months,

rather than trends in absolute sea-ice area. Because all anomalies were below zero, retaining "negative" was necessary to indicate their sign. The observed trend arose from the increasing frequency of MHW months rather than sea ice loss during MHW months.

However, in the revised manuscript, SST is detrended prior to MHW detection, which substantially reduces the apparent trend in MHW-month frequency. As a result, the previously reported declines in negative integrated sea-ice anomalies are no longer significant. Consequently, the entire paragraph has been removed to avoid potential misinterpretation and to maintain consistency with the revised MHW month definition.

Ln. 234: "increase in sea ice loss": sea ice area, not loss

Response: Consistent with our earlier response (Ln. 226), the text can be revised as "*increase ($P < 0.05$) in negative sea-ice anomalies*". However, the entire paragraph has been removed now due to the reason we explained in the previous response.

Ln. 235: is the increasing sea ice trend in the Antarctic really "driven by a decline in MHW month frequency"? I would agree that they are consistent, but there are other factors driving sea ice trends, not just MHWs

Response: We agree that "largely driven by" is not accurate, as the decline in MHW month frequency is only one of the factors contributing to the trend. We could replace it with "*largely consistent with*". However, the entire paragraph has been removed (see our previous responses).

Ln. 247: "the overall signs of their effects remain robust": I don't understand what this means, and how it proves the dominance of DIC? The impact of the approximate coefficients should be determined in a more quantitative way

Response: We agree that the previous wording was unclear and did not sufficiently demonstrate the dominance of DIC. In the revised manuscript, we have clarified this statement and provided a more quantitative explanation as follows (Pages 8–9, Lines 222–230) "*Although the contributions of DIC, ALK, and SSS are estimated using approximate sensitivity coefficients, the signs of their effects remain consistent because the sensitivity coefficients in Equation (7) vary within a limited*

range, with γ_{DIC} ranging approximately from 9 to 15 and γ_{ALK} from −9 to −15[18]*. Even when the most extreme plausible coefficients are applied ($\gamma_{DIC} = 9$ and $\gamma_{ALK} = -14$) for all regions, the DIC contribution still dominates. For example, under these extreme coefficients, the average DIC-induced contribution in the Barents and Kara Seas is approximately −10.17 μatm, whereas the corresponding ALK-induced contribution is about −2.18 μatm, which is substantially smaller. These results confirm that DIC acts as the principal regulator of CO_2 uptake anomalies.*"

Ln. 257: "IC and OC": define these terms in the Figure caption

Response: We have defined these terms in the figure caption as "*inorganic carbon (IC) and organic carbon (OC)*" (Page 36, Line 950).

Fig. 4: it should be clearly indicated (e.g. in the first line of the figure caption) whether this is from model output or from observations. On line 246 you indicate that panels a-d are from CMEMS-LSCE, while panels e-h are from ICON (Ln. 268). This is not clear at all from looking at the figure

Response: We thank the reviewer for this suggestion. We have clarified the data sources by adding "*derived from the observation-based CMEMS-LSCE dataset*" and "*based on the ICON-Coast model*".

Ln. 308: "small coastal carbon sink": you previously stated the the coastal carbon sink is disproportionately large?

Response: We thank the reviewer for pointing out this inaccuracy. The coastal carbon sink is indeed considered disproportionately large relative to its area. Our intent was to emphasize that Laruelle et al.[8] provided one of the first quantitative estimates of the coastal ocean CO_2 uptake, which lies toward the lower end of the reported range (0.18–0.45 Pg C yr^{-1})[8]. Specifically, Laruelle et al.[8] estimated a sink of ~0.19 Pg C yr^{-1}, consistent with Roobaert et al.[6] (~0.18 Pg C yr^{-1}).

We have removed the word "small" and revised the text to (Page 11, Lines 295–296) "*which offered one of the first reliable estimates*[4] *of coastal CO_2 uptake at the lower end of reported sink values*".

Ln. 311: “strength” -> strengthen

Response: Corrected.

Ln. 317: “despite uncertainties remain”: check grammar

Response: We have corrected the text to “*Despite remaining uncertainties*” (Page 11, Line 306).

Extended Data: the last few figures are not rendered correctly and lack a caption

Response: Thank you for pointing this out. We have carefully checked these figures and corrected the formatting issue. Following the formatting requirements of Nature Communications, the Extended Data figures have now been moved to Supplementary Information. We believe that the figures are now properly rendered and include the appropriate captions.

Eq. 4: it looks like some of the “ice” variables are rendered with a different letter “i” (someone didn’t “dot their i’s” as they say...)

Response: Thank you for pointing this out. The issue with the letter “i” in “ice” when a bar is applied has been resolved; the dot now appears correctly in all cases.

References for Responses to Reviewer #1

1. Zheng, J. et al. Phytoplankton response to the record-breaking marine heatwave in the summer of 2020 in the South China Sea. *J. Geophys. Res. Oceans* **129**, e2024JC021275 (2024).
2. Ford, D. J., Nair, S. P., Oglethorpe, K. & Shutler, J. D. Regionally different marine heatwave ocean carbon sink responses are consistent with carbonate understanding. *Environmental Research Communications*, 111009 (2025).
3. Edwing, K. et al. Impact of marine heatwaves on air-sea CO_2 flux along the US East Coast. *Geophys. Res. Lett.* **51**, e2023GL105363 (2024).
4. Li, C., Burger, F. A., Raible, C. C. & Frölicher, T. L. Observed regional impacts of marine heatwaves on sea-air CO_2 exchange. *Geophys. Res. Lett.* **51**, e2024GL110379 (2024).

5. Mignot, A. et al. Decrease in air-sea CO_2 fluxes caused by persistent marine heatwaves. *Nat. Commun.* **13**, 4300 (2022).
6. Roobaert, A., Regnier, P., Landschützer, P. & Laruelle, G. G. A novel sea surface pCO_2-product for the global coastal ocean resolving trends over 1982–2020. *Earth Syst. Sci. Data* **16**, 421–441 (2024).
7. Le Quéré, C. et al. Global carbon budget 2013. *Earth Syst. Sci. Data* **6**, 235–263 (2014).
8. Laruelle, G. G., Lauerwald, R., Pfeil, B. & Regnier, P. Regionalized global budget of the CO_2 exchange at the air-water interface in continental shelf seas. *Glob. Biogeochem. Cycles* **28**, 1199–1214 (2014).

<u>Reviewer #2 (Remarks to the Author):</u>

Dear Authors,

your paper is interesting, and mostly talks about trends in some pCO2 and air-sea CO2-flux products (not necessarily marine heat waves, the way they are described).

Response: We sincerely thank the reviewer for the constructive comments and suggestions. In the revised manuscript, SST has been detrended prior to MHW detection to avoid the influence of long-term warming trends. The manuscript has been revised accordingly, and detailed point-by-point responses are provided below, with the major comments appropriately organized into separate points.

However, I have a few **<u>major comments</u>**.

1: how heat waves are defined here seem to me to unfortunately mix up different things (if I am not mistaken). It is applied to monthly averages and there are two main criteria used. In regions where there are large positive trends, the first one will select only months in the warm (recent) part of the time series (that's very clear from the supp mat figures) (a month kept if it have more than 15 days of heat wave day, which is also a strange criteria). The second one tries to minimize that, but only considering months that stand out 'positive' in the detrended time series. At the end, I dont understand what we are looking at, and I really dont see the added value of having done the large effort of defining a heat wave, etc... from daily SST field. I would like to get convinced that we would not get basically the same results by simply looking at monthly fields and anomalies, and what the added complexity brings us. I personnally feel that the heat wave should be defined in detrended time series. One could at the end look at months with similar criteria which would pass the criterium of the 15 days ... ANd there is anyway a caveat which is that the reconstructed pCO2 products are monthly (and rightly so, as the construction method is eally geared to relatively low frequencies). How will you be confident that this is a quasi linear system relative to heat wave conditions?

Response: We sincerely thank the reviewer for this important and constructive comment. After careful consideration, we have revised our methodology and detrended SST prior to MHW detection.

In the first version of the manuscript, we intentionally retained the SST trend to preserve the increasing frequency of MHWs. To maintain consistency with the subsequent decomposition of FCO_2 anomalies (which was relative to trend-preserving climatology), we introduced a secondary criterion for defining MHW months to exclude cases in which the monthly mean SST was lower than the climatology calculated from detrended SST in some regions. We now recognize that this two-criterion approach introduced an inconsistency between the MHW month definition and the CO_2 flux anomaly framework. We have therefore revised our methodology by detrending SST prior to MHW detection.

As discussed by Amaya et al.[1], MHWs can be defined relative to either a fixed historical baseline or a shifting baseline that accounts for long-term ocean warming. The fixed-baseline approach evaluates extremes relative to past climatology, whereas the shifting-baseline approach removes the warming trend to isolate short-term variability. Because these definitions yield different estimates of MHW characteristics and trends, the choice of baseline should be aligned with the research objective. In our study, the primary goal is to investigate the contemporaneous anomalous impact of MHWs on air–sea CO_2 flux in the global coastal ocean, for which a shifting-baseline framework is more appropriate to isolate event-scale variability from the background warming trend.

Zhang et al.[2] (following Amaya et al.[1]) detected MHWs using a 31-year sliding climatological window to represent evolving background conditions and thereby remove the long-term warming effect. However, our study period spans 1985–2020, making a full 31-year sliding climatology impractical for all years. Therefore, we detrended the SST time series prior to MHW detection to remove the long-term warming influence while retaining short-term variability.

In the revised manuscript, we detrended SST before MHW detection as suggested by the reviewer, and we now define MHW months using a single criterion: a month is classified as an MHW month if it contains at least 15 MHW days. In addition, we have revised the climatological reference period from 1985–2014 to 1985–2020. The sentences now read as follows: (Page 13, Lines 358–359) "*SST data during 1982–2020 were detrended prior to MHW detection to remove the long-term influence, and MHWs were subsequently identified over 1985–2020.*", (Pages 14–15, Lines 384–391)"*To match the monthly resolution of the $pCO_{2,sea}$ and FCO_2 data, we defined MHW months by aggregating daily MHW metrics within each month, following the approach of Edwing*

et al.[28]*. For each grid cell, the number of MHW days was calculated for each month during 1985–2020. A month was classified as an MHW month only if it contained at least 15 MHW days, ensuring that the thermal perturbations were strong enough to induce ecological impacts and influence monthly* CO_2 *fluxes*[28,99]*. This criterion ensured that MHW months represented statistically significant thermal anomalies (Supplementary Fig. 8e), while also maintaining a sufficient sample size for robust assessment of carbon cycle responses.*”

The time series of MHW months is shown below (Supplementary Fig. 1c). The increasing trends disappear for both SST datasets.

“

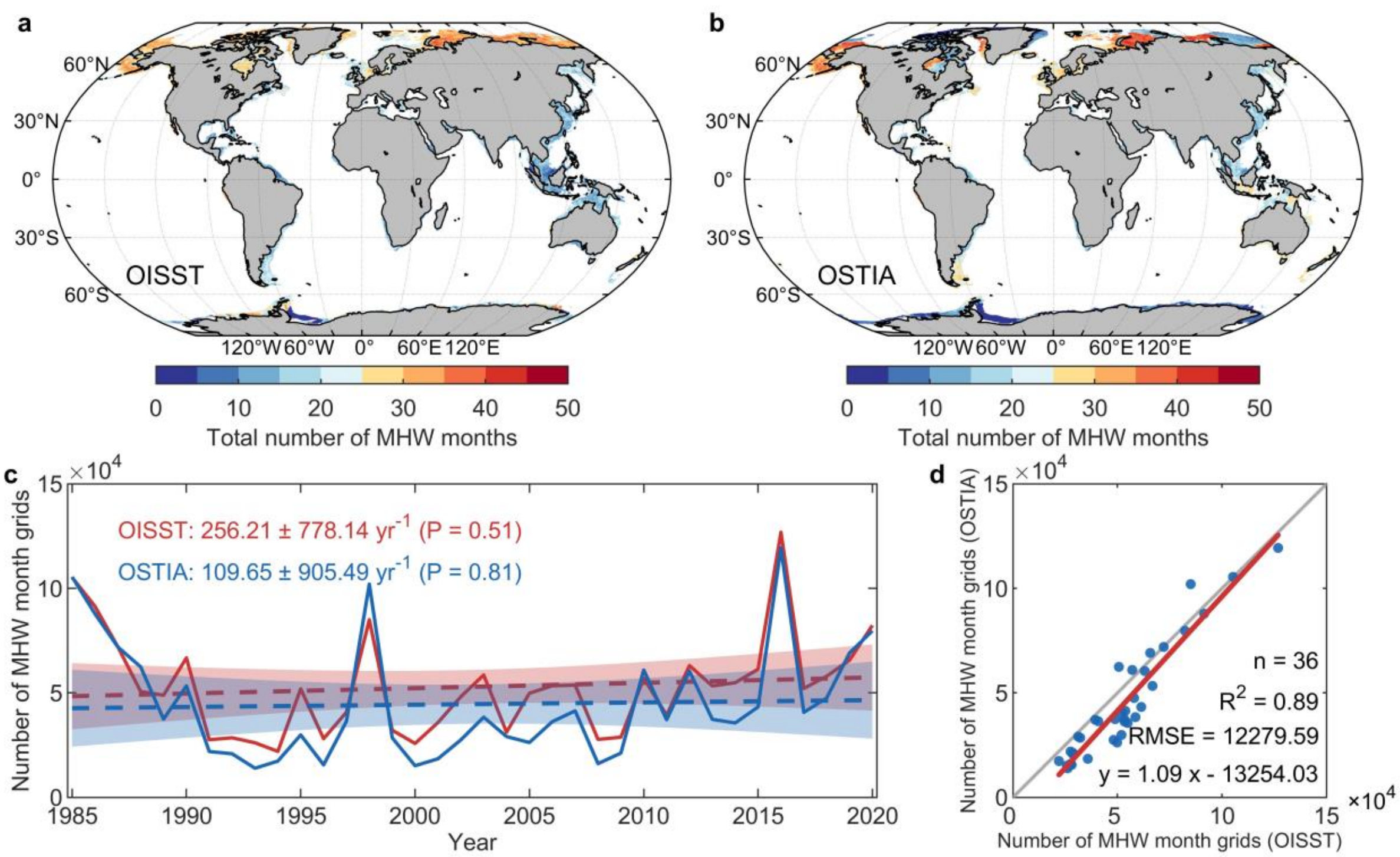

Supplementary Fig. 1 | Consistency in MHW month detection between OISST and OSTIA datasets (1985–2020). *The global distribution of the total number of MHW months in coastal regions by the OISST dataset (**a**) and the OSTIA dataset (**b**). (**c**) Time series of the annual total number of grid cells identified as MHW months (OISST data in red and OSTIA data in blue). Dashed lines indicate linear trends, and shaded bands represent 95% confidence intervals. Trend slopes and corresponding P values are provided in the panel. (**d**) Scatterplot comparison of monthly grid cell counts between the two datasets. Data volume,* R^2*, RMSE, and regression lines are provided.*”

All analyses have been recalculated accordingly. While detrending SST prior to MHW detection changes the time series figures and largely removes long-term trends (such as Supplementary Fig. 6), the main conclusions remain robust, with only moderate quantitative adjustments, such as the change in the integrated CO_2 flux anomaly from 4.3 ± 0.2% to 11.0 ± 1.6%.

“

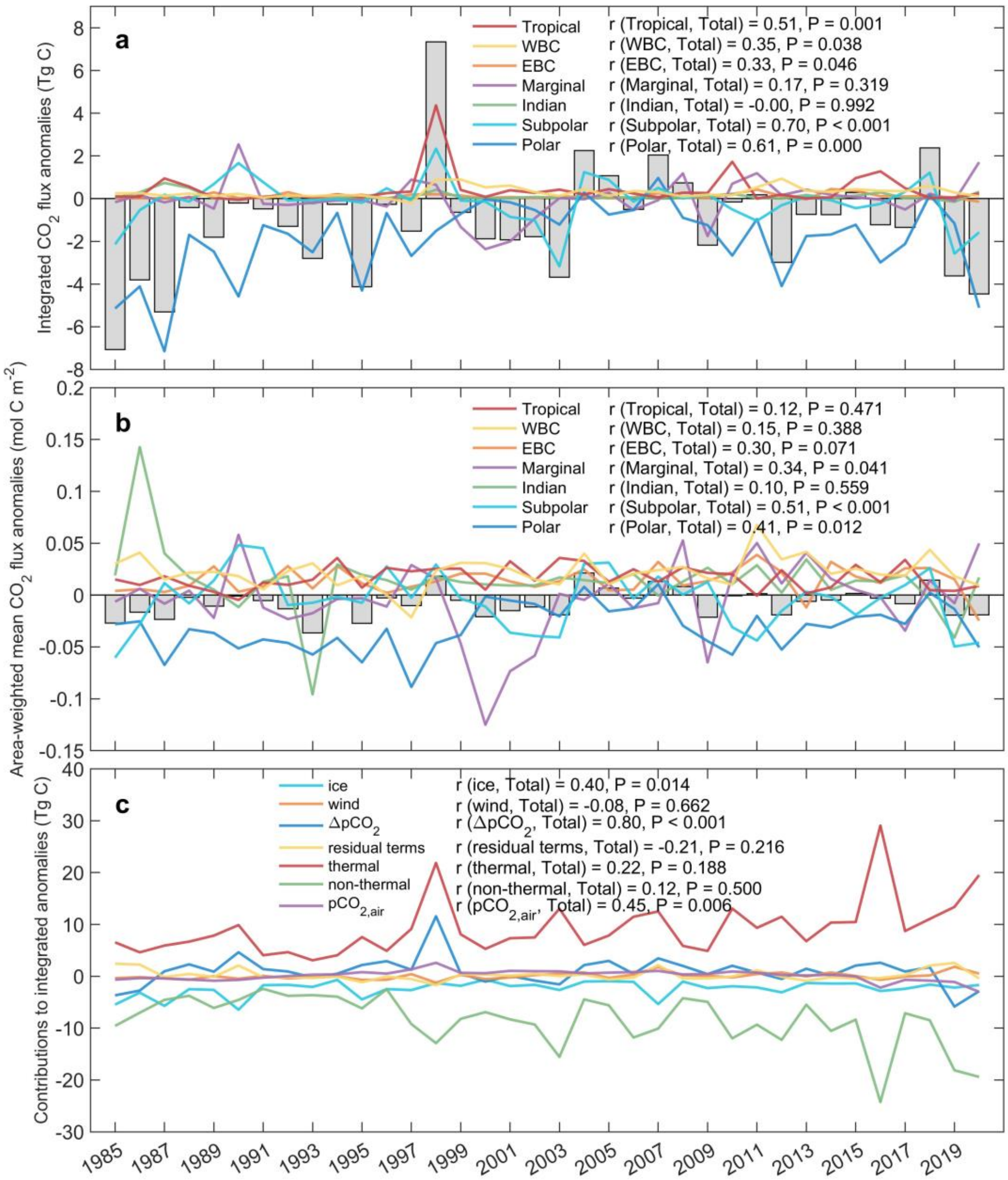

Supplementary Fig. 6 | Temporal dominance of regions and factors contributing to integrated FCO_2 anomalies. (a) *Time series of integrated FCO_2 anomalies for the global coastal ocean (bars) and across seven distinct regional classes. Correlation coefficients and P values are provided.* ***(b)***

*same as (**a**), but for area-weighted mean FCO_2 anomalies. (**c**) Time series of integrated FCO_2 anomalies driven by seven factors, with correlation coefficients and P values indicated. All data shown are derived from the observation-based ULB–SOM–FFN–coastalv2 dataset*[1]."

The choice of defining MHW months is a compromise solution, based on two considerations. First, the reconstructed FCO_2 products are provided at monthly resolution, and we need to link them with MHWs. We use a threshold of 15 days to define MHW months because, as noted by Edwing et al.[3], extreme temperatures that persist for at least half of a month are expected to be reflected in pCO_2 and CO_2 flux data. The threshold of 15 days was also tested through eleven sensitivity runs to ensure robustness. Second, the net effects of MHWs on FCO_2 or other biogeochemical metrics are not necessarily confined to the days with anomalously high SST; they may persist beyond the event itself or reach maximum effect after MHWs. Therefore, using MHW months provides a more realistic, general, and inclusive representation of the carbon flux response than limiting the analysis to days with high SST only.

We also considered defining MHW months based on monthly mean high SST, as suggested by the reviewer, following the methodology of Li et al.[4]. In this approach, MHW months are identified when linearly detrended monthly-mean SST exceeds the local seasonally varying 90th percentile. However, the concept of MHW differs from that of high SST. MHWs are defined not only by intensity but also by duration, whereas monthly high SST mainly reflects background warming effects. MHWs represent episodic thermal disturbances that can trigger nonlinear and potentially amplified carbon flux anomalies. Moreover, our study focuses on regional heterogeneity in MHW occurrence across the global ocean. If we were to use monthly mean high SST as in Li et al.[4], the resulting number of "MHW months" per grid cell would be uniform across regions, failing to capture this spatial heterogeneity.

Finally, we acknowledge that reconstructed pCO_2 products are monthly and primarily designed to capture seasonal to interannual variability. Nevertheless, in our study, the thermal anomalies associated with MHW months persist over a substantial fraction of the month, influencing monthly mean physical and biogeochemical conditions. Since these products are based on in-situ observations, they retain, to some extent, the CO_2 exchange anomalies induced by MHW months.

Regarding the reviewer's concern about quasi-linearity, we evaluate FCO_2 anomalies during MHW months by testing whether they are significantly different from zero. The absence of

significant anomalies in some regions does not imply a lack of response but rather reflects the coexistence of competing mechanisms. When analysing thermal and non-thermal contributions separately, we find that in most regions both contributions are significantly different from zero (Supplementary Fig. 8), indicating that the tug-of-war between thermal and non-thermal factors is widespread. Importantly, the responses of both thermal and non-thermal factors during MHW months scale approximately linearly with the occurrence of MHW conditions, which is a key point of our study. This approximate linearity allows us to reveal how physical and biological processes in coastal regions respond to MHWs.

2: the analysis is based on a heterogeneous set of products which are poorly described in the paper, and it is a bit hard to get in particular at what are there respective advantages and issues based on what is written in the Supp mat. Also, among those five products, four are explicitely described as extrapolating offshelf pCO2 (fluxes) to the coastal and shelf regions (which is not always the case, actually in those, such as for the Barents Sea).

Response: We thank the reviewer for this helpful comment. We agree that the description of the different data products in the previous version was too short and did not clearly highlight their respective methodologies, strengths, and limitations.

We also appreciate the reviewer's point regarding the wording suggesting that these products extrapolate offshore pCO_2 or flux estimates into coastal regions. We agree that this wording was inaccurate. In fact, the datasets incorporate coastal observations and dedicated reconstruction approaches to estimate pCO_2 and air–sea CO_2 fluxes across both open-ocean and coastal domains. We have therefore removed the words "merely extend open-ocean values into nearshore areas" to avoid this misunderstanding.

We have therefore revised the Methods section to provide a clearer description of each dataset, as shown below (Pages 15–16, Lines 402–432): "*In this study, we employed five observation-based products (Supplementary Table 1), all aimed at providing observation-constrained estimates of surface ocean pCO_2 and air–sea CO_2 flux, differing in methodology, spatial resolution, and reconstructed variables. ULB–SOM–FFN–coastalv2[11] combines Self-Organizing Maps (SOM) network with Feed-Forward Network (FFN), specifically targeting the coastal ocean at 0.25° × 0.25° resolution. It extends the work of Laruelle et al.[15] by including a broader set of predictors*

and enhanced validation. Its high resolution captures fine-scale variability in coastal air–sea CO_2 exchange[11,15]*, though uncertainties remain elevated in regions with strong spatiotemporal variability and sparse observations.*

The other four datasets, mainly developed for global ocean applications, were used to complement and cross-validate the coastal regions. Combining these products into an ensemble framework enhances confidence in the robustness of coastal flux anomalies, particularly in data-sparse high-latitude shelf seas. CMEMS-LSCE-FFNN[53] *employs an ensemble of feed-forward neural networks trained on SOCAT observations and environmental predictors, providing global reconstructions at 0.25° resolution, although uncertainties remain higher in coastal regions and data-sparse regions such as the Southern Ocean and upwelling systems. OceanSODA-ETHZ*[54] *employs the Geospatial Random Cluster Ensemble Regression (GRaCER) framework to reconstruct multiple carbonate variables (e.g., pCO_2, DIC, ALK, and pH) at 1° resolution. By incorporating coastal observations and multiple clusters, it improves the representation of coastal variability and extends the coverage into the coastal margin, providing a useful basis for examining coastal carbon flux variability, including under MHW conditions, although uncertainties remain high due to strong spatiotemporal variability and representation errors.*

SeaFlux[55] *differs fundamentally from the other products in that it does not generate a new pCO_2 reconstruction but instead provides a harmonized framework for calculating air–sea CO_2 fluxes from multiple existing pCO_2 products. By standardizing gas-exchange parameterizations, wind products, and flux calculations, SeaFlux enables more consistent comparisons among different flux estimates while reducing methodological discrepancies, although the resulting fluxes remain dependent on the quality and coverage of the input pCO_2 datasets. Finally, CMEMS-LSCE*[66] *provides a 0.25° monthly reconstruction of pCO_2, ALK, DIC, pH, and carbonate saturation states with internally consistent multi-variable estimates suitable for assessing coastal carbon fluxes. It also improves nearshore coverage and representation of the coastal–open ocean continuum, although uncertainties remain high in highly dynamic coastal environments.*"

For that reason, they are considered 'inferior' to the fifth product (by Roobaert et al., 2024), but are nonetheless included, which in some ways is a bit contradictory. It is also not clear how the information from the different products are merged (or is it only in Supp Mat).

Response: While the other three FCO_2 datasets are considered "inferior" to the ULB–SOM–FFN–coastalv2 dataset for coastal applications, they were included to cross-validate the enhanced integrated CO_2 flux anomaly for the global coastal ocean (Supplementary Fig. 4), which is consistently negative across all products and therefore robust. The ULB–SOM–FFN–coastalv2 dataset, specifically designed for coastal regions, remains the primary dataset for the main analyses, including the integrated flux calculations over the 45 segments and the decomposition.

To improve clarity, all figure captions have been revised to explicitly indicate the dataset used, for example, "*All data shown are derived from the observation-based ULB–SOM–FFN–coastalv2 dataset*" or "*derived from the observation-based CMEMS-LSCE dataset*", ensuring that readers can clearly identify the source of each result.

Furthermore, Roobaert et al explicitely mentions the issue of sampling and potential biases and errors in regions with partial or seasonal ice cover (ice rate change been a key parameter in the training and construction of the product) (see the comments on regions 1, 5 and 7). In many ways, these are the regions where the largest impact of the trends (I'll stop calling that heat waves!) is found.

Response: We thank the reviewer for pointing out the potential sampling limitations and uncertainties in regions with partial or seasonal sea-ice cover, as discussed in Roobaert et al.[5]. To address this concern, we have detrended the SST before MHW detection and added a quantitative uncertainty assessment that propagates the RMSE between reconstructed pCO_2 and SOCAT observations into the uncertainty of the derived air–sea CO_2 flux anomalies. We also considered the integrated coastal flux uncertainty reported by Roobaert et al.[5] (0.01 Pg C yr^{-1}), which accounts for uncertainties related to ΔpCO_2, gas transfer velocity, wind speed, and sea-ice cover. These additional analyses allow us to quantify the uncertainty of the integrated FCO_2 anomaly during MHW months and evaluate its robustness.

The corresponding methodology and results have now been added to the Supplementary Note and Discussion sections below (Pages 11–12, Lines 310–331):

"*Roobaert et al.[11] highlighted that reconstructed coastal pCO_2 products may contain biases, particularly in regions with sparse observations or seasonal sea-ice cover. In three of the ten*

biogeochemical provinces (P1, P5, and P7), the root mean square error (RMSE) exceeds 35 μatm between reconstructed pCO_2 and SOCAT observations[11]. To evaluate the potential influence of such biases on our results, we propagated the upper-bound RMSE into the uncertainty of the air–sea CO_2 flux anomaly (Supplementary Note 1), thereby deriving a conservative estimate. When aggregated within each of the 45 coastal segments, the resulting uncertainty is relatively small compared with the estimated FCO_2 anomaly (Supplementary Fig. 10). When further aggregated across all coastal regions, the combined uncertainty remains limited (0.91 Tg C), largely because the large number of contributing grid cells reduces the overall uncertainty through statistical aggregation. In addition to reconstruction errors, the coastal flux product itself includes uncertainties associated with ΔpCO_2, gas transfer velocity, wind speed, and sea-ice cover. Roobaert et al.[11] estimated that the combined effect of these factors results in an uncertainty of approximately 0.01 Pg C yr^{-1} for the integrated coastal air–sea CO_2 flux. Although MHW months occur more frequently in recent decades with improved observational coverage, where uncertainties are lower than this long-term mean value[11], we adopt a conservative estimate by assuming temporal and spatial independence of uncertainties and scaling the variance according to the fraction of the coastal domain affected by MHW months (Supplementary Note 1). Under this assumption, the upper-bound uncertainty of 0.01 Pg C yr^{-1} corresponds to an uncertainty of 17.26 Tg C for the integrated CO_2 flux anomaly during MHW months. The resulting signal-to-noise ratio (SNR) is 2.38, suggesting that the enhanced coastal CO_2 uptake during MHW months remains relatively robust despite the reconstruction uncertainties."

"

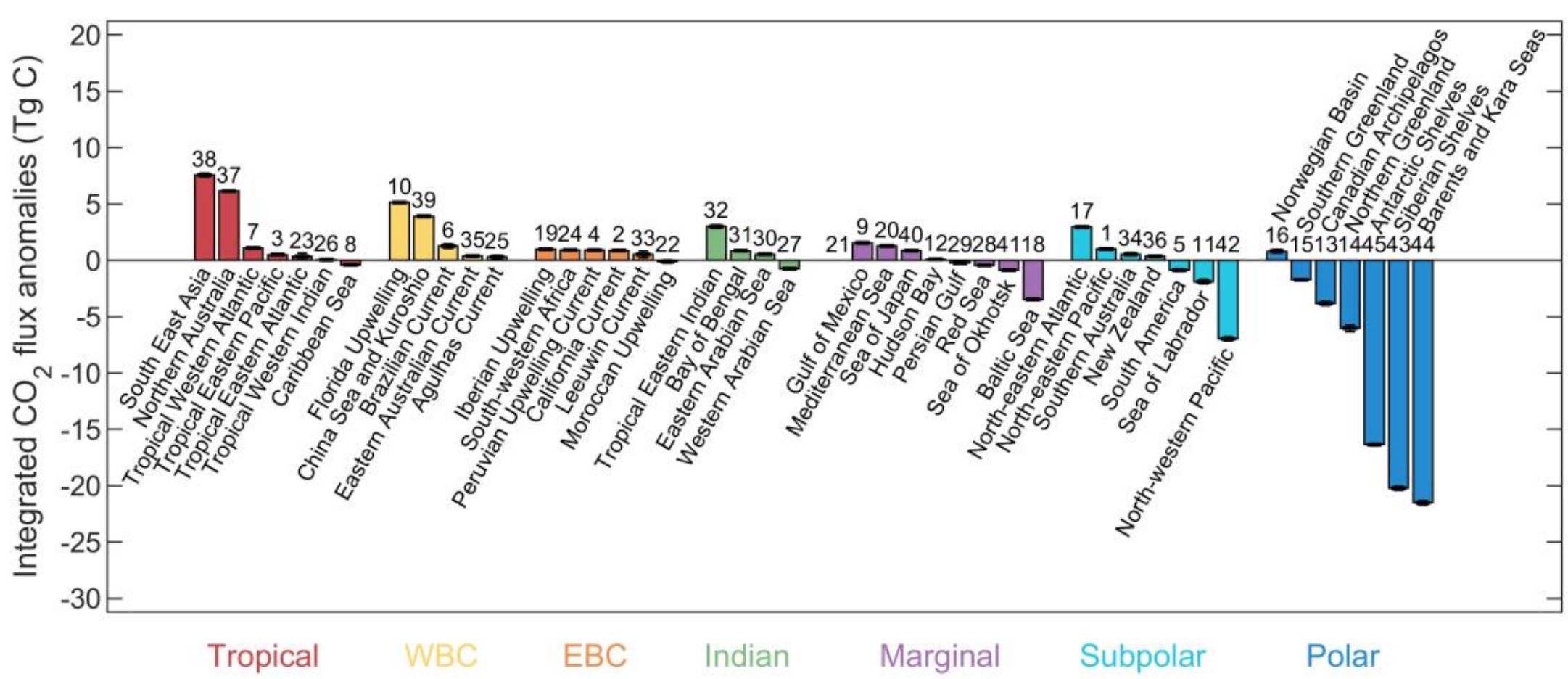

Supplementary Fig. 10 | Uncertainty of integrated coastal FCO_2 anomalies associated with reconstructed coastal pCO_2 RMSE. *Bars represent the integrated CO_2 flux anomalies calculated using a 15-day threshold to define MHW months. Error bars represent uncertainties derived from the RMSE between reconstructed pCO_2 and SOCAT observations[1] for each of the 45 coastal segments[2]. Classes are ordered by total integrated CO_2 flux anomaly in descending order, with segments within each class further ordered by descending anomaly. Segment names and their serial numbers are shown.*"

(Pages 21–22, Lines 132–177 in Supplementary Information) "***Supplementary Note 1 | Uncertainty estimation of integrated FCO_2 anomaly during MHW months.***

Following Roobaert et al.[1], two complementary sources of uncertainty were considered: (1) the reconstruction error of pCO_2, quantified by the RMSE between reconstructed pCO_2 and SOCAT observations, and (2) the uncertainty of the FCO_2 product, which accounts for uncertainties associated with ΔpCO_2, gas transfer velocity, wind speed, and sea-ice cover. These two components were used to estimate the uncertainty of the integrated FCO_2 anomaly during MHW months.

In three of the ten biogeochemical provinces (P1, P5, and P7), the RMSE exceeds 35 μatm[1]. Because the air–sea CO_2 flux is proportional to the air–sea pCO_2 difference (ΔpCO_2), uncertainties in pCO_2 translate directly into uncertainties in the estimated flux. The associated flux uncertainty can therefore be expressed as

$$\sigma_{FCO_2} = k \cdot K_0 \cdot (1 - ice) \cdot RMSE \qquad (1)$$

Because the FCO_2 anomaly is defined as the difference between the instantaneous flux and its climatological value, uncertainties from both terms contribute to the anomaly uncertainty. Because both the instantaneous flux and its climatological value are derived from the same reconstructed product, they are expected to have comparable uncertainties. The resulting anomaly uncertainty can be approximated as

$$\sigma_{FCO_2,anom} = \sqrt{2} \cdot \sigma_{FCO_2} \qquad (2)$$

For each grid cell and each MHW month, the anomaly uncertainty was converted to carbon units by multiplying by the grid-cell area (A_i) and converting from mol C to mass units. The total

uncertainty associated with the integrated FCO_2 anomaly during MHW months was then obtained by combining grid-cell uncertainties under the assumption that errors are spatially independent:

$$\sigma_{total} = \sqrt{\sum_i (\sigma_{FCO_2,anom,i} \cdot A_i)^2} \quad (3)$$

In addition to reconstruction errors, we further considered the uncertainty of the integrated coastal air–sea CO_2 flux reported by Roobaert et al.[1], which is estimated to be 0.01 Pg C yr^{-1} for the global coastal domain. To apply this annual uncertainty to monthly estimates, we assumed that uncertainties are temporally independent between months, with variances additive over time. Under this assumption, the variance of the monthly integrated flux can be expressed as

$$Var_{month} = \frac{(0.01)^2}{12} \quad (4)$$

Because MHW months occur only over a fraction of the coastal domain in each month, the uncertainty was further scaled according to the proportion of coastal area affected by MHW months. Let A_{MHW} denote the total area of grid cells experiencing MHW months during the 36-year study period, and A_{tot} the total coastal area. The variance associated with the integrated FCO_2 anomaly is therefore

$$Var_{MHW} = Var_{month} \times \frac{A_{MHW}}{A_{tot}} \quad (5)$$

Since the FCO_2 anomaly is defined as the difference between instantaneous flux and its climatological value, the variance of the anomaly becomes

$$Var_{anom} = 2 \times Var_{MHW} \quad (6)$$

The corresponding uncertainty of the integrated FCO_2 anomaly is therefore

$$\sigma_{anom} = \sqrt{2 \times Var_{MHW}} \quad (7)$$

Finally, the robustness of the estimated FCO_2 anomaly during MHW months was evaluated using the signal-to-noise ratio (SNR):

$$SNR = \frac{|F_{anom}|}{\sigma_{anom}} \quad (8)$$

where F_{anom} is the integrated FCO_2 anomaly during MHW months and σ_{anom} is the corresponding uncertainty."

3: part of the reason why regions with partial ice cover are more uncertain (OK: except parts of the Arctic, where the change in time is major...) is that there are not that many data in many of those places and how one defines the sea ice mask has strong implications on the reconstructions... Notice also that SST products have also rather large uncertainties in some of these regions...

Response: We agree that ice-covered regions are characterized by sparse observations and sensitivity to sea-ice datasets, which can affect reconstruction accuracy. Nevertheless, previous studies[5-7] have made important efforts to quantify air–sea CO_2 fluxes in these regions to reduce gaps in global coastal carbon budgets. Roobaert et al.[5] quantified the associated uncertainties associated with pCO_2 reconstruction, as well as those arising from different data products of ΔpCO_2, gas transfer velocity, wind speed, and sea-ice cover.

In this study, we explicitly accounted for these uncertainties, including sea-ice cover, as detailed in our previous response. By propagating reconstruction errors and incorporating the reported flux uncertainty, we quantitatively assessed their impact on the integrated FCO_2 anomalies (see Discussion part). Despite the higher uncertainty in ice-affected regions, the aggregated signal remains robust, supporting our main conclusion of enhanced coastal CO_2 uptake during MHW months and the dominant role of high-latitude regions.

We also note that SST products are more uncertain in ice-covered regions. To address this, we repeated the analysis using two independent SST datasets (OISST and OSTIA). Both yield consistent MHW-month time series (Supplementary Fig. 1), and the corresponding integrated FCO_2 anomalies show similar enhanced CO_2 uptake signals (Supplementary Fig. 4). This consistency across independent datasets further supports the robustness of our results.

Now, I dont have particular objections to what is stated on explaining the changes and it is all logical with the way the products are constructed, and to current knowledge, but that's related to a good extent (for the Arctic for example) to the low frequency pluri-annual variability/trends.

I would suggest that the paper with revisited. If the emphasis is on heat waves (even if combined in monthly categories), then do it in a coherent way , which excludes the trends in SST during the selection, and compare those months with non-heat wave month for the same part of the time series.

There might be interesting trends in that too! Or, adopt a low frequency approach directly based on the monthly means.

Response: We have detrended the SST prior to the detection of MHWs as suggested.

We note that the FCO_2 anomaly is calculated based on a trend-preserving climatology. Therefore, FCO_2 anomalies during non-MHW months generally exhibit an opposite tendency compared to those during MHW months.

The integrated FCO_2 anomaly during MHW months is closely linked to the identification of MHW months. Whether SST is detrended or not, the resulting FCO_2 anomalies remain meaningful. Without detrending, they reflect extremes relative to the historical climatology, although this approach tends to preferentially select months from the warm (more recent) part of the time series. In contrast, detrending SST better isolates short-term variability. In both approaches, months with relatively high SST can be identified, that is why the signal of enhanced CO_2 uptake is consistent across the two methodologies.

On balance, we consider the detrended approach to be more appropriate. The updated results based on MHW months derived from detrended SST show a larger enhancement in integrated global coastal CO_2 uptake during MHW months, with the percentage increasing from 4.3 ± 0.2% to 11.0 ± 1.6%.

Minor comments:

l. 311: to further strengthen…

Response: Corrected as suggested.

l. 318: Despite remaining uncertainties

Response: Corrected as suggested.

References for Responses to Reviewer #2

1. Amaya, D. J. et al. Marine heatwaves need clear definitions so coastal communities can adapt. *Nature* **616**, 29–32 (2023).

2. Zhang, M. et al. Spatiotemporal variability of air-sea CO_2 fluxes in response to El Nino-related marine heatwaves in the tropical Pacific Ocean. *Mar. Environ. Res.* **204**, 106949 (2025).
3. Edwing, K. et al. Impact of marine heatwaves on air-sea CO_2 flux along the US East Coast. *Geophys. Res. Lett.* **51**, e2023GL105363 (2024).
4. Li, C., Burger, F. A., Raible, C. C. & Frölicher, T. L. Observed regional impacts of marine heatwaves on sea-air CO_2 exchange. *Geophys. Res. Lett.* **51**, e2024GL110379 (2024).
5. Roobaert, A., Regnier, P., Landschützer, P. & Laruelle, G. G. A novel sea surface pCO_2-product for the global coastal ocean resolving trends over 1982–2020. *Earth Syst. Sci. Data* **16**, 421–441 (2024).
6. Laruelle, G. G. et al. Global high-resolution monthly pCO_2 climatology for the coastal ocean derived from neural network interpolation. *Biogeosciences* **14**, 4545–4561 (2017).
7. Dai, M. et al. Carbon fluxes in the coastal ocean: synthesis, boundary processes, and future trends. *Annu. Rev. Earth Planet. Sci.* **50**, 593–626 (2022).

<u>Reviewer #3 (Remarks to the Author):</u>

Review of Nature Communications ID NCOMMS-25-92847-T: 'Polar and subpolar shelf seas dominate enhanced coastal CO_2 uptake during marine heatwaves' by Zhang et al.

Table of Contents

Summary..1

Issue 1: MHW definition...3

Issue 2: Model evaluation..4

Minor issues..4

References...9

<u>Summary</u>

Zhang et al. investigate today's global coastal ocean carbon cycle during marine heat waves (MHWs) based on pCO_2 data products. In addition, a global ocean biogeochemistry model is utilized to identify its driving mechanisms.

They find that the observed monthly coastal ocean air-sea CO_2 flux (FCO_2) anomaly – i.e. FCO_2 during MHW months minus the 1985 to 2014 climatology – is not significantly different from zero in many coastal regions (Extended Data Fig. 2d). Individual drivers of this FCO_2 anomaly, however, are largely significantly different from zero (Extended Data Fig. 6), and the authors conclude (L101): 'The absence of significant anomalies in many regions does not indicate a lack of response but rather the coexistence competing mechanisms.'.

Keeping the often insignificant FCO_2 anomaly in mind, Zhang et al. show that during MHW months, the global coastal ocean CO_2 uptake is strengthened by ~4%, depending on the data product (Extended Data Fig. 3). The authors stress that this coastal signal contrasts open ocean FCO_2 anomalies during MHW conditions, as Li et al., 2024 (ref 50) reported a reduced global open ocean CO_2 uptake by 8%.

The largest contribution to this global coastal FCO2 anomaly originates from the large Arctic and Antarctic shelves (Figure 3 of Dai et al., 2022; ref 7), as most non-polar coastal regions show the opposite: reduced uptake/enhanced outgassing during MHW months (Fig. 1b).

In these non-polar coastal regions, the FCO2 anomaly is mainly set by thermal drivers of pCO2 changes (Fig. 2), similarly shown for the open ocean by Li et al., 2024 (their Figure 3a; ref 50). For polar coastal regions, Zhang et al. here identify a large compensation of thermal and non-thermal drivers of pCO2 changes (Fig. 2), the latter being, together with retreating sea ice, the most important driver of FCO2 anomalies (Fig. 2, Extended Data Fig. 5a, f).

Finally, a modeled coastal DIC budget of the upper 50m reveals that the coastal DIC decrease during MHW months, i.e. the important non-thermal driver of pCO2 changes, is largely driven by enhanced biological carbon fixation (Fig. 4), probably due to warmer seawater and increased light availability due to sea ice loss.

This work focuses on the coastal ocean, thereby complementing a similar analysis for the open ocean by Li et al., 2024 (ref 50). I thus think that the manuscript is a meaningful contribution to understand the global carbon cycle in a changing world, particularly during extreme events. I think the manuscript is interesting, well written, and that there is nothing wrong. I particularly like that the significance of the FCO2 anomaly and its drivers is documented (Extended Data Fig. 2, 5, and 6). Most of my comments are rather minor.

At the same time I think the authors could have taken the chance and provide the shown analyses for both the open and coastal oceans as they had all necessary data at hand anyway. For example, zonal mean plots of the FCO2 anomaly per unit area during MHW months, similarly as Figure 4 of Roobaert et al., 2019 (ref 16) would have been a great contribution. Similarly, I do appreciate the analysis of the modeled DIC budget. However, I am not sure if the FCO2 anomaly and its thermal and non-thermal drivers are sufficiently represented in the model, as to me Fig. 2a versus Supplementary Fig. 2 as well as Extended Data Fig. 5e, f versus Supplementary Fig. 3 are somewhat difficult to compare by eye. For this, I would like to see e.g. zonal mean plots (or similar

as Fig. 2a) showing both data product and model results at once. Finally, I find the discussion rather superficial and thus not very helpful, perhaps due to word limitations.

Response: We thank the reviewer for this constructive suggestion. Following this recommendation, we additionally provide zonal mean distributions of *F*CO_2 anomalies during MHW months for both the open ocean and coastal ocean (Supplementary Fig. 3). This analysis provides a more direct comparison of MHW responses between the open and coastal ocean.

“

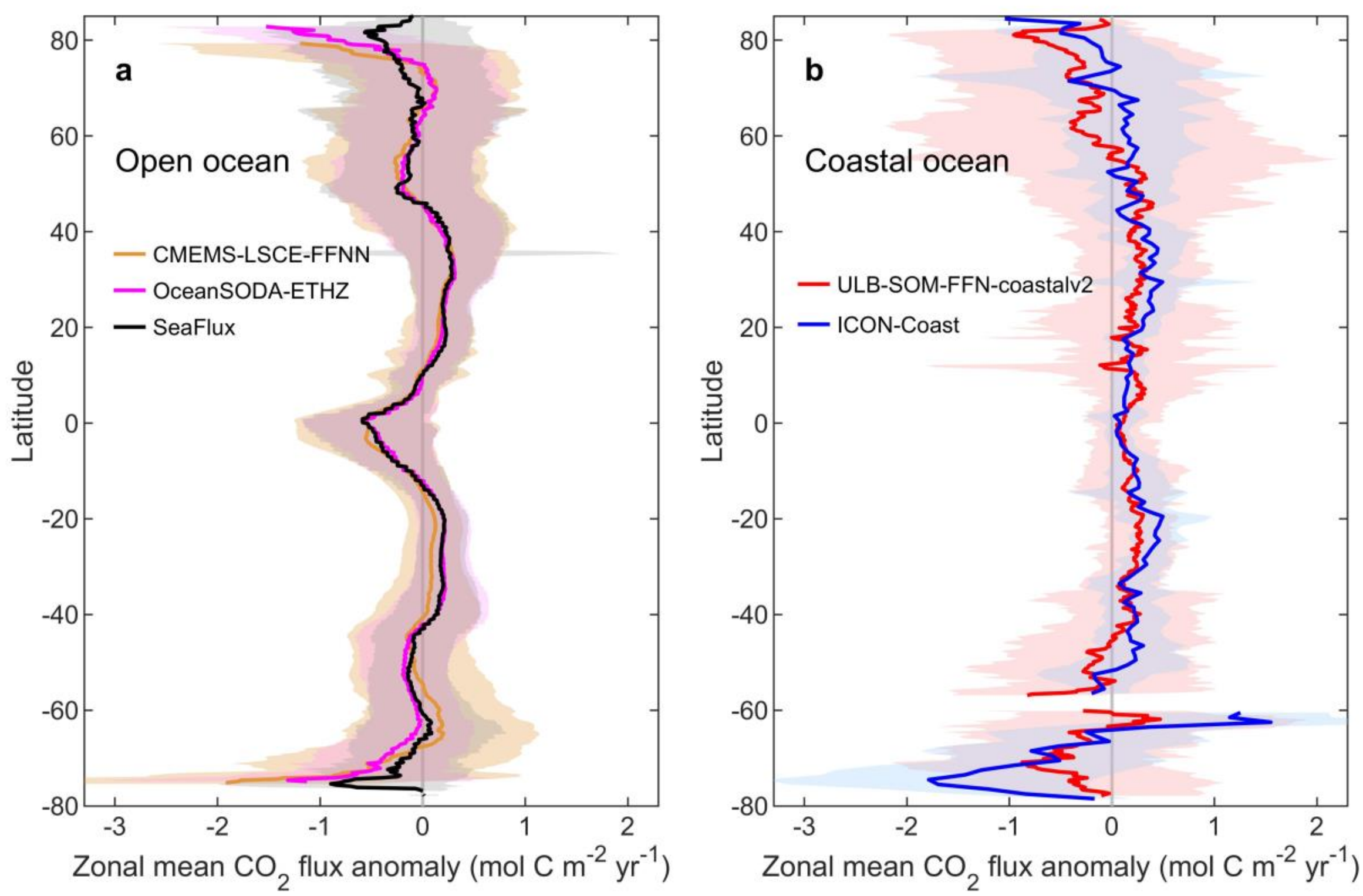

Supplementary Fig. 3 | Zonal mean CO_2 flux anomalies during MHW months (1985–2020) in the open ocean and coastal ocean. *(**a**) Open-ocean CO_2 flux anomalies derived from CMEMS-LSCE-FFNN, OceanSODA-ETHZ, and SeaFlux. (**b**) Coastal-ocean CO_2 flux anomalies derived from ULB–SOM–FFN–coastalv2 and ICON-Coast. Shading indicates ±1 standard deviation of the anomalies.*”

The figure is appropriately referenced in the revised manuscript: (Page 4, Lines 102–105) “*Positive anomalies, indicating enhanced CO_2 outgassing or reduced uptake, prevail in most coastal regions, while negative anomalies, representing enhanced uptake or reduced outgassing, are largely*

confined to high-latitude, ice-influenced shelf seas (Fig. 1a and Supplementary Fig. 3b)." (Page 6, Lines 152–156) "*In tropical regions, MHWs amplify coastal CO_2 outgassing but suppress open ocean outgassing*[50,51,62] *(Supplementary Fig. 3), despite both being climatological sources of atmospheric CO_2. At higher latitudes, polar and subpolar coastal shelf seas exhibit enhanced CO_2 uptake during MHW months, while uptake in the open North Pacific weakens*[50,51] *(Supplementary Fig. 3), although both are climatological CO_2 sinks.*"

In the zonal mean plots shown in Supplementary Fig. 3b, the $F\mathrm{CO}_2$ anomalies are reasonably represented by the ICON-Coast model. This figure is also appropriately referenced in the Model Evaluation section (Page 21, Lines 573–578): "*In terms of MHW-induced anomalies, the model reproduces the overall spatial pattern: most coastal regions are characterized by net upward FCO_2 anomalies (Supplementary Figs. 3b and 12), consistent with observation-based estimates in low- and mid-latitude shelves (Fig. 1a and Supplementary Fig. 3b). Towards higher latitudes, the magnitude of simulated FCO_2 anomalies decreases, and in some regions the sign shifts to a net downward flux (Supplementary Fig. 3b).*"

We add a comparison of zonal mean contributions from thermal and non-thermal $p\mathrm{CO}_{2,\mathrm{sea}}$ anomalies between observations and the model (Supplementary Fig. 14). We find that the model captures the overall latitudinal structure well. In the Model Evaluation section, the sentence now reads (Pages 21–22, Lines 586–588): "*Despite these biases, the spatial distributions and zonal mean plots of thermal and non-thermal $pCO_{2,sea}$ contributions show overall good qualitative agreement with observational estimates (Supplementary Figs. 13 and 14).*"

"

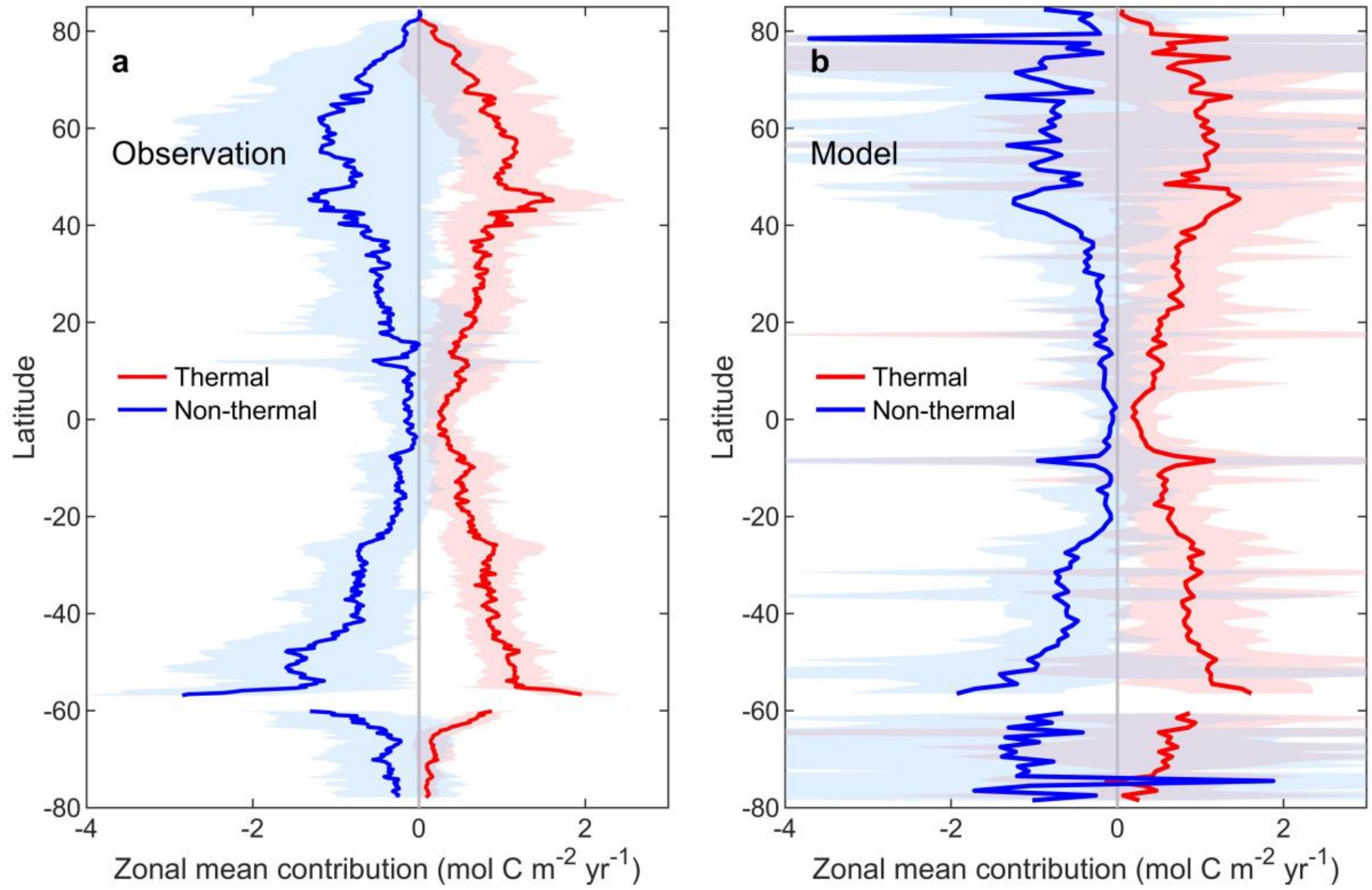

Supplementary Fig. 14 | Zonal mean contributions of thermal and non-thermal $pCO_{2,sea}$ components to FCO_2 anomalies during MHW months. *(**a**) Results derived from ULB–SOM–FFN–coastalv2. (**b**) Results derived from ICON-Coast. Shading denotes ±1 standard deviation of the anomalies.*"

We agree that the original discussion was not sufficiently comprehensive. In the revised manuscript, we have expanded the discussion related to biological carbon fixation anomalies presented in Results section, particularly regarding how MHW-induced changes in upper-ocean stratification affect phytoplankton, as follows (Pages 9–10, Lines 257–268): "*Phytoplankton, as a key driver of carbon fixation in marine ecosystems, plays an important role in carbon cycling*[76]*. Its response to MHWs can propagate through higher trophic levels, influencing zooplankton, fish, seabirds, and marine mammals*[77]*. The impact of MHWs on phytoplankton shows a clear latitudinal pattern, with chlorophyll generally declining in tropical and mid-latitude regions but increasing at high latitudes*[78]*. In nutrient-rich polar and subpolar regions with deep mixed layers, MHWs can directly alter upper-ocean stratification*[47,49,78,79]*, modifying light and nutrient availability and extending the residence time of phytoplankton in the euphotic zone*[77]*. Recent MHWs in the subpolar North Pacific have been associated with shifts in dominant phytoplankton functional groups*[80,81]*.*

In the Antarctic coastal ocean, MHWs can enhance stratification and trigger iron release from melting sea ice, fertilizing waters that are high in nutrients but low in chlorophyll, stimulating phytoplankton growth along the ice edge, and thereby enhancing CO_2 uptake[49,82]."

In the Discussion section, we have added a discussion on the quantitative uncertainty assessment, as follows (Pages 11–12, Lines 310–331): "*Roobaert et al.[11] highlighted that reconstructed coastal pCO_2 products may contain biases, particularly in regions with sparse observations or seasonal sea-ice cover. In three of the ten biogeochemical provinces (P1, P5, and P7), the root mean square error (RMSE) exceeds 35 μatm between reconstructed pCO_2 and SOCAT observations[11]. To evaluate the potential influence of such biases on our results, we propagated the upper-bound RMSE into the uncertainty of the air–sea CO_2 flux anomaly (Supplementary Note 1), thereby deriving a conservative estimate. When aggregated within each of the 45 coastal segments, the resulting uncertainty is relatively small compared with the estimated FCO_2 anomaly (Supplementary Fig. 10). When further aggregated across all coastal regions, the combined uncertainty remains limited (0.91 Tg C), largely because the large number of contributing grid cells reduces the overall uncertainty through statistical aggregation. In addition to reconstruction errors, the coastal flux product itself includes uncertainties associated with ΔpCO_2, gas transfer velocity, wind speed, and sea-ice cover. Roobaert et al.[11] estimated that the combined effect of these factors results in an uncertainty of approximately 0.01 Pg C yr^{-1} for the integrated coastal air–sea CO_2 flux. Although MHW months occur more frequently in recent decades with improved observational coverage, where uncertainties are lower than this long-term mean value[11], we adopt a conservative estimate by assuming temporal and spatial independence of uncertainties and scaling the variance according to the fraction of the coastal domain affected by MHW months (Supplementary Note 1). Under this assumption, the upper-bound uncertainty of 0.01 Pg C yr^{-1} corresponds to an uncertainty of 17.26 Tg C for the integrated CO_2 flux anomaly during MHW months. The resulting signal-to-noise ratio (SNR) is 2.38, suggesting that the enhanced coastal CO_2 uptake during MHW months remains relatively robust despite the reconstruction uncertainties.*"

I cannot tell if this manuscript fits to the scope of this journal and leave this decision to the editor. Personally, after reviewing, I have no problem seeing it published here as I don’t think that journal-based impact factors are a good proxy for good science.

Note to the Editor: I don’t find it helpful that this journal is using numbers and not author names for references. Please use author names, like almost every other journal in climate science.

Issue 1: MHW definition

A MHW is defined if the daily SST exceeds the 90% percentile of a fixed 30-year long daily climatology, without prior detrending (L534ff; and some modifications: L542ff).

A MHW month is a month 1) that has at least 15 MHW days based on daily SST and 2) in which the linearly detrended monthly averaged SST exceeds the monthly climatology based on detrended monthly averages SST.

The 1st condition is evaluated with eleven sensitivity runs (L662). What is the effect of the 2nd condition? Why is the 2nd condition based on linearly detrended data, but the calculation of the actual MHW days based on daily SST is not?

Response: We sincerely thank the reviewer for the comment. In the first version of the manuscript, we intentionally retained the SST trend to preserve the increasing frequency of MHWs. To maintain consistency with the subsequent decomposition of FCO_2 anomalies (which was relative to trend-preserving climatology), we introduced the 2nd condition for defining MHW months to exclude cases in which the monthly mean SST was lower than the climatology calculated from detrended SST in some regions. We now recognize that this approach introduced an inconsistency between the MHW definition and the CO_2 flux anomaly framework.

After careful consideration, we have revised our methodology and detrended SST prior to MHW detection. We define MHW months using a single criterion: a month is classified as an MHW month if it contains at least 15 MHW days. In addition, we have revised the fixed climatological reference period from 1985–2014 to 1985–2020. The sentences now read as follows: (Page 13, Lines 358–359) “*SST data during 1982–2020 were detrended prior to MHW detection to remove the long-term influence, and MHWs were subsequently identified over 1985–2020.*”, (Pages 14–15, Lines 384–391) “*To match the monthly resolution of the $pCO_{2,sea}$ and FCO_2 data, we defined*

MHW months by aggregating daily MHW metrics within each month, following the approach of Edwing et al.[28]. For each grid cell, the number of MHW days was calculated for each month during 1985–2020. A month was classified as an MHW month only if it contained at least 15 MHW days, ensuring that the thermal perturbations were strong enough to induce ecological impacts and influence monthly CO_2 fluxes[28,99]. This criterion ensured that MHW months represented statistically significant thermal anomalies (Supplementary Fig. 8e), while also maintaining a sufficient sample size for robust assessment of carbon cycle responses."

The revised time series of MHW months is shown below.

"

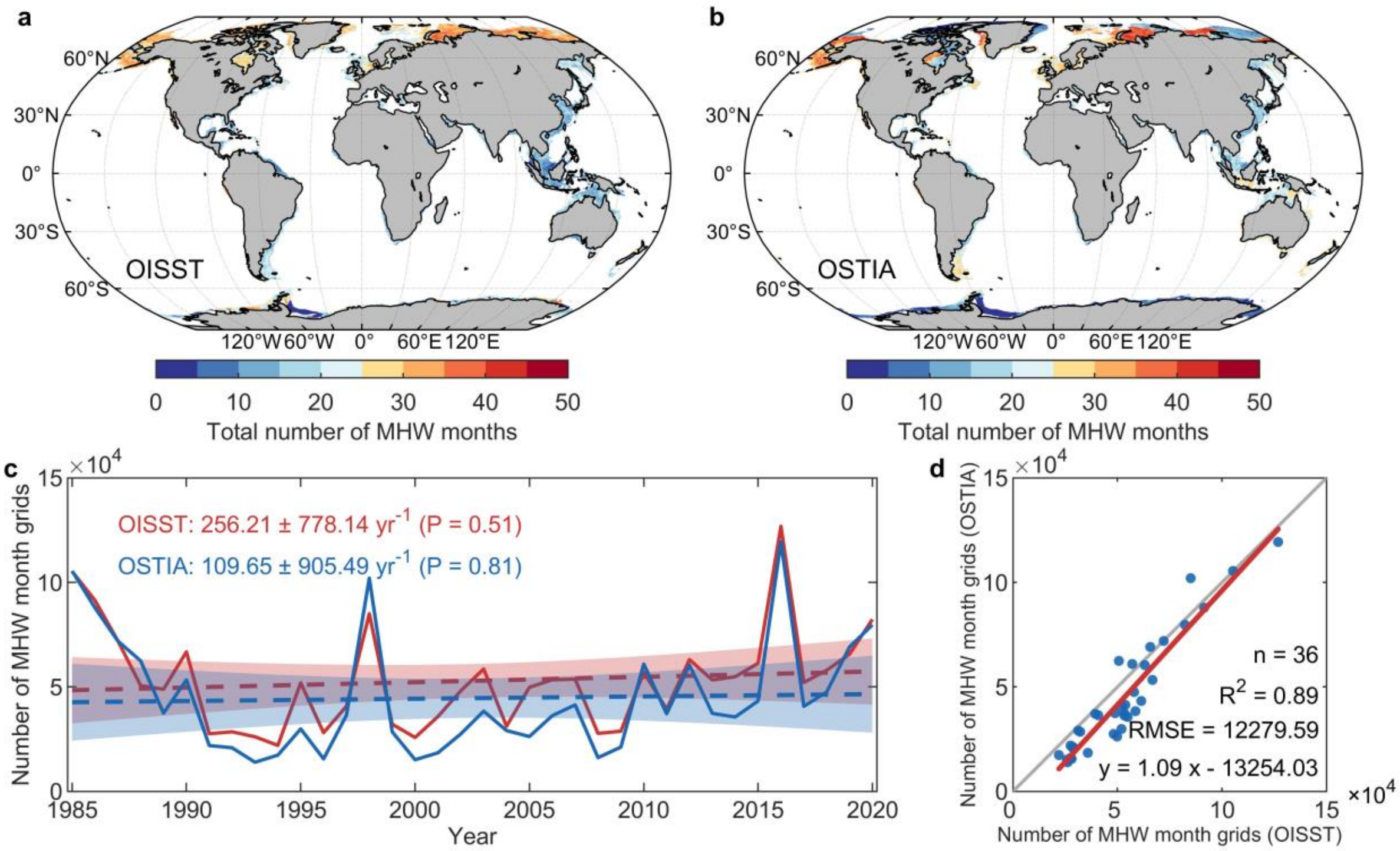

***Supplementary Fig. 1 | Consistency in MHW month detection between OISST and OSTIA datasets (1985–2020).** The global distribution of the total number of MHW months in coastal regions by the OISST dataset (**a**) and the OSTIA dataset (**b**). (**c**) Time series of the annual total number of grid cells identified as MHW months (OISST data in red and OSTIA data in blue). Dashed lines indicate linear trends, and shaded bands represent 95% confidence intervals. Trend slopes and corresponding P values are provided in the panel. (**d**) Scatterplot comparison of monthly grid cell counts between the two datasets. Data volume, R^2, RMSE, and regression lines are provided*."

All analyses have been recalculated accordingly. While detrending SST prior to MHW detection changes the time series figures and largely removes long-term trends, the main conclusions of the study remain robust, with only moderate quantitative adjustments, such as the change in the integrated CO2 flux anomaly from 4.3 ± 0.2% to 11.0 ± 1.6%.

An 'anomaly' is defined as the amplitude of a variable 'during MHW months'. How exactly was this calculated? I cannot find any information about this in the respective Methods section (L599). In fact, I find this whole section rather confusing than helpful as there the term 'anomaly' seems to denote a value with respect to any climatology, not the specific one during MHW months.

Response: The anomaly of carbon variables during MHW months refers to "the anomaly for MHW months" relative to the trend-preserving climatology. The climatology is calculated from the full time series, not solely from MHW months. Therefore, the resulting anomalies represent the contemporaneous impact of MHWs relative to the long-term climatological expectation.

For clarity, we have revised the Methods section as follows (Pages 16–17, Lines 446–454): "*Monthly anomalies were calculated to isolate the short-term impacts of MHWs while removing long-term trends associated with global warming and rising atmospheric CO_2. For each variable and grid cell, a trend-preserving climatology was first calculated over the full 1985–2020 time series, combining a long-term linear trend with a mean seasonal cycle derived from detrended data*[51,57]*. Anomalies during MHW months were then defined as the deviation of each MHW months from its corresponding climatological value. These anomalies specifically capture the instantaneous effects of MHWs relative to the long-term expectation. Consistent with this approach, all climatological fields in this study, denoted with an overbar, retained the long-term trend to ensure methodological consistency.*"

I don't understand L115: 'Given the particularly high frequency of MHW months in the Arctic (Extended Data Fig. 1a,b), we quantify the integrated FCO2 anomaly for the global coastal ocean by accounting for area and frequency.'. How do you conclude that there is a 'particularly high frequency' and how does the 'accounting for area and frequency' work? Please clarify.

Response: Thank you for pointing out this vague description. The statement regarding the "particularly high frequency" of MHW months in the Arctic is based on the spatial distribution shown in Supplementary Fig. 1a,b, where Arctic coastal regions exhibit more frequent MHW months compared to other regions. To calculate the integrated FCO_2 anomaly, for each grid cell and each month identified as an MHW month, the FCO_2 anomaly is multiplied by the corresponding area and converted to carbon units (Tg). The total integrated anomaly is then obtained by summing over all MHW months and all coastal grid cells.

The revised text now reads (Page 5, Lines 112–119): "*Given the strong regional heterogeneity in the frequency of MHW months across the global coastal ocean (Supplementary Fig. 1a,b), particularly the high frequency observed in the Arctic, we quantify the integrated FCO_2 anomaly for the global coastal ocean by accounting for both the frequency of MHW months and their spatial coverage. Specifically, for each grid cell and month identified as an MHW month, the FCO_2 anomaly is multiplied by the corresponding grid-cell area and converted to carbon units (Tg). The total integrated anomaly is then obtained by summing across all MHW months and all coastal grid cells, ensuring that both spatial coverage and MHW month occurrence are properly represented.*"

Issue 2: Model evaluation

I do think that your ocean hindcast simulation is suitable to investigate the coastal carbon cycle during MHW conditions. However, as mentioned in my summary above, I would like to see some evaluation of modeled MHW parameters.

Response: Thank you for your suggestion. As our analysis focuses on MHW months, we additionally evaluate the simulated MHW characteristics against OISST/OISSTIA estimates by comparing the spatial distribution of MHW-month occurrence and the temporal variability of the integrated annual MHW-month area. The integrated MHW-month area combines the effects of MHW frequency, duration, and spatial extent.

The following text is added to the Model Evaluation section (Page 21, Lines 557–570): "*MHW statistics are evaluated by comparing the simulated spatial distribution of MHW-month occurrence and the temporal variability of the integrated annual area affected by MHW months against OISST/OSTIA-based estimates. Overall, the model reproduces the global spatial*

distribution of MHW-month occurrence reasonably well, although the total frequency of MHW months in high-latitude regions is generally underestimated compared to observations (Supplementary Fig. 11a). This underestimation is likely related to the relatively thick uppermost model layer (~16 m), which dampens near-surface temperature extremes and reduces the occurrence of simulated MHWs relative to satellite-derived SST products representing the ocean skin layer. Despite these regional biases, the model reproduces the interannual variability of the integrated MHW-month area well (Supplementary Fig. 11b). The integrated MHW-month area combines the effects of MHW frequency, duration, and spatial extent, and therefore provides a comprehensive metric for evaluating the model performance. The close agreement between the simulated and observed time series of integrated MHW-month area supports the applicability of the model framework for investigating MHW-driven carbon cycle responses."

"

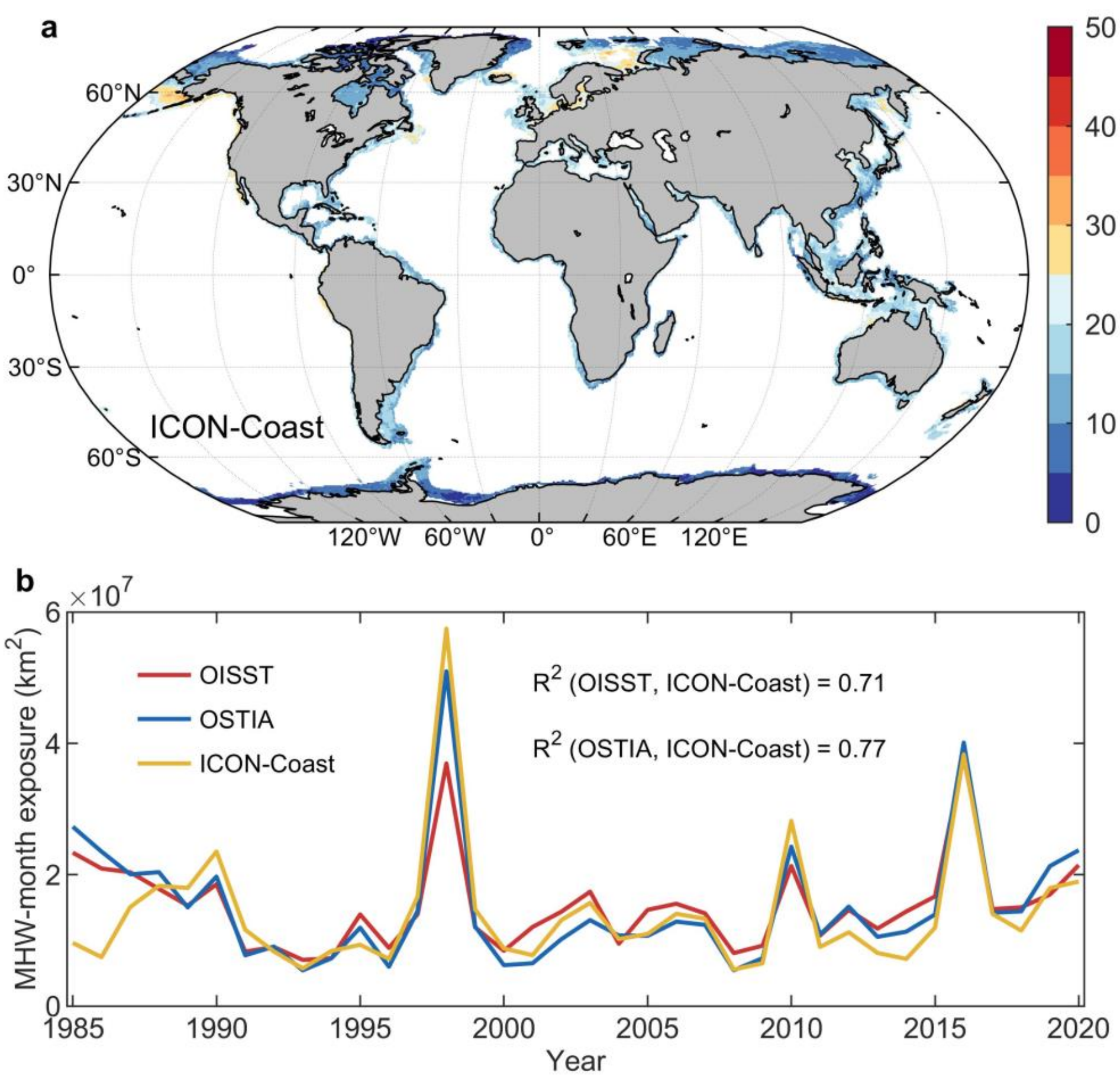

***Supplementary Fig. 11 | Model evaluation of MHW-month metrics. (a)** Global distribution of the total number of MHW months (1985–2020) in coastal regions simulated by ICON-Coast. **(b)** Time series of the integrated MHW-month area from OISST, OSTIA, and ICON-Coast, with R^2 values provided for comparisons between OISST and ICON-Coast, and between OSTIA and ICON-Coast.*"

L705: 'In terms of MHW-induced anomalies, the model reproduces the overall spatial pattern: most coastal regions are characterized by net upward FCO2 anomalies (Supplementary Fig. 2), consistent with observation-based estimates in low- and midlatitude shelves (Fig. 1a).' Yes but not in the polar latitudes, which → are the important regions of this study, by comparing Fig. 1a and Supplementary Fig. 2 by eye.

Response: In the first version of the manuscript, the model FCO_2 anomalies were calculated using OISST-derived MHW months. Following the reviewers' suggestions, we updated the analysis by identifying MHW months directly from the model daily SST output. With this revision, the simulated FCO_2 anomaly patterns show improved agreement with observations, including in polar regions (Supplementary Fig. 12). This consistency is also reflected in the zonally mean FCO_2 anomalies shown above (Supplementary Fig. 3b).

"

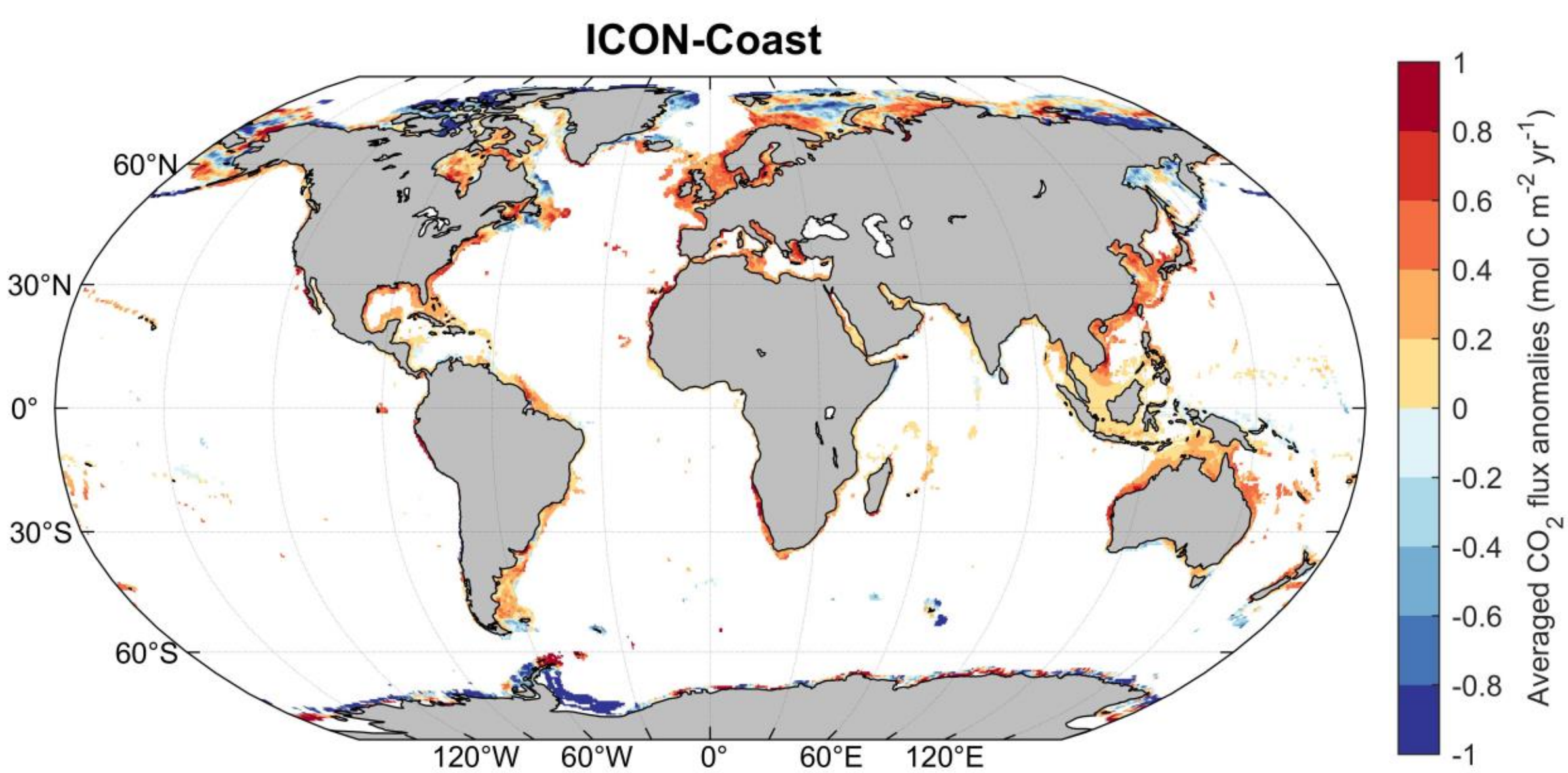

Supplementary Fig. 12 | Coastal FCO_2 responses to MHW months simulated by ICON-Coast. *Spatial distribution of averaged air–sea FCO_2 anomalies (mol C m^{-2} yr^{-1}) across the global coastal regions, derived from ICON-Coast simulations*."

Actually, ideally, I would like to see the results presented in Fig. 1, Fig. 2, and Extended Data Fig. 1-5 in a way that both data product and model estimates are directly comparable. At least, please add the model results to Fig. 1b.

Response: We thank the reviewer for this suggestion. This study primarily relies on observation-derived reconstructed datasets to quantify integrated coastal FCO_2 anomalies during MHW months and to identify their dominant drivers through decomposition analysis. The model is used in a complementary way to explore the role of biological processes in driving DIC variability.

Following the reviewers' comments, we have added additional comparisons of MHW between observational and model-based results and further strengthened the evaluation of the model's skill in simulating carbon uptake in the revised manuscript.

While we agree that a fully parallel presentation of observational and model products would be valuable, we aim to keep the main focus of the research clearly on the observational results to ensure clarity of the primary findings. The model results are therefore used primarily to support process understanding and mechanistic interpretation, rather than to mirror all observational figures.

Minor issues

Whole document: Please replace 'MHWs' by 'MHW months' if you speak about your analysis.

Response: We thank the reviewer for this suggestion. We agree that precise terminology is needed, as our analysis is based on MHW months, and we use FCO_2 anomalies during MHW months to represent MHW-related carbon exchange anomalies.

We have revised the manuscript to use "MHW months" when describing the analytical framework and results, while retaining "MHWs" only in contexts referring to the broader physical phenomenon.

L33: 'Although it covers only ~7% of the global ocean surface, it contributes disproportionately to the ocean carbon sink, with CO2 uptake rates far exceeding those of the open ocean[5-11].' → Coastal ocean CO2 uptake estimates per unit area are indeed larger than those of the open ocean with global averages of -0.68 and -0.58 mol C m^{-2} yr^{-1} versus -0.5 and -0.37 mol C m^{-2} yr^{-1} from Dai et al., 2022 (ref 7) and Roobaert et al., 2019 (ref 16), respectively. These numbers suggest a 36% and 57% higher CO2 uptake per unit area in coastal regions, and the latitudinal air-sea CO2 flux distribution averaged over the coastal and open ocean regions are actually quite similar (Figure 4a of Roobaert et al., 2019). Hence, I find the wording 'disproportionately' and 'far exceeding' too unspecific and too strong.

Response: We have revised the sentence to a more appropriate formulation (Page 2, Lines 33–35): "*Although it covers only ~7% of the global ocean surface, it contributes a relatively large share of the ocean carbon sink, with CO_2 uptake rates higher than those of the open ocean[5-11].*"

L35: 'Defined here as the continental shelf sea, the coastal ocean is estimated to absorb 0.18–0.45 Pg C yr^{-1} of atmospheric CO28. →Ref 8 is Laruelle et al., 2014. Please include more recent references in this estimate, e.g. Roobaert et al., 2019 (ref 16) and Dai et al., 2022 (ref 7). Also, please provide a similar area-integrated number for the open and/or total ocean so that the reader can set everything into perspective.

Response: Following the reviewer's suggestions, we have added more recent references (Roobaert et al.[1], Dai et al.[2] and Roobaert et al.[3]) to support the coastal carbon estimate, and we provide an area-integrated value for the total ocean to allow readers to place the coastal flux in a broader context.

The revised sentence now reads (Page 2, Lines 35–37): "*The total ocean currently absorbs approximately 2.4 ± 0.5 Pg C yr^{-1} of atmospheric CO_2[12]. By comparison, the coastal ocean, defined here as the continental shelf sea, is estimated to absorb 0.18–0.45 Pg C yr^{-1} [7,8,11,13].*"

L38: ‘This sink is highly heterogeneous in space, with polar and subpolar shelf seas contributing more than 90% of the total7.‘ → Yes, but this area-integrated value at least partially reflects the large area sum of the two polar regions compared to other regions (Figure 3 of Dai et al., 2022; ref 7). Here, I would find it more helpful to (additionally) report the relatively high CO2 uptake rates per unit area to understand the significance of the polar regions.

Response: We have revised the text to additionally report relatively high CO_2 uptake rates per unit area for polar and subpolar shelf seas. The sentence now reads (Page 2, Lines 39–42): “*This sink is highly heterogeneous in space, with polar and subpolar shelf seas showing substantially higher CO_2 uptake per unit area than other shelf seas and contributing more than 90% of the total uptake due to their large combined area*[7]*.*”

L45: ‘Long-term observations suggest that the coastal CO2 uptake has strengthened in recent decades, largely driven by enhanced biological carbon fixation3,25.’ → To me, this formulation implies that the latter point was shown in observations. However, ref 3 (Mathis et al., 2024) is a model study. Please reformulate. Also, as far as I understand Mathis et al., 2024, 41% (= 2/(2 + 2.8) gC m-2 yr-1; numbers from their Figure 5 c, d) of modeled FCO2 changes over the historical period averaged over the coastal oceans originate from CO2 solubility changes. The component ‘biological carbon fixation’ is in the remaining 59% (= 100% – 41%). This remaining part, however, also includes everything else, e.g. changes in circulation. After reading their ‘Analysis’ section it is somewhat unclear to me how they derived the other percentages reported in their Abstract. Hence, I am also somewhat against the current formulation ‘largely driven by enhanced biological carbon fixation’ as to me all components in their budget seem to be equally important. I have asked several colleagues if they understand the budget of Mathis et al., 2024 but nobody seems to be able to do so.

Response: We agree that the original formulation was not accurate. The sentence has been revised to (Page 3, Lines 47–49): “*Long-term observations suggest that the coastal CO_2 uptake has strengthened in recent decades*[26]*, while modeling experiments propose enhanced biological carbon fixation and increasing atmospheric CO_2 as the main drivers*[3]*.*”

In Mathis et al. 2024, the drivers for the increase in CO_2 uptake, other than the solubility component, are summarized as enhanced biological carbon fixation. This was done because all identified changes in the physical conditions rather induce a weakening of the CO_2 uptake, such as a stronger and shallower stratification, or enhanced eastern boundary upwelling of carbon-rich water masses.

L101: ‘The absence of significant anomalies in many regions does not indicate a lack of response but rather the coexistence competing mechanisms.’ → Nice formulation but somewhat unclear to me. Please give the respective figures and try to discuss this as one of my personal take homes of your study is that the effect of MHWs for the coastal air-sea CO2 flux is mostly negligible.

Response: We agree that the original statement was unclear. We have now added the corresponding figure (Supplementary Fig. 2d) and expanded the discussion to better explain this point as follows (Page 5, Lines 108–111): “*The absence of significant anomalies in many regions (Supplementary Fig. 2d) does not indicate a lack of response but rather the coexistence of competing mechanisms, as the net FCO_2 response to MHWs is strongly influenced by the “tug of war” between thermal and non-thermal drivers*[50,51,57]*.*”

In this section, this statement is primarily used to motivate the analysis of integrated CO_2 flux anomaly presented in the following paragraph. A more detailed discussion, together with the relevant figures, is provided in the subsequent section focusing on the drivers of coastal CO_2 exchange anomalies. There, we show that (Page 7, Lines 170–174) “*The thermal contribution of $pCO_{2,sea}$ consistently enhances fluxes by reducing solubility in warmer seawater during MHW months (Supplementary Fig. 7e), a response that is overall statistically significant (Supplementary Fig. 8e). However, the thermal forcing is often offset by non-thermal processes or reductions in sea ice cover, particularly in regions where mean FCO_2 anomalies are not statistically significant (Supplementary Fig. 2d).*”

Importantly, the fact that the thermal contribution is significantly different from zero indicates that MHWs do exert a measurable influence on coastal air–sea CO_2 fluxes. The weak or non-significant net anomalies in some regions do not imply a negligible effect but rather reflect compensation among multiple processes. This underscores the coastal ocean as a system in which interacting

drivers produce a buffered or regulated response under MHW conditions, rather than an absence of response.

Fig. 1b: Add meaning of numbers to caption. To me, the horizontal lines are not really ‘bold’.

Response: We have added an explanation of the numbers in the caption to clarify their meaning (Page 33, Line 920): “*Segment names and their serial numbers are shown.*”

Additionally, the horizontal lines have been bolded to enhance visibility.

Extended Data Fig. 3: Please add the meaning of negative FCO2 anomalies to the caption.

Response: We have clarified the meaning of negative anomalies in the caption by explicitly stating that (Page 5, Lines 46–47 in Supplementary Information) “*Negative anomalies indicate enhanced oceanic CO_2 uptake during MHW months.*”

L122: ‘Collectively, these results provide robust evidence that MHWs enhance coastal CO2 uptake. This response contrasts sharply with the open ocean, where MHWs suppress global CO2 uptake by ~8%50.’ Yes, but Figure 1 of Li et al. → 2024 (ref 50) shows that in four out of eight aggregated regions, and globally, there are purple points above and below the zero line, i.e. the open ocean air-sea CO2 flux during MHWs can be both, increased or weakened, depending on the pCO2 product. Please reformulate.

Response: In these two sentences, our goal is to highlight the contrast in the globally integrated CO_2 uptake anomaly between the open ocean and the coastal ocean. While regional responses can vary, as discussed later in the manuscript, our focus here is on the globally integrated signal, which shows an opposite sign between the two domains.

Globally, both positive and negative anomalies are indeed present in the final column of Figure 1 in Li et al.[4]. This includes the purple points, representing six observation-based pCO_2 products using an average wind product, as well as the orange points, which correspond to 30 individual observation-based data products. However, the conclusion reported in the Abstract of Li et al.[4] is

based on the ensemble of these 30 products and indicates an average global reduction of ~8% (3%–19% across datasets; mean ± 95% confidence interval) in open-ocean CO_2 uptake during MHWs.

To avoid overstating the open-ocean response, we have revised the text to explicitly refer to the integrated estimate and to acknowledge that the impact of MHWs on open-ocean integrated air–sea CO_2 flux varies in both sign and magnitude across $p$$CO_2$ products, while still emphasizing the contrast in the globally integrated signal.

The revised sentences now read: (Page 5, Lines 124–125) "*Collectively, these results provide robust evidence that MHWs enhance integrated coastal CO_2 uptake.*" and (Page 5, Lines 131–133) "*This response contrasts sharply with the open ocean, where MHWs suppress integrated global CO_2 uptake by ~8% on average, although the sign and magnitude of the response vary across products*[50]*.*"

L124: 'Although rising temperatures are generally known to reduce the solubility of CO2 in seawater and thus promote outgassing[58], our findings indicate that the integrated net response of coastal systems is the opposite, implying stronger compensating feedback in the coastal ocean relative to the open ocean.' → Again, please acknowledge Li et al. 2024 (ref 50); they show a strong regional compensation of thermal and non-thermal contributions to FCO2 anomalies during MHWs (their Figure 3). In fact, except for the polar latitudes, your Fig. 2 and their Figure 3 show more or less the same (comparing your coastal and their adjacent open ocean areas).

Response: We have acknowledged Li et al.[4] as suggested. Our intent here is to emphasize the contrast in the globally integrated CO_2 uptake anomaly between the coastal and open oceans, and to motivate the subsequent analysis of the 45 coastal segments and their dominant drivers. Here, the term "stronger compensating feedback" refers to a stronger tug of war between non-thermal and thermal drivers.

While our Fig. 2 and Figure 3 of Li et al.[4] show broadly similar regional patterns in some areas, our focus is on the regions contributing most strongly to the integrated response. In coastal systems, thermal effects dominate CO_2 flux variability in tropical source regions (e.g., Northern Australia and Southeast Asia), whereas in high-latitude coastal sink regions, sea-ice loss and non-thermal $pCO_{2,sea}$ processes outweigh thermal forcing. By contrast, in the open ocean, non-thermal DIC

advection dominates in the tropical Pacific, while thermal forcing is the primary driver in low–mid latitude North and South Pacific sink regions[4,5]. These contrasts highlight distinct coastal versus open-ocean responses, even in regions with similar climatological CO_2 roles, reinforcing the integrated contrast emphasized in our study.

L137: ‘On an interannual timescale, anomalies in polar and subpolar shelf seas show significantly stronger correlations ($P \leqslant 0.003$) with global integrated FCO2 anomalies than other regions (Extended Data Fig. 4a). These results demonstrate that the global enhancement of coastal CO2 uptake during MHWs is dominated by the polar and subpolar regions.’ → 1) To me, the covariability of global and polar on the one hand and global and tropical FCO2 anomalies is statistically identical (‘Polar’ and ‘Tropical’ in Extended Data Fig. 4a). 2) Shouldn’t the larger surface area of the subpolar and polar areas be taken into account? I expect that the same analysis with spatially averaged FCO2 anomalies, i.e. per unit area, yield different results (again Figure 3 of Dai et al., 2022, ref 7).

Response: We have revised the methodology by detrending SST prior to the detection of MHW months. Accordingly, the correlations between the integrated CO_2 flux anomalies for the entire coastal ocean and those for the seven classes have been updated. The revised correlation coefficients are now 0.70 for the subpolar region, 0.61 for the polar region, and 0.51 for the tropical region, indicating a clearer and more robust dominant role of the subpolar and polar regions (Supplementary Fig. 6a).

“

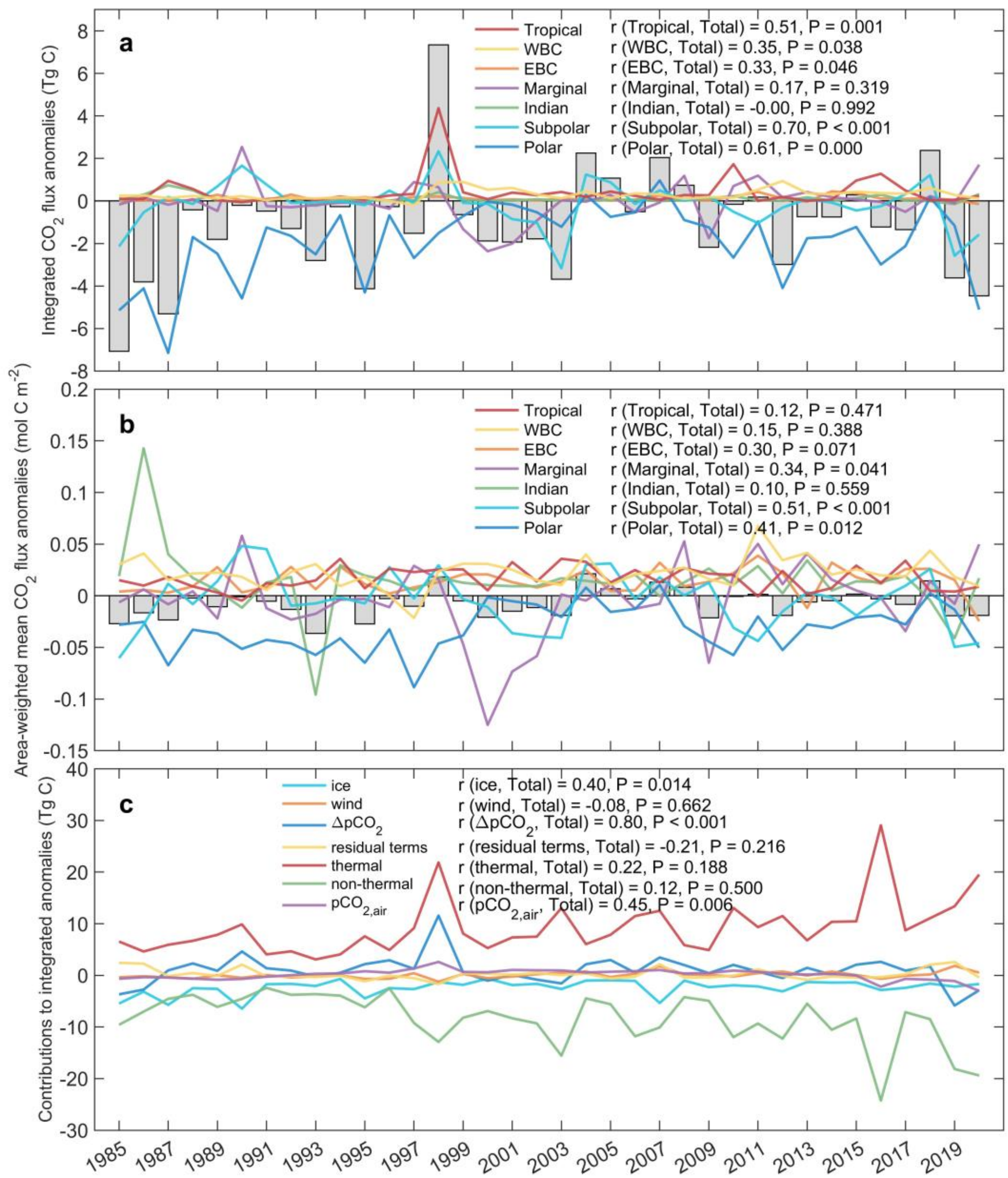

Supplementary Fig. 6 | Temporal dominance of regions and factors contributing to integrated FCO_2 anomalies. *(**a**) Time series of integrated FCO_2 anomalies for the global coastal ocean (bars) and across seven distinct regional classes. Correlation coefficients and P values are provided. (**b**) same as (**a**), but for area-weighted mean FCO_2 anomalies. (**c**) Time series of integrated FCO_2 anomalies driven by seven factors, with correlation coefficients and P values indicated. All data shown are derived from the observation-based ULB–SOM–FFN–coastalv2 dataset[1].*"

Regarding the reviewer's suggestion to consider spatially averaged FCO_2 anomalies, we explored two approaches. First, we divided the integrated CO_2 flux anomalies by the total area of each

region. As expected, this scaling does not affect the correlation coefficients. Second, we calculated area-weighted mean CO_2 flux anomalies and then evaluated the correlations between the global time series and those of the seven regions (Supplementary Fig. 6b), as shown above. This second approach yields same results, with the highest correlation found for the subpolar region (0.51), followed by the polar region (0.41).

The revised sentences now read (Page 6, Lines 147–149): "*On interannual timescales, both integrated and area-weighted mean FCO_2 anomalies in the polar and subpolar shelf seas exhibit significantly stronger correlations ($P < 0.05$) with global anomalies than those in other regions (Supplementary Fig. 6a, b).*"

Eq. 3: This is not yet finished as e.g. the first two terms on the RHS, $\bar{a}\bar{b}\bar{c} - \overline{\bar{a}\bar{b}\bar{c}}$, vanish. Are those what you call the 'zero-order' terms? If yes, what does it mean if these 'baseline contributions from mean states of the drivers' (L632) vanish? The brackets are redundant.

Response: Thank you for the comment. In our formulation, the overbar denotes the climatological state including the long-term trend rather than a simple temporal mean. Therefore, $\overline{\bar{a}\bar{b}\bar{c}}$ is not equal to $\bar{a}\bar{b}\bar{c}$, and these terms do not cancel. We have clarified the definition of the overbar as follows (Page 17, Lines 470–471): "*The anomalies a', b', c' are defined relative to their climatological states ($\bar{a}, \bar{b}, \bar{c}$), which include the long-term trend.*"

The redundant brackets have been removed as suggested.

Eq. 4-7: In Eq. 4, can you please somehow mark or group those first-order terms that represent the FCO2 anomaly contribution of a specific driver, e.g. mark/group all sea ice terms and so on, perhaps as Couldrey et al., 2016 (ref. 94) did.

Response: We have grouped all terms in Eqs. 3 and 4 as suggested, which allows readers to easily identify the contribution of each driver to the FCO_2 anomaly, as shown below (Page 17, Lines 463–466 and Page 18, Lines 476–483):

"$y' = \bar{a}\bar{b}\bar{c} + a'\bar{b}\bar{c} + \bar{a}b'\bar{c} + \bar{a}\bar{b}c' + \bar{a}b'c' + a'\bar{b}c' + a'b'\bar{c} + a'b'c' - \overline{\bar{a}\bar{b}\bar{c} + a'\bar{b}\bar{c} + \bar{a}b'\bar{c} + \bar{a}\bar{b}c' + \bar{a}b'c' + a'\bar{b}c' + a'b'\bar{c} + a'b'c'}$

$$= \underbrace{\bar{a}\bar{b}\bar{c} - \overline{\bar{a}\bar{b}\bar{c}}}_{zero-order\ term} + \underbrace{a'\bar{b}\bar{c} - \overline{a'\bar{b}\bar{c}} + \bar{a}b'\bar{c} - \overline{\bar{a}b'\bar{c}} + \bar{a}\bar{b}c' - \overline{\bar{a}\bar{b}c'}}_{first-order\ terms} +$$

$$\underbrace{\bar{a}b'c' - \overline{\bar{a}b'c'} + a'\bar{b}c' - \overline{a'\bar{b}c'} + a'b'\bar{c} - \overline{a'b'\bar{c}}}_{second-order\ terms} + \underbrace{a'b'c' - \overline{a'b'c'}}_{third-order\ term} \quad (3)”$$

$$“FCO_2' = \underbrace{\left(\overline{(kK_0)}\,\overline{(1-\mathrm{ice})}\,\overline{\Delta pCO_2} - \overline{\overline{(kK_0)}\,\overline{(1-\mathrm{ice})}\,\overline{\Delta pCO_2}}\right)}_{zero-order\ term} +$$

$$\underbrace{\left((kK_0)'\,\overline{(1-\mathrm{ice})}\,\overline{\Delta pCO_2} - \overline{(kK_0)'\,\overline{(1-\mathrm{ice})}\,\overline{\Delta pCO_2}}\right)}_{wind\ speed\ contribution} +$$

$$\underbrace{\left(\overline{(kK_0)}\,(1-\mathrm{ice})'\,\overline{\Delta pCO_2} - \overline{\overline{(kK_0)}\,(1-\mathrm{ice})'\,\overline{\Delta pCO_2}}\right)}_{ice\ contribution} +$$

$$\underbrace{\left(\overline{(kK_0)}\,\overline{(1-\mathrm{ice})}\,\Delta pCO_2' - \overline{\overline{(kK_0)}\,\overline{(1-\mathrm{ice})}\,\Delta pCO_2{}'}\right)}_{\Delta pCO_2\ contribution} +$$

$$\underbrace{\left(\overline{(kK_0)}\,(1-\mathrm{ice})'\,\Delta pCO_2' - \overline{\overline{(kK_0)}\,(1-\mathrm{ice})'\,\Delta pCO_2'}\right)}_{second-order\ term} +$$

$$\underbrace{\left((kK_0)'\,\overline{(1-\mathrm{ice})}\,\Delta pCO_2' - \overline{(kK_0)'\,\overline{(1-\mathrm{ice})}\,\Delta pCO_2'}\right)}_{second-order\ term} +$$

$$\underbrace{\left((kK_0)'(1-\mathrm{ice})'\,\overline{\Delta pCO_2} - \overline{(kK_0)'(1-\mathrm{ice})'\,\overline{\Delta pCO_2}}\right)}_{second-order\ term} +$$

$$\underbrace{\left((kK_0)'(1-\mathrm{ice})'\Delta pCO_2' - \overline{(kK_0)'(1-\mathrm{ice})'\Delta pCO_2'}\right)}_{third-order\ term} \quad (4)”$$

Then, in eq. 4, you plug in pCO’2,X for X = (T, DIC, ALK, SSS) (eq. 5, 7) for all pCO’2,sea terms in the ΔpCO2’ = pCO’2,sea – pCO’2,air terms, right? If that is correct, I count 3 ΔpCO2’ terms (from eq. 4) times 4 pCO’2,X terms (from eq. 5, 7) = 12 first-order pCO’2,X contribution terms to FCO2 anomalies, correct? Please make this clearer in the respective Methods section(s) as currently it’s not entirely clear to me.

Response: We only substitute the terms $pCO_{2,T}'$ and $pCO_{2,NT}'$ into the first-order terms in Eq. (4). The terms $pCO_{2,X}'$ (X = DIC, ALK, or SSS) are used only to determine which component is most important for $pCO_{2,NT}'$. In our framework, we first assess the importance of the non-thermal component based on the ULB–SOM–FFN–coastalv2 dataset (Fig. 2), and then identify which of DIC, ALK, or SSS contributes most to $pCO_{2,NT}'$ based on CMEMS-LSCE dataset (Fig. 4a–d).

To clarify this, we have added the following text in this part (Page 19, Lines 504–506): "*To quantify the thermal and non-thermal contributions to FCO_2', the terms $pCO'_{2,T}$ and $pCO'_{2,NT}$ were substituted into the first-order terms in equation (4), where ΔpCO_2 is the difference between $pCO_{2,sea}$ and $pCO_{2,air}$.*"

L164ff & Extended Data Fig. 4: Why do the thermal and non-thermal contributions exhibit a temporal trend but not the other components? Initially I thought that this is because of the MHW months definition in this study that does not remove the temporal trend of the underlying SST time series. But on the other hand I would expect that all time series show some kind of temporal trend due to climate change. Please clarify.

Response: In the first version of the manuscript, SST was not detrended prior to MHW detection. As a result, the identified MHW months were more concentrated in the later part of the time series, which introduced an apparent temporal trend in the thermal and non-thermal contributions. These two components exhibit larger magnitudes compared to the other components (Fig. 2), and their long-term trends were therefore more visible in the time series. In contrast, the other components have much smaller magnitudes (Fig. 2), which explains why no clear temporal trend was observed. This difference is thus mainly related to the relative magnitude of the components rather than a direct expression of long-term climate change.

In the revised version of the manuscript, SST has been detrended prior to MHW detection. With this updated methodology, the temporal trends in both thermal and non-thermal contributions are largely reduced (Supplementary Fig. 6c), indicating that the previously observed trends were primarily linked to the MHW month selection based on a non-detrended SST time series. This revision ensures that the identified MHW months represent short-term, instantaneous impacts rather than long-term warming.

L196: 'Open oceans thus exhibit patterns opposite to those of coastal systems with similar climatological roles.' No, → see comment to L124.

Response: As noted in the response of L124, our focus here is on the regions contributing most strongly to the integrated response. In coastal systems, thermal effects dominate CO_2 flux

variability in tropical source regions (e.g., Northern Australia and Southeast Asia), whereas in high-latitude coastal sink regions, sea-ice loss and non-thermal $p\mathrm{CO}_{2,\mathrm{sea}}$ processes outweigh thermal forcing. By contrast, in the open ocean, non-thermal DIC advection dominates in the tropical Pacific, while thermal forcing is the primary driver in low–mid latitude North and South Pacific sink regions[4,5]. These contrasts highlight distinct coastal versus open-ocean responses, even in regions with similar climatological CO_2 roles.

Fig. 3: In a and c, what means 'integrated sea ice fraction anomaly'? Please add units to figure and/or caption.

Response: In Figs. 3a and 3c, "integrated sea ice fraction anomaly" refers to the sum of sea ice fraction anomalies during MHW months. Since sea ice fraction is dimensionless (ranging from 0 to 1), the integrated anomaly is also unitless.

We have clarified this definition in the figure caption and now indicate that the values are dimensionless, described as (Page 35, Line 939) "*sum of anomalies during MHW months; unitless*".

L241: Please add 'coastal' somewhere.

Response: Added as suggested.

Fig. 4: Why the weird units in a-d? Why not simply using the spatial average, i.e. dividing by the respective area to obtain typical μatm? Please add to the caption that a-d are results from a data product but e-h model results. Why do you explicitly write 'The SST contributions are also shown in the fifth column'? I do not fully understand the carbon budget. Can you please very briefly describe the terms in the respective Methods section (L724)? I find the advection and 'decomp' terms particularly unclear. What means 'integrated carbon budget anomalies'? Integrated over time? That's why the budget units are not per time as they should? Please clarify.

Response: Our initial intention was to account for the area, but we now agree that presenting spatially average values would make the results clearer and easier for readers to interpret.

Accordingly, in the revised manuscript, we have recalculated the values in Fig. 4a–d and now present them in units of μatm.

“

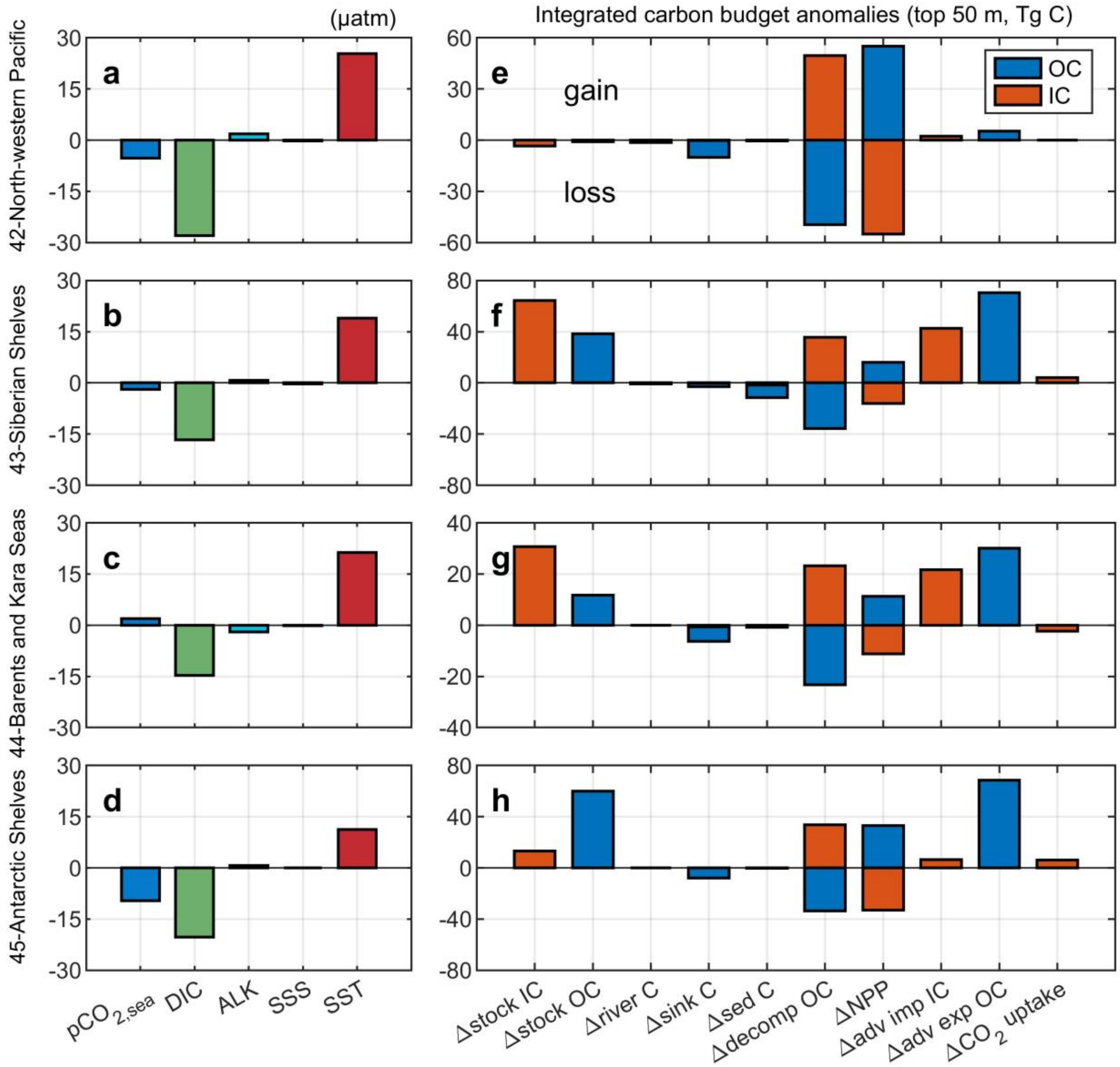

***Fig. 4 | Mechanisms driving non-thermal $pCO_{2,sea}$ anomalies and coastal carbon budget during MHW months from 1985 to 2020. a–d**, Relative contributions of DIC, ALK, and SSS to non-thermal $pCO_{2,sea}$ anomalies in four typical coastal regions: (**a**) the North-western Pacific, (**b**) the Siberian Shelves, (**c**) the Barents and Kara Seas, and (**d**) the Antarctic Shelves, derived from the observation-based CMEMS-LSCE dataset[66]. The SST contributions are also shown in the fifth column. **e–h**, Integrated anomalies (Tg C) in the upper-ocean (top 50 m) carbon budget of inorganic carbon (IC) and organic carbon (OC) during MHW months for the same regions, based*

on the ICON-Coast model[56]. Anomalies in the OC or IC stocks result from anomalies in river inputs, vertical sinking of particulate carbon, sedimentation of particulate carbon, decomposition of particulate and dissolved OC, net primary production, advective import of IC and advective export of OC (including anomalies due to circulation and vertical mixing), and CO_2 uptake from the atmosphere."

We have updated the caption to clarify that Fig. 4a–d show results from CMEMS-LSCE dataset, whereas Fig. 4e–h show results from the ICON-Coast model. In addition, all other figure captions, including those in the Supplementary Information, have been revised to clearly indicate whether the data are observational or model-based. Phrases such as "*All data shown are derived from the observation-based ULB–SOM–FFN–coastalv2 dataset*", "*derived from the observation-based CMEMS-LSCE dataset*", or "*based on the ICON-Coast model*" have been used to ensure that this information is clear and easily understood by readers.

In Fig. 4, our intention is to illustrate the mechanisms driving non-thermal $pCO_{2,sea}$ anomalies (DIC, ALK, and SSS). In some regions, such as the Siberian Shelves (43) and the Barents and Kara Seas (44), the magnitude of the SST contribution can even exceed that of DIC. However, our primary focus is on non-thermal $pCO_{2,sea}$ anomalies. In Fig. 2, we show the contribution of ice reduction and non-thermal $pCO_{2,sea}$ anomalies offset the influence of high SST. Therefore, we explicitly note that "The SST contributions are also shown in the fifth column" solely to provide a complete picture for constructing total $pCO_{2,sea}$, while clarifying that SST anomaly is not a component of non-thermal $pCO_{2,sea}$ anomalies.

In the upper 50m carbon budget presented in this study, physical changes in the circulation and vertical mixing are included in the terms "advective IC import" or "advective OC export". To be more clear, we add an explanation of the individual budget terms to the caption of Fig. 4 (Pages 36–37, Lines 951–955): "*Anomalies in the OC or IC stocks result from anomalies in river inputs, vertical sinking of particulate carbon, sedimentation of particulate carbon, decomposition of particulate and dissolved OC, net primary production, advective import of IC and advective export of OC (including anomalies due to circulation and vertical mixing), and CO_2 uptake from the atmosphere.*"

L270: Please clarify that the 8% and -0.5% are model results.

Response: Clarified as suggested.

L290ff: I find it a bit hard to follow. I wonder if it would be helpful to summarize Fig. 4 and Extended Data Fig. 8 for polar/tropical/non-polar shelves or climatological uptake/release shelves or such?

Response: In the Results section, we have already presented and discussed enhanced biological carbon fixation in polar and subpolar regions. Therefore, the aim of this first paragraph of the Discussion section is to highlight the contrasting patterns in typical tropical and WBC shelf seas, where FCO_2 anomalies are primarily dominated by thermal effects (Fig. 2f,g,i,j). Here, we use the contrast between Fig. 4 and new Supplementary Fig. 9 to highlight that oceanic background nutrient levels regulate phytoplankton responses to MHWs, with weaker blooms in oligotrophic waters and stronger ones in nutrient-rich waters[6-10].

We have revised the entire paragraph as follows (Page 10, Lines 278–289): "*In typical tropical and WBC shelf seas, where FCO_2 anomalies are dominated by thermal effects (Fig. 2f,g,i,j), the negative non-thermal $pCO_{2,sea}$ anomalies are nevertheless still primarily driven by negative DIC anomalies (Supplementary Fig. 9a–d). However, the biological responses to MHWs in tropical and WBC shelf seas differ fundamentally from those at high latitudes. Overall, advective transport of IC plays the dominant role, whereas the contribution of biological carbon fixation depends strongly on the background nutrient conditions of the ocean. In tropical shelf seas, MHWs suppress net biological carbon fixation by elevating water temperatures and reducing nutrient supply (Supplementary Fig. 9f,g). In contrast, the suppression is weaker in WBC regions, and net productivity is even enhanced in the China Sea and Kuroshio region (Supplementary Fig. 9e,h). Tropical shelf seas are generally nutrient poor[83-85], providing unfavorable conditions for intense phytoplankton blooms during MHWs[86-88], whereas WBC regions are generally nutrient-rich[89-91], supporting favorable conditions for phytoplankton blooms[67].*"

L292: 'by because'?

Response: We have corrected the text by removing the redundant word "by".

L530: Please drop a word or two what what 'foundation SST' and 'reprocessed' means from the OSTIA data product.

Response: We have clarified the terms as follows (Page 13, Lines 353–356): "*OSTIA reprocessed (produced by re-running the analysis system on historical observations to generate a consistent long-term climate record) product offers daily, gap-free foundation SST (temperature below the diurnal warm layer and free of diurnal variability) and sea ice concentration data at a higher spatial resolution of 0.05° × 0.05°[95,96].*"

L533: Please add the spatial interpolation method.

Response: As suggested, we have added the spatial aggregation method (Page 13, Line 357) "*using grid-cell averaging*".

L570: Wanninkhof (2014; ref 92) does not show this.

Response: Thank you for pointing out this mistake. As this is a well-established theoretical concept, we have removed the previous reference.

L639: In my view, the references for this statement are too unspecific (Sarmiento and Gruber 2006; ref 17) and not suitable (Edwing et al. 2024; ref 27). Please find a better reference.

Response: We have removed these two references, and we cite a more appropriate reference (Takahashi et al.[11]) (Page 18, Line 491).

L658: Please add 'shelf regions' or such to the numbers 12-16 and 41-45.

Response: Added as suggested.

L836: Add SeaFlux version. Why is pCO2,sea not listed under 'Available variables' of SeaFlux?

Response: We have added the SeaFlux version "*version 2021.04*" (Page 19, Line 124 in Supplementary Information) as suggested. We have also included all available SeaFlux variables: "*$pCO_{2,sea}$; $pCO_{2,air}$; k; K_0; ice*".

L838: Please add the spatial interpolation method.

Response: As suggested, we have added the spatial aggregation method "*using grid-cell averaging*" (Page 20, Line 126 in Supplementary Information).

L866: What is the 'climatological sum'?

Response: For MHW months, the expected climatological CO_2 flux, including the long-term trend, was multiplied by the area of each grid cell and summed over the global coastal ocean (Tg). We have replaced "climatological sums" with (Page 5, Line 50 in Supplementary Information) "*corresponding integrated climatological flux*".

References

Dai et al., 2022: https://doi.org/10.1146/annurev-earth-032320-090746

Li et al., 2024: https://doi.org/10.1029/2024GL110379

Roobaert et al., 2019: https://doi.org/10.1029/2019GB006239

References for Responses to Reviewer #3

1. Roobaert, A. et al. The spatiotemporal dynamics of the sources and sinks of CO_2 in the global coastal ocean. *Glob. Biogeochem. Cycles* **33**, 1693–1714 (2019).
2. Dai, M. et al. Carbon fluxes in the coastal ocean: synthesis, boundary processes, and future trends. *Annu. Rev. Earth Planet. Sci.* **50**, 593–626 (2022).
3. Roobaert, A., Regnier, P., Landschützer, P. & Laruelle, G. G. A novel sea surface pCO_2-product for the global coastal ocean resolving trends over 1982–2020. *Earth Syst. Sci. Data* **16**, 421–441 (2024).

4. Li, C., Burger, F. A., Raible, C. C. & Frölicher, T. L. Observed regional impacts of marine heatwaves on sea-air CO_2 exchange. *Geophys. Res. Lett.* **51**, e2024GL110379 (2024).
5. Mignot, A. et al. Decrease in air-sea CO_2 fluxes caused by persistent marine heatwaves. *Nat. Commun.* **13**, 4300 (2022).
6. Hayashida, H., Matear, R. J. & Strutton, P. G. Background nutrient concentration determines phytoplankton bloom response to marine heatwaves. *Glob. Change Biol.* **26**, 4800–4811 (2020).
7. Zhan, W. et al. Reduced and smaller phytoplankton during marine heatwaves in eastern boundary upwelling systems. *Commun. Earth Environ.* **5**, 629 (2024).
8. Li, M. et al. Phytoplankton spring bloom inhibited by marine heatwaves in the North-Western Mediterranean Sea. *Geophys. Res. Lett.* **51**, e2024GL109141 (2024).
9. Zheng, J. et al. Phytoplankton response to the record-breaking marine heatwave in the summer of 2020 in the South China Sea. *J. Geophys. Res. Oceans* **129**, e2024JC021275 (2024).
10. Bian, C., Zhao, Z., Holbrook, N. J., Strutton, P. G. & Wu, L. Marine heatwaves shift ocean net primary productivity from the tropics toward the poles. *Nat. Commun.* **17**, 4624 (2026).
11. Takahashi, T. et al. Climatological mean and decadal change in surface ocean pCO_2, and net sea–air CO_2 flux over the global oceans. *Deep Sea Res. Part II Top. Stud. Oceanogr* **56**, 554–577 (2009).

Response to the reviewers' comments (second round)

We sincerely thank the reviewers for their positive evaluation of our revised manuscript and for their valuable additional comments. We appreciate the opportunity to further improve the manuscript and have carefully addressed all remaining comments through additional revisions. Detailed point-by-point responses to each comment (shown in black) are provided below. Our responses are presented in blue, and the corresponding changes in the revised manuscript are highlighted in italic and underlined text.

Reviewer #1 (Remarks to the Author):

My comments from the previous round of reviews have been satisfactorily addressed.

Response: We thank the reviewer for the positive assessment of our revised manuscript. We are pleased that the reviewer considers the revisions satisfactory and has no further concerns.

Reviewer #2 (Remarks to the Author):

I thank the authors to have made a major revision, and to have clearly separate trends from HW events. I find the detailed response and the revised manuscript and supp. Mat. really appropriate.

One very small comment:

Line460, when mentioning y=abc as a framework, indicate that this is a good framework for equation (1), where y is FCO2… (I belive that lines 472-475 are fine where they are)

Response: We sincerely thank the reviewer for the positive assessment of our revised manuscript and for this helpful suggestion.

Following the reviewer's recommendation, we have clarified that the $y = abc$ framework is applied to equation (1). We have added the following text (Page 17, Line 472): "*This framework is applicable to equation (1), where y represents FCO_2.*"

<u>Reviewer #3 (Remarks to the Author):</u>

Review 2 of Nature Communications ID NCOMMS-25-92847-T: 'Polar and subpolar shelf seas dominate enhanced coastal CO_2 uptake during marine heatwaves' by Zhang et al.

Table of Contents

Summary..1
Minor...1
References..9

<u>Summary</u>

Thanks a lot Zhang et al. for working on all comments of the 1st review. I am especially thankful for the additional Supplementary Fig. 3. To me, that confirms my initial impression that the effect of MHWs on coastal and open ocean air-sea CO2 uptake is actually very similar. Hence, personally, I would formulate '… in stark contrast ...' less strongly in the abstract (L22; see also comment to L152 below). I am however fine with the current formulation.

I have some remaining points which are all minor. If these are addressed I am happy to see this published.

Response: We sincerely thank the reviewer for the encouraging comments and for this helpful suggestion.

We agree that the wording in the Abstract can be moderated. Accordingly, we have revised "in stark contrast to" to "*<u>in contrast to</u>*" (Page 2, Line 22) in the Abstract.

<u>Minor</u>

L35: 'Although it covers only ~7% of the global ocean surface, it contributes a relatively large share of the ocean carbon sink, with CO2 uptake rates higher than those of the open ocean[5-11]. The total ocean currently absorbs approximately 2.4 ± 0.5 Pg C yr-1 of atmospheric CO2[12]. By

comparison, the coastal ocean, defined here as the continental shelf sea, is estimated to absorb 0.18–0.45 Pg C yr-1 [7,8,11,13].'

I find this introduction still rather misleading.

Ref 12 is a rather old estimate of the global ocean carbon sink from Le Quéré et al., 2018, i.e. the Global Carbon Budget of 2017. More recently, Friedlingstein et al., 2025 reported a stronger ocean carbon sink of 2.9 ± 0.4 Pg C yr-1 (GCB24; decadal average from 2014 to 2023; their Table 7), and very recently this number was further corrected upwards to 3.1 ± 0.5 Pg C uptake per year (Friedlingstein et al., 2026; their Table 1).

Dai et al., 2022 (ref 7) report a global coastal CO2 uptake of 0.25 ± 0.5 Pg C yr-1. Laruelle et al., 2014 (ref 8) report a global coastal CO2 uptake of 0.19 ± 0.05 Pg C yr-1 (the upper estimate of 0.45 PgC yr-1 is not reported by this study, please remove this in L37). Roobaert et al., 2019 (ref 13) and 2024 (ref 11) report a global coastal CO2 uptake of 0.2 ± 0.02 and 0.18 ± 0.01 Pg C yr-1 (their 'narrow coastal ocean'), respectively.

Putting these reported global integrals ± uncertainties of the coastal versus total ocean carbon sink into perspective, I obtain (coastal/GCB*100):

dai_etal_2022_ref7 vs gcb17: -8.6 to 39 %

dai_etal_2022_ref7 vs gcb24: -7.6 to 30 %

dai_etal_2022_ref7 vs friedlingstein_etal_2026: -6.9 to 29 %

laruelle_etal_2014_ref8 vs gcb17: 4.8 to 13 %

laruelle_etal_2014_ref8 vs gcb24: 4.2 to 9.6 %

laruelle_etal_2014_ref8 vs friedlingstein_etal_2026: 3.9 to 9.2 %

roobaert_etal_2019_ref_13 vs gcb17: 6.2 to 12 %

roobaert_etal_2019_ref_13 vs gcb24: 5.5 to 8.8 %

roobaert_etal_2019_ref_13 vs friedlingstein_etal_2026: 5 to 8.5 %

roobaert_etal_2024_ref11_narrow vs gcb17: 5.9 to 10 %

roobaert_etal_2024_ref11_narrow vs gcb24: 5.2 to 7.6 %

roobaert_etal_2024_ref11_narrow vs friedlingstein_etal_2026: 4.7 to 7.3 %

roobaert_etal_2024_ref11_wide vs gcb17: 13 to 23 %

roobaert_etal_2024_ref11_wide vs gcb24: 11 to 17 %

roobaert_etal_2024_ref11_wide vs friedlingstein_etal_2026: 10 to 17 %

Hence, based on these shares of 3.9 to 9.2% versus the corrected GCB24 (Friedlingstein et al., 2026), I actually don't think that the coastal ocean 'contributes a relatively large share of the ocean carbon sink', as their ~7% area share of the global ocean lies well within this range (here excluding both the Dai et al., 2022 estimate due to its large uncertainty as well as the upper 'wide coastal ocean' estimate of 0.4 ± 0.03 Pg C yr-1 of Roobaert et al., 2024 of 10 to 17%, as the area used for this estimate is larger than the area used in the other studies). Please rephrase.

Response: We appreciate the reviewer for pointing out that our original wording and supporting references were not sufficiently accurate.

First, regarding the estimate of the global ocean carbon sink, we agree that the reference to the Global Carbon Budget 2017 is no longer the most appropriate. Accordingly, we have updated the estimate to the latest value reported by Friedlingstein et al.[1], namely 3.1 ± 0.5 Pg C yr^{-1}.

Second, we thank the reviewer for identifying the inconsistency in the reported range of the global coastal CO_2 sink. We have corrected the estimate from 0.18–0.45 Pg C yr^{-1} to 0.18–0.25 Pg C yr^{-1}.

Finally, we agree that the phrase "contributes a relatively large share of the ocean carbon sink" was not appropriate. As the reviewer noted, recent estimates indicate that the coastal ocean contributes approximately 5–8% of the global ocean carbon sink, broadly comparable to its ~7% share of the global ocean surface area. Accordingly, we have removed both the statement that "it contributes a relatively large share of the ocean carbon sink" and the accompanying statement that "with CO_2 uptake rates higher than those of the open ocean".

The revised text now reads (Page 2, Lines 33–35): "*The global ocean currently absorbs approximately 3.1 ± 0.5 Pg C yr-1 of atmospheric CO_2[5]. By comparison, the coastal ocean, defined here as the continental shelf sea, is estimated to absorb 0.18–0.25 Pg C yr^{-1} [6-9].*"

L55: 'In coastal waters, where biological productivity and human activity are concentrated, MHWs exhibit stronger spatial heterogeneity than in the open ocean and can exert disproportionately large impacts[28,34,42,44].'

I don't see that any of these refs (ref 28: Edwing et al., 2024, ref 34: Marin et al., 2021, ref 42: Cheung and Frölicher., 2020, ref 44: Arias-Ortiz et al., 2018) show that coastal MHWs exhibit a 'stronger spatial heterogeneity than in the open ocean'. Please provide references which show this or drop this part of the sentence.

Response: We sincerely thank the reviewer for this helpful comment. We agree that the original wording was not sufficiently accurate, as the cited references did not directly support the comparison that coastal MHWs exhibit stronger spatial heterogeneity than those in the open ocean.

Accordingly, we have removed the phrase "stronger spatial heterogeneity than in the open ocean" and revised it to "*MHWs exhibit strong spatial heterogeneity*" (Page 3, Line 54). This statement is supported by Marin et al.[2], who showed that the frequency, duration, and mean intensity of coastal MHWs exhibit strong spatial heterogeneity on a global scale.

In addition, we have separated the citations to better align each statement with its supporting evidence. Specifically, Marin et al.[2] is cited to support the statement on the spatial heterogeneity of coastal MHWs, whereas Edwing et al.[3], Cheung et al.[4], and Arias-Ortiz et al.[5] are cited to support the statement that coastal MHWs can exert large impacts on the coastal carbon sink, fisheries, and seagrass carbon stocks, respectively.

L100: Please add the meaning of positive/negative FCO2 to the caption of Supplementary Fig. 2.

Response: We have added the meaning of positive and negative FCO_2 to the caption of Supplementary Fig. 2 as follows (Page 3, Lines 33–35 in Supplementary Information): "*In panels (**a**) and (**b**), positive (negative) FCO_2 values indicate CO_2 outgassing from the ocean to the atmosphere (CO_2 uptake by the ocean).*"

L105: Please add the meaning of positive/negative FCO2 to the caption of Supplementary Fig. 3.

Response: We have clarified the meaning of positive and negative FCO_2 anomalies in the caption of Supplementary Fig. 3 as follows (Page 4, Lines 43–44 in Supplementary Information): "*Positive anomalies indicate enhanced CO_2 outgassing or reduced uptake, whereas negative anomalies indicate enhanced uptake or reduced outgassing.*"

L114: Please add some words what 'anomaly' here means, i.e. with respect to what, since it's the first occurrence of 'anomaly' in the main text.

Response: We have added (Page 5, Lines 112–113) "*relative to the trend-preserving climatology (see Methods)*" to clarify the reference state of the anomaly, as suggested by the reviewer.

L117: 'carbon units (Tg)' → 'Tg C' or 'mass units'?

Response: Revised to "*Tg C*" (Page 5, Line 116) as suggested.

L120: '11%'

This number changed substantially from the 1st version of the manuscript where it has been only a 4.3% increase of global coastal CO2 uptake during MHW months (with respect to MHW-months and MHW-grid points only). I assume the difference originates from detrending the data prior to extreme event detection (done in 2nd version but not in 1st version). I am somewhat puzzled by this. Can you please add this piece of information somewhere to the manuscript (e.g. the Methods section) and speculate about its meaning? Thanks!

Response: We thank the reviewer for pointing this out. The difference between the two estimates mainly results from detrending the SST data prior to MHW detection in the revised analysis.

Without detrending, MHW detection is more likely to identify months in the warmer (more recent) part of the time series as MHW events. Consequently, the detected MHWs may reflect a mixture of long-term climate warming and short-term extreme warming events. In the first version of the manuscript, however, FCO_2 anomalies were calculated relative to the trend-preserving climatology, which accounts for the long-term trend in the background state. Therefore, the inconsistency between the MHW detection approach (without SST detrending) and the FCO_2

anomaly calculation (relative to the trend-preserving climatology) resulted in the inclusion of relatively weak MHW events that would not have been identified after SST detrending and that were associated with weaker FCO_2 anomalies. This likely contributed to the lower estimated enhancement of coastal CO_2 uptake (4.3%) in the previous version.

In this study, our primary objective is to investigate the anomalous air–sea CO_2 flux response to MHWs arising from internal climate variability rather than the "total heat exposure", which combines the effects of long-term warming and MHWs, as described by Amaya et al.[6].

We have therefore added an explanation in the Methods section to clarify why SST was detrended prior to MHW detection, as follows (Pages 13–14, Lines 364–369): "*MHWs can be defined either relative to the historical climatology or after removing the long-term warming trend to isolate short-term variability, depending on the research objective[93]. In this study, our primary objective is to investigate the anomalous air–sea CO_2 flux response to MHWs arising from internal climate variability. Therefore, SST data from 1982 to 2020 were detrended prior to MHW detection to remove the long-term warming trend, after which MHWs were identified for the period 1985–2020.*"

L120: '… 11% increase … during 1985-2020' to me sounds like a temporal change or trend. Is it possible to rephrase so that it becomes clear that the coastal FCO2 uptake during MHW months is stronger/larger compared the climatology or such?

Response: The sentence has been revised as follows (Page 5, Lines 118–119): "*Over the period 1985–2020, the integrated FCO_2 anomaly is –40.02 Tg C, corresponding to an 11.0 ± 1.6% enhancement in global coastal CO_2 uptake.*", (Page 5, Lines 124–126) "*The reported percentage anomalies are calculated by normalizing the integrated anomalies against the corresponding integrated climatological fluxes only for MHW-affected coastal areas and MHW months, following the methodology of Mignot et al.[47] and Li et al.[46].*"

L126: What exactly is the 'climatological sum'? And more general: I am not sure that I fully understand the difference between the relative MHW effect of 11% with respect to the 'climatological sums only for MHW-affected coastal areas and MHW months' (L126-127) versus

the much smaller 0.62% with respect to the 'cumulative CO2 uptake of the global coastal ocean over all months' (L128-129). Is there a difference between 'sum' and 'cumulative'? Actually, which of these numbers is the more interesting one?

Also: Which of these two numbers can be put into perspective with the relative effects of MHWs in the open ocean reported by Mignot et al., 2022 (ref 52) and Li et al., 2024 (ref 51; e.g., 8% CO2 uptake reduction globally, 31% or 40% CO2 release reduction in equatorial Pacific, 29% CO2 uptake reduction in North Pacific)? I assume your reported MHW-month- and MHW-grid-wise relative contribution of 11%. But I am not sure; did Mignot et al. and Li et al. derive their relative contributions similarly MHW-time- and MHW-grid-wise? I find their (and your) methods sections not very precise. Thanks for clarification.

Response: We agree that the term "climatological sums" was not sufficiently precise. What we intended to refer to was "corresponding integrated climatological fluxes". Accordingly, we have revised the sentence as follows (Page 5, Lines 124–126): "*The reported percentage anomalies are calculated by normalizing the integrated anomalies against the corresponding integrated climatological fluxes only for MHW-affected coastal areas and MHW months, following the methodology of Mignot et al.*[47] *and Li et al.*[46]."

The 11% value quantifies the intensity of the MHW response within MHW-affected regions and months, whereas the 0.62% value quantifies the contribution of this integrated anomaly to the cumulative coastal CO_2 uptake over the entire coastal ocean and the entire study period.

We think that 11% is the more informative metric. Accordingly, it is highlighted in the Abstract, as it is directly comparable to the relative effects of MHWs reported for the open ocean by Mignot et al.[7] and Li et al.[8]. By contrast, the 0.62% value is provided only to place the integrated anomaly in the context of the overall coastal CO_2 uptake.

Our reported 11% enhancement is calculated using the same MHW-month- and MHW-grid-based normalization as adopted by Mignot et al.[7] and Li et al.[8]. Specifically, Mignot et al.[7] state in their Methods that "The percent F_{CO2} anomalies during PMHWs and for the 1985–2017 period correspond to the monthly F_{CO2} anomalies divided by the **monthly** F_{CO2} climatological values." Likewise, Li et al.[8] state that "Subsequently, the anomalies during MHWs are derived as deviations from the climatological seasonal cycle of monthly-mean CO_2 fluxes **during MHWs**."

L130: Ref 59 is Le Quéré et al., 2014, even older then Le Quéré et al., 2017 (ref 12) that you have used in the introduction. Why are you citing these old numbers?

Response: We selected this old estimate because we aimed to provide an order-of-magnitude comparison using a global ocean CO_2 uptake value that better matches our study period (1985–2020). We clarify that this comparison is only a rough estimate.

L137: I think Mignot et al., 2022 (ref 52) should be cited here as well.

Response: Cited as suggested.

L147: 'both integrated and area-weighted' I assume you are referring → spatial integral and spatial mean. Both are 'area-weighted' by definition. Please clarify.

Response: We thank the reviewer for pointing this out. The phrase "both integrated and area-weighted mean FCO_2 anomalies" has been revised to (Page 6, Lines 146–147) "*both spatial integrals and spatial means of FCO_2 anomalies*" for clarity.

L149: Please add the meaning of positive/negative FCO2 to the caption of Supplementary Fig. 6.

Response: We have added the explanation to the caption of Supplementary Fig. 6 as follows (Page 7, Lines 68–70 in Supplementary Information): "*Positive anomalies indicate contributions from enhanced CO_2 outgassing or reduced uptake, whereas negative anomalies indicate contributions from enhanced uptake or reduced outgassing.*"

L152: 'In tropical regions, MHWs amplify coastal CO2 outgassing but suppress open ocean outgassing[51,52,63] (Supplementary Fig. 3), despite both being climatological sources of atmospheric CO2. At higher latitudes, polar and subpolar coastal shelf seas exhibit enhanced CO2 uptake during MHW months, while uptake in the open North Pacific weakens[51,52] (Supplementary Fig. 3),

although both are climatological CO2 sinks. These divergent FCO2 anomalies show that coastal and open-ocean systems with similar climatological roles can respond in opposite ways to MHWs.'

Thanks a lot for Supplementary Fig. 3. I think it would help to 1) show both open and coastal ocean CO2 uptake anomalies in one plot (I did so in Review Fig. 1 below) and 2) include only those grid points in the zonal mean that exhibit statistically significant FCO2 anomalies in open and coastal oceans (the latter according to Supplementary Fig. 2d).

To me, the open and coastal oceans react very similar during MHW months, except in the tropics, where the open ocean shows a substantially increased uptake and/or reduced outgassing, while the tropical coasts seem to show the opposite, although much of these zonally averaged anomalies are – I speculate - likely not significant. Hence, in my view, the only regions where 'divergent FCO2 anomalies' occur (if at all: significance) are the tropics, and not the high latitudes. Please rephrase this paragraph accordingly.

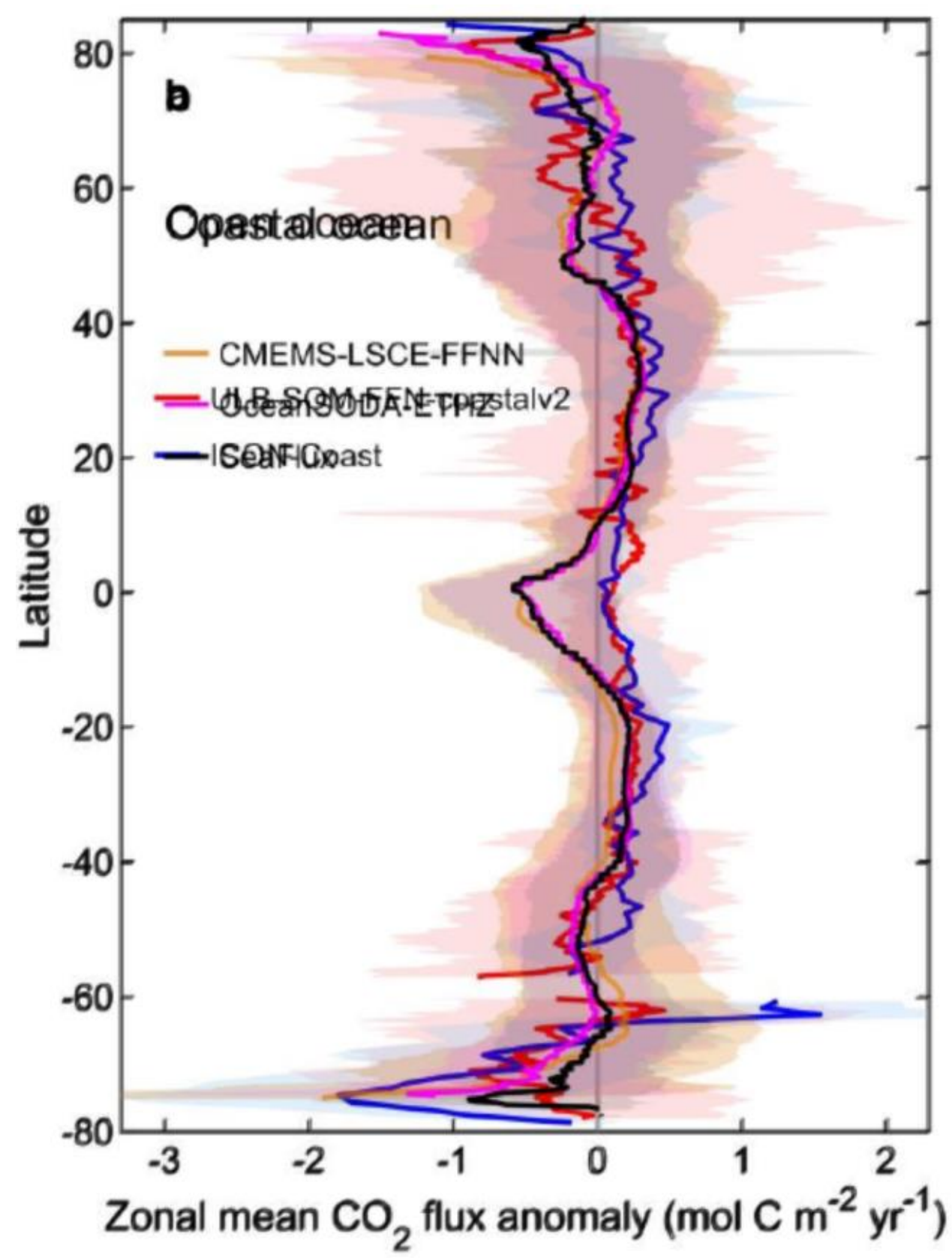

Review Fig. 1: Overlay of Supplementary Fig. 3a and b from 2nd manuscript version. Open (black) and coastal (red) oceans exhibit very similar CO2 uptake anomalies during MHW months, except in the tropics (there, however, the coastal anomalies are very small and likely not significant).

Response: We thank the reviewer for this helpful suggestion. We have now shown the open and coastal ocean CO_2 uptake anomalies in the same plot and included only grid cells with statistically significant FCO_2 anomalies in the zonal-mean calculation.

“

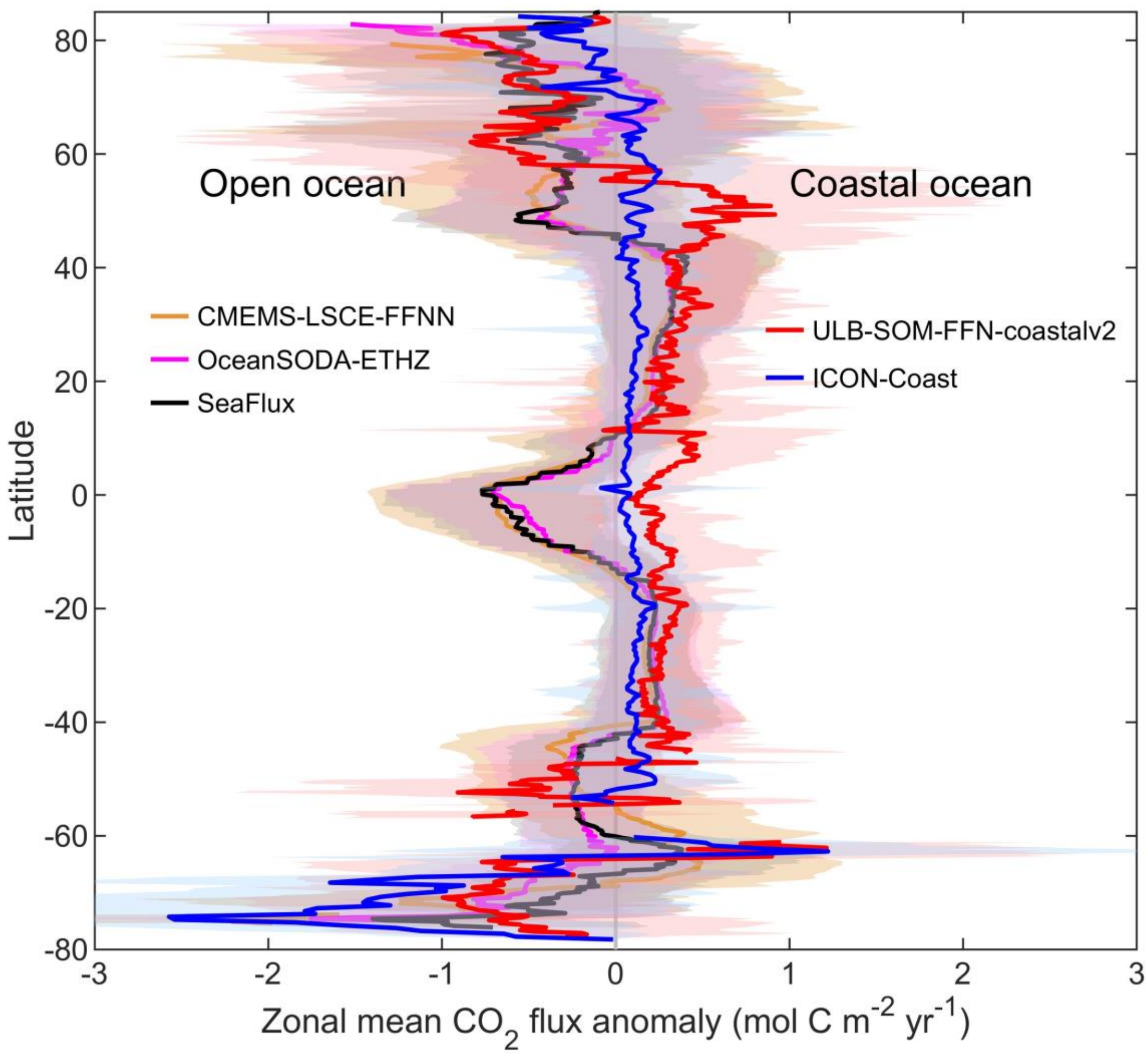

Supplementary Fig. 3 | Zonal mean CO_2 flux anomalies during MHW months (1985–2020) in the open ocean and coastal ocean. *Zonal mean CO_2 flux anomalies are shown for the open ocean (derived from CMEMS-LSCE-FFNN, OceanSODA-ETHZ, and SeaFlux) and the coastal ocean (derived from ULB–SOM–FFN–coastalv2 and ICON-Coast). Only grid cells exhibiting statistically significant FCO_2 anomalies are included in the zonal-mean calculation for both ocean domains. Positive anomalies indicate enhanced CO_2 outgassing or reduced uptake, whereas*

negative anomalies indicate enhanced uptake or reduced outgassing. Shading indicates ±1 standard deviation of the anomalies."

We agree that the zonal-mean responses of the coastal and open ocean are broadly similar at high latitudes. Our original intention, however, was not to compare the responses of coastal and open-ocean regions within the same latitude bands, but rather to identify the regions that contribute most strongly to the integrated responses of the coastal and open oceans, respectively.

We have therefore revised the text accordingly. The revised sentence now reads (Page 6, Lines 153–162): "*At higher latitudes, polar and subpolar coastal shelf seas exhibit enhanced CO_2 uptake during MHW months, similar to the open ocean at the same latitudes (Supplementary Fig. 3). In terms of the integrated response, however, the polar and subpolar coastal shelf seas contribute most strongly to the coastal response (Fig. 1b), whereas the open North Pacific contributes most strongly to the open-ocean response[46,47]. Although both are climatological CO_2 sinks, MHWs enhance CO_2 uptake in the former (Fig. 1b) but weaken CO_2 uptake in the latter[46,47] (Supplementary Fig. 3). These results indicate that coastal and open-ocean systems with similar dominant regional contributions and climatological roles can respond differently to MHWs. Understanding the mechanisms underlying these differences is therefore essential for assessing the contribution of coastal oceans to the global carbon budget during ongoing MHWs.*"

L162ff: In the whole paragraph, please formulate more explicitly how the important FCO2 anomaly drivers change the ocean CO2 uptake during MHW months; do they weaken or strengthen the uptake? I find formulations like 'enhance fluxes' very unclear. For this, please add the meaning of positive/negative FCO2 contributions to the caption of Supplementary Fig. 7.

Response: We agree that the term "enhance fluxes" was not sufficiently clear. We have revised the text from "The thermal contribution of $pCO_{2,sea}$ consistently enhances fluxes" to (Page 7, Line 173) "*The thermal contribution of $pCO_{2,sea}$ consistently enhances CO_2 outgassing*".

In addition, we have clarified the meaning of positive and negative FCO_2 anomaly contributions in the caption of Supplementary Fig. 7 as follows (Page 8, Lines 76–78 in Supplementary Information): "*Positive anomalies indicate contributions from enhanced CO_2 outgassing or*

reduced uptake, whereas negative anomalies indicate contributions from enhanced uptake or reduced outgassing."

L187: '… reinforcing that oceanic processes dominate coastal FCO2 anomalies during MHW months.'

In my view '… as in the global ocean.[51,52]' or such could be added at the end of the sentence (ref 51: Li et al., 2024; ref 52: Mignot et al., 2022).

Response: We thank the reviewer for this suggestion. In most of the open ocean, contributions from wind, residual terms, and $pCO_{2,air}$ are indeed negligible (their Figure 2a in Li et al.[8]). However, there are some exceptions where wind contributions are not negligible, such as the subpolar North Atlantic (their Figure 2c) and the equatorial Pacific (their Figure 2f).

We have added "*as also reported for most of the open ocean*[46,47]" (Page 7, Line 191) at the end of the sentence.

L189: 'In open oceans, the response of the oceanic carbon sink to anomalous warming is mainly governed by the balance of thermal and non-thermal drivers[51,52,58]'.

In this general sense this is also true for the coastal oceans (Fig. 2, Supplementary Fig. 6c). Please rephrase.

Response: We agree that this is also true for the coastal ocean. We have therefore added "also" to the sentence. The revised sentence now reads (Page 7, Lines 192–193): "*In open oceans, the response of the oceanic carbon sink to anomalous warming is also mainly governed by the balance of thermal and non-thermal drivers*[46,47,53]*.*"

L204 & Fig. 3: Why these non-intuitive units of sea ice fraction anomalies during MHW months? For FCO2, you took the temporal average of anomalies. Why taking the temporal sum for sea ice? Wouldn't it be more informative to know the average sea ice fraction (=percent) anomaly during MHW months?

Response: For FCO$_2$ anomaly, we present the temporal mean anomalies in Fig. 1a, whereas the dominant role of the polar and subpolar shelf seas is identified from the integrated FCO$_2$ anomalies in Fig. 1b. Figs 2–4 are intended to explain why the polar and subpolar regions dominate the integrated response.

In Fig. 3, our focus is on the contribution of sea ice reduction to the integrated FCO$_2$ anomaly. Therefore, we show the spatial distribution of the integrated sea ice fraction anomaly, which accounts for both the magnitude of the anomaly and the frequency of MHW months. The average contribution of sea ice reduction to the FCO$_2$ anomaly is presented in Supplementary Fig. 7a.

L222: Some bars in Fig. 4e-h and Supplementary Fig. 9e-h seem to differ quite substantially between this 2nd version of the manuscript and the 1st version (Fig. 4e-h and Extended Data Fig. 8e-h therein). Why?

Thanks for explaining the budget terms in the caption of Fig. 4e-h. However, I am still confused by the meaning of 'import' and 'export' of the Δadv imp IC and Δadv exp OC terms. Is a positive Δadv exp OC term an OC gain or a loss? Thanks for clarification. I think it makes sense to shorten the now rather long caption of Fig. 4 by simply moving the budget explanation to the Methods section 'Model evaluation' or such.

Response: In version 1, we identified MHW months and regions from the observational SST dataset and applied these to extract the corresponding CO_2 fluxes from the model output, aiming to remain as consistent with the observations as possible. However, following the reviewers' suggestions, in version 2 MHWs were identified directly from the daily model SST output. In addition, SST was detrended prior to MHW detection. These methodological changes altered the identified model MHW months and regions, leading to the differences in the budget terms shown in Fig. 4e–h and Supplementary Fig. 9e–h.

The names of the advective budget terms are related to the mean states of the advective fluxes, respectively. The coastal regions considered in our analysis typically act as autotrophic regions with a net transformation of inorganic carbon into organic matter. Hence, regarding advective transports, the mean state is a net import of IC into the region and a net export of OC out of the region. We found that during MHWs the advective export of OC is reduced, which means that for

the budget Δadv exp OC results in an OC gain, retaining more OC in the region (also indicated by an increase in OC stock).

We have moved the explanation of the budget terms to the "Model evaluation" subsection of the Methods section, as suggested.

L244: '… consistent with the observed negative DIC anomalies.'

Where do I see the negative DIC anomalies? Δstock IC in Fig. 4f-h is positive in three out of four segments. It becomes somewhat clearer with the next sentence but I find the current formulation confusing. Actually, the model is reproducing the effect of MHWs on the coastal FCO2 (Supplementary Fig. 3b, Supplementary Fig. 14), but for the wrong reason (not the DIC reduction)? I think it's important to clarify this as this is the last key point in the abstract.

Response: The upper ocean carbon budgets represent the net impacts of different carbon fluxes and transformation processes in the top 50 m, providing a more comprehensive picture about the net biological response in the upper water column. Corresponding changes in the DIC stock are thus not directly comparable to anomalies in surface DIC concentrations. The former also account for vertical gradients, and changes in vertically integrated DIC stock do not translate directly into changes in surface DIC concentration because they depend on the pre-existing vertical distribution of DIC.

We have revised this paragraph and added a supplementary table summarizing the simulated model anomalies in surface DIC and non-thermal $p\mathrm{CO_2}$, showing their consistence with observations.

(Page 9, Lines 244–254) "*Upper ocean carbon budgets derived from model results suggest that polar and subpolar shelf seas consistently respond to MHW months with marked increases in NPP (Fig. 4e–h). These productivity enhancements are expected to reduce surface DIC and pCO$_2$, thereby favoring enhanced atmospheric CO$_2$ uptake. While simulated surface DIC anomaly is largely consistent with the observed negative DIC anomalies (Supplementary Table 2), changes in the carbon budget terms provide a more comprehensive picture about the net biological response in the upper water column. Anomalies in inorganic carbon (IC) transport and organic matter decomposition outweigh the effects of enhanced biological carbon fixation, resulting in net positive DIC accumulation in several high-latitude regions despite lower DIC concentrations at the surface.*

Yet, the model does not fully reproduce the observed CO_2 uptake response, probably due to biases in the individual physical and biogeochemical CO_2 flux contributions."

"***Supplementary Table 2 | Simulated anomalies in surface DIC and non-thermal $pCO_{2,sea}$ during MHW months for the representative MARCATS segments.***

MARCATS	Surface DIC anomalies (mg C m^{-3})	Non-thermal $pCO_{2,sea}$ anomalies (µatm)
**10**	–9.1	–4.9
**37**	+1.7	–0.5
**38**	–7.0	–0.9
**39**	–17.4	–3.2
**42**	–24.0	–5.2
**43**	–37.0	–6.3
**44**	–3.2	–2.8
**45**	–37.4	–6.5

Values represent the mean anomalies averaged over all MHW months in each MARCATS segment."

L269: 'While NPP gains are largely offset by enhanced carbon transport and organic carbon (OC) decomposition (Fig. 4e–h), in regions such as the North-western Pacific shelves, NPP increases substantially surpass concurrent increases in OC decomposition ...'

I don't see how segment 42 (Fig. 4e) exhibits 'substantially' different dynamics compared to segments 43-45 (Fig. 4f-h). Please clarify.

Response: We agree that the term "substantially" was too strong and have revised the statement as follows (Page 10, Lines 275–279): "*While NPP gains are largely offset by enhanced carbon transport and organic carbon (OC) decomposition in the upper water column (Fig. 4e–h), in regions such as the North-western Pacific shelves, NPP increases surpass concurrent increases in OC decomposition, yielding a net positive shift in ecosystem production. This strong biological*

control contrasts sharply with the mechanism in the open ocean, where negative DIC anomalies primarily arise from horizontal advection[47]."

L359: 'influence' of what?

Response: We have clarified that the SST data were detrended to remove the long-term warming trend. The revised sentence now reads (Page 14, Lines 367–368): "*SST data from 1982 to 2020 were detrended prior to MHW detection to remove the long-term warming trend*".

L438 and L441: If you add 'mol C' to the units FCO2, then that should be added to the units of K0 as well?

Response: We thank the reviewer for this comment. Although FCO_2 is expressed in units of mol C m^{-2} yr^{-1} because it represents the flux of carbon, the solubility coefficient (K_0) is conventionally expressed in units of mol m^{-3} μatm^{-1} [9,10] because it describes the solubility of CO_2 in seawater, where mol refers to the amount of CO_2 rather than carbon. We have therefore retained the conventional unit for K_0.

L439: Why 'net' uptake?

Response: We thank the reviewer for pointing this out. We have removed "net". The sentence now reads (Page 16, Lines 447–448): "*Positive values indicate CO_2 outgassing from the ocean, whereas negative values indicate CO_2 uptake by the ocean.*"

L447: 'For each variable and grid cell, a trend-preserving climatology was first calculated over the full 1985–2020 time series, combining a long-term linear trend with a mean seasonal cycle derived from detrended data[52,58]. Anomalies during MHW months were then defined as the deviation of each MHW months from its corresponding climatological value.'

I don't fully understand how you calculated the climatology. I understand that for every variable and grid cell you calculated:

trend(t_i) = calc_linear_trend(data(t_i)) 1

detrended_data(t_i) = data(t_i) – trend(t_i) 2

monthly_clim_of_detrended_data(mon_j) = calc_monthly_clim(detrended_data(t_i)) 3

monthly_clim(mon_j) = trend(t_i) + monthly_clim_of_detrended_data(mon_j) 4

monthly_anomaly(mhw_mon_k) = data(mhw_mon_k) - monthly_clim(mon_j) 5

for i = 1,…,432 (= 36 years from 1985 to 2020, times 12), j = 1,…,12, and k = 1,…,n with n the total number of identified MHW months. In steps 4 and 5, the corresponding climatological month j (mon_j) was chosen for each time point t_i and MHW month mhw_mon_k, respectively. Is that correct? It's ok if you want to avoid equations like this but I would appreciate to reformulate so that it becomes a bit clearer. Thanks!

Also, why are you both detrending the data and then adding the trend again? Why not simply calculating the monthly climatology alone? I would ask the the same question to the two given refs as I don't understand why they did this as well (ref 52: Mignot et al., 2022, ref 58: Müller et al., 2025). Thanks for clarification.

Response: We thank the reviewer for the helpful suggestion. The workflow described by the reviewer correctly represents our calculation procedure. We have revised the Methods section to make this procedure clearer as follows (Page 17, Lines 456–463): "*Monthly anomalies were calculated to isolate the short-term impacts of MHWs while removing the influence of long-term changes associated with global warming and rising atmospheric CO_2[99]. For each variable and grid cell, a trend-preserving climatology was calculated over the 1985–2020 period[47,53]. The long-term linear trend was first removed from the time series, and the monthly climatology was then calculated from the detrended data to isolate the mean seasonal cycle. The linear trend was subsequently added back to the seasonal cycle to obtain a trend-preserving climatology. Anomalies during MHW months were then defined as the deviations from the corresponding climatological values.*"

The ocean carbon sink has increased since 1990[11]. In our study, the climatology retains the long-term increasing trend in ocean CO_2 uptake, and therefore the calculated anomalies remove the influence of this trend. If we simply calculated a monthly climatology without preserving the trend, the resulting FCO_2 anomalies would include this background increase in CO_2 uptake. Therefore,

following the approach adopted by Mignot et al.[7] and Müller et al.[12], our method also isolates the short-term impacts of MHWs relative to the expected background state.

L470: ‘The anomalies a’, b’, c’ are defined relative to their climatological states ($\bar{a}, \bar{b}, \bar{c}$), which include the long-term trend.’

Your review response to eq. 3: ‘In our formulation, the overbar denotes the climatological state including the long-term trend rather than a simple temporal mean. Therefore, $\overline{\bar{a}\bar{b}\bar{c}}$ is not equal to $\bar{a}\bar{b}\bar{c}$.’

I am confused. To me, only one temporal mean is defined in your equations 2-4: the overbar. Hence, $\overline{\bar{a}\bar{b}\bar{c}} = \bar{a}\bar{b}\bar{c}$ and the zero-order terms in eq. 3 vanish. Let e.g. a = (1 2), b = (1 2) and c = (1 2), i.e. all vectors representing data from 2 time points at some location:

$$\overline{\overline{(1\ 2)(1\ 2)(1\ 2)}} = \overline{1.5 * 1.5 * 1.5} = \overline{3.375} = 3.375$$

Couldrey et al., 2016, the study that you are following in this methodology (ref 104), conclude the same on their page 790. Please clarify if you are using different temporal means so that these zero-order terms do not vanish.

Response: In our formulation, the overbar does not denote a temporal mean. Instead, it denotes the trend-preserving climatological time series corresponding to each month. Therefore, $\bar{a}$, $\bar{b}$, $\bar{c}$ are time-varying climatological series rather than constants. Consequently, $\overline{\bar{a}\bar{b}\bar{c}}$ represents the trend-preserving climatology of $\bar{a}\bar{b}\bar{c}$, rather than a constant temporal mean of $\bar{a}\bar{b}\bar{c}$.

Although our decomposition follows the framework of Couldrey et al.[13], our definition of the climatological state differs because we adopt a trend-preserving climatology.

We clarified this explicitly in the Methods section by stating (Page 17, Lines 464–466): “*Consistent with this approach, all climatological fields in this study, denoted with an overbar, retained the long-term trend to ensure methodological consistency.*”, (Page 18, Lines 483–484) “*The anomalies a', b', c' are defined relative to their climatological states ($\bar{a}, \bar{b}, \bar{c}$), which include the long-term trend.*”

L512: ‘(12-16 and 41-45)’ ‘(segments → 12-16 and 41-45)’

Response: We thank the reviewer for the suggestion. We have clarified this in the manuscript by replacing “12–16 and 41–45” with “*MARCATS 12–16 and 41–45*” (Page 19, Line 526).

References

Arias-Ortiz et al., 2018: https://www.nature.com/articles/s41558-018-0096-y

Cheung and Frölicher, 2020: https://www.nature.com/articles/s41598-020-63650-z

Couldrey et al., 2016: https://agupubs.onlinelibrary.wiley.com/doi/10.1002/2015GB005267

Dai et al., 2022: https://www.annualreviews.org/content/journals/10.1146/annurev-earth-032320-090746

Edwing et al., 2024: https://agupubs.onlinelibrary.wiley.com/doi/10.1029/2023GL105363

Friedlingstein et al., 2025: https://essd.copernicus.org/articles/17/965/2025/

Friedlingstein et al., 2026: https://www.nature.com/articles/s41586-025-09802-5

Laruelle et al., 2014: https://agupubs.onlinelibrary.wiley.com/doi/10.1002/2014GB004832

Le Quéré et al., 2014: https://essd.copernicus.org/articles/6/235/2014/essd-6-235-2014.html

Le Quéré et al., 2018: https://essd.copernicus.org/articles/10/2141/2018/

Li et al., 2024: https://agupubs.onlinelibrary.wiley.com/doi/10.1029/2024GL110379

Marin et al., 2021: https://agupubs.onlinelibrary.wiley.com/doi/10.1029/2020JC016708

Mignot et al., 2022: https://www.nature.com/articles/s41467-022-31983-0

Müller et al., 2025: https://www.nature.com/articles/s41558-025-02380-4

Roobaert et al., 2019: https://agupubs.onlinelibrary.wiley.com/doi/10.1029/2019GB006239

Roobaert et al., 2024: https://essd.copernicus.org/articles/16/421/2024/

References for Responses to Reviewer #3

1. Friedlingstein, P. et al. Emerging climate impact on carbon sinks in a consolidated carbon budget. *Nature* **649**, 98-103 (2026).
2. Marin, M., Feng, M., Phillips, H. E. & Bindoff, N. L. A global, multiproduct analysis of coastal marine heatwaves: distribution, characteristics, and long-term trends. *J. Geophys. Res. Oceans* **126**, e2020JC016708 (2021).
3. Edwing, K. et al. Impact of marine heatwaves on air-sea CO_2 flux along the US East Coast. *Geophys. Res. Lett.* **51**, e2023GL105363 (2024).
4. Cheung, W. W. L. & Frolicher, T. L. Marine heatwaves exacerbate climate change impacts for fisheries in the northeast Pacific. *Sci Rep* **10**, 6678 (2020).
5. Arias-Ortiz, A. et al. A marine heatwave drives massive losses from the world's largest seagrass carbon stocks. *Nat. Clim. Change* **8**, 338–344 (2018).
6. Amaya, D. J. et al. Marine heatwaves need clear definitions so coastal communities can adapt. *Nature* **616**, 29–32 (2023).
7. Mignot, A. et al. Decrease in air-sea CO_2 fluxes caused by persistent marine heatwaves. *Nat. Commun.* **13**, 4300 (2022).
8. Li, C., Burger, F. A., Raible, C. C. & Frölicher, T. L. Observed regional impacts of marine heatwaves on sea-air CO_2 exchange. *Geophys. Res. Lett.* **51**, e2024GL110379 (2024).
9. Wanninkhof, R. Relationship between wind speed and gas exchange over the ocean. *J. Geophys. Res. Oceans* **97**, 7373-7382 (1992).
10. Weiss, R. F. Carbon dioxide in water and seawater: the solubility of a non-ideal gas. *Mar. Chem.* **2**, 203–215 (1974).
11. Gruber, N. et al. Trends and variability in the ocean carbon sink. *Nature Reviews Earth & Environment* **4**, 119-134 (2023).
12. Müller, J. D. et al. Unexpected decline in the ocean carbon sink under record-high sea surface temperatures in 2023. *Nat. Clim. Change* **15**, 978–985 (2025).
13. Couldrey, M. P., Oliver, K. I. C., Yool, A., Halloran, P. R. & Achterberg, E. P. On which timescales do gas transfer velocities control North Atlantic CO_2 flux variability? *Glob. Biogeochem. Cycles* **30**, 787–802 (2016).

Response to the reviewer's comments (third round)

We sincerely thank the reviewer for the additional comments. We appreciate the opportunity to clarify key points and further improve the manuscript, and we have carefully addressed all remaining comments through additional revisions. Detailed point-by-point responses to each comment (shown in black) are provided below. Our responses are presented in blue, and the corresponding revisions in the manuscript are highlighted in *italic* and <u>underlined</u> text.

Reviewer #3 (Remarks to the Author):

Review 3 of Nature Communications ID NCOMMS-25-92847-T: 'Polar and subpolar shelf seas dominate enhanced coastal CO_2 uptake during marine heatwaves' by Zhang et al.

Table of Contents

Summary...1
Minor...1
References...4

Summary

Thank you Zhang et al. for working on all comments of the 2nd review. I am especially thankful for for Supplementary Fig. 3 and Supplementary Table 2.

Based on your revisions, there are some final minor comments from my side. The last of those, L474, is somewhat important.

Response: We thank the reviewer for the helpful comments and suggestions. We have carefully addressed all of the remaining comments, including the important comments regarding the climatology calculation at L458 and L474.

Minor

L57: 'In the Arctic, long-term warming and declining sea ice act to amplify MHW intensity[46,47], ...'

Table S3 of Huang et al., 2021 (ref 46) shows that Arctic MHW intensity (their 'SSTA') is not significantly correlated with sea ice concentration.

He et al., 2024 (ref 47) excluded all grid points where the sea ice concentration is >35%, which are quite a lot (their Supplementary Fig. 4). Hence, I cannot rule out that the latitudinal differences in MHW and THW (i.e. detrending or not) intensity changes from 1985-2014 to 2071-2100 shown in their Fig. 2c and g (what they termed 'Arctic Amplification of marine heatwaves') are simply due to this methodological approach.

Please re-formulate.

Response: We thank the reviewer for pointing this out. We agree that the original wording was not sufficiently accurate.

In Huang et al.[1], the authors state that "the increasing trends of the annual intensity, frequency, duration, and areal coverage in the Arctic are closely associated with the increasing surface air temperature and decreasing sea-ice concentration under the global warming environment." We agree with the reviewer that, according to their Table S3, MHW intensity (SSTA) is not significantly correlated with sea ice concentration, although several other MHW characteristics, including frequency and duration, show significant correlations.

Regarding the reviewer's concern about the methodology in He et al.[2], we note that areas with SIC >35% are relatively extensive during 1985–2014 but become much less extensive by 2071–2100. Therefore, we acknowledge that the methodological treatment of sea ice-covered regions should be considered when interpreting the reported "Arctic Amplification of marine heatwaves".

Therefore we decide not to use He et al.[2] and now only cite Huang et al.[1] to support this statement. The sentence has been revised as follows (Page 3, Lines 57–58): "*In the Arctic, declining sea ice has been associated with changes in MHW characteristics[41].*"

Fig. 3: I was asking for using more intuitive units of sea ice concentration response during MHW months, simply by showing the temporal mean (as in Fig. 1), and not the temporal sum. Your response to this is that in Fig. 3 you want to 'focus on contribution of sea ice reduction to the integrated FCO2 anomaly'. I still think that it would be more helpful to provide the sea ice

reduction averaged, and not summed, over all MHW months, in %. Nobody can comprehend the shown numerical values of temporally summed sea ice concentration anomalies. Everybody, in contrast, can understand sea ice concentration anomalies in %. I think it's important how science is communicated. Also, L204ff, where you refer to Fig. 3, could, very likely, stay as it is. You can leave Fig. 3 it as it is though.

Response: We thank the reviewer for this helpful suggestion. We agree that showing the averaged sea ice fraction anomalies during MHW months provides a more intuitive representation and improves the communication of our results. Following the reviewer's suggestion, we have revised Fig. 3 as follows:

"

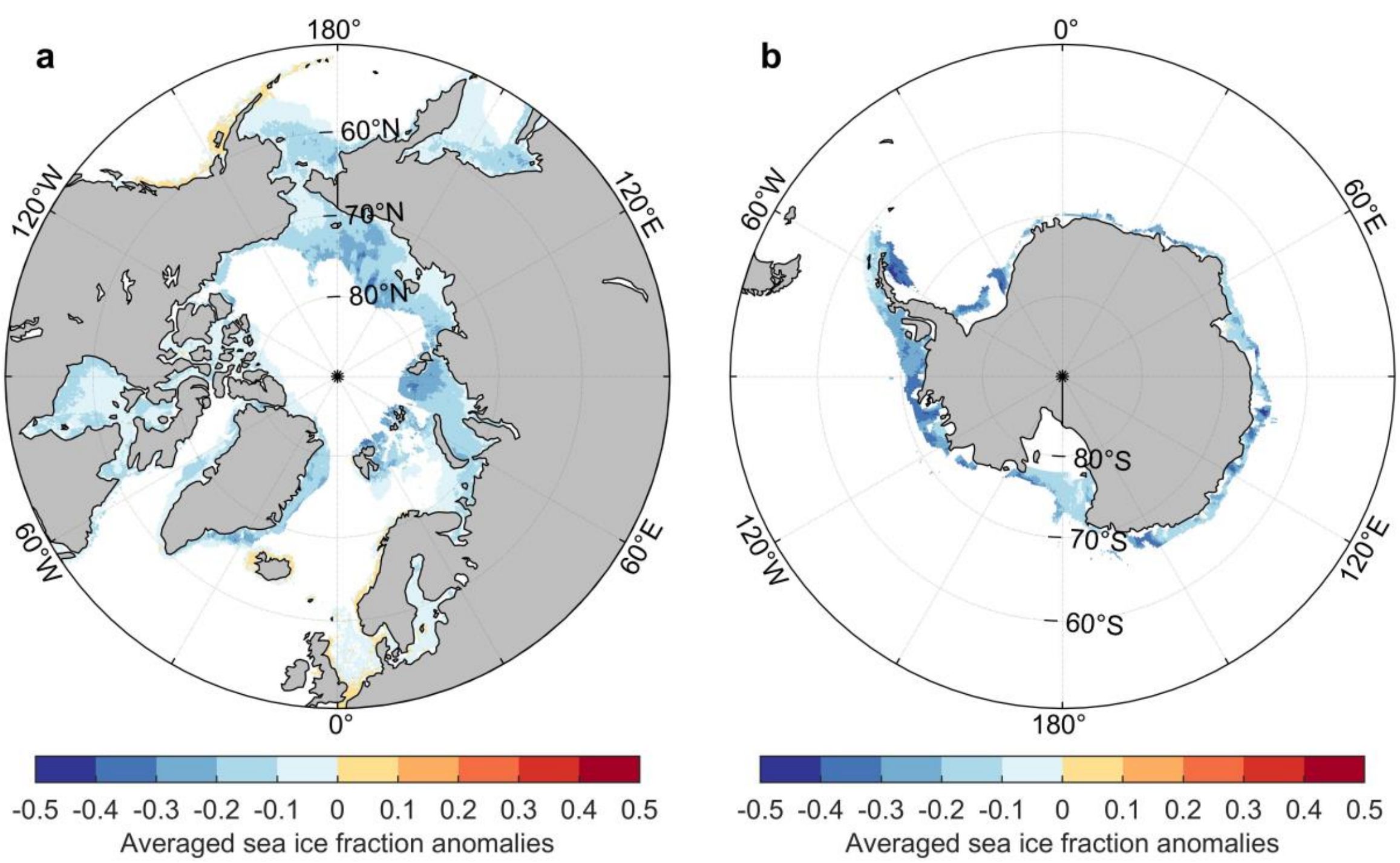

Fig. 3 | Sea ice reductions during MHW months. a*, *b, *Spatial distribution of averaged sea ice fraction anomalies during MHW months (unitless) in the Arctic and subpolar Northern Hemisphere (**a**) and the Antarctic region (**b**). All data shown are derived from the observation-based ULB–SOM–FFN–coastalv2 dataset*[9]."

We have revised the text accordingly (Page 8, Lines 204–206): "*Analysis of averaged sea ice fraction anomalies during MHW months reveals widespread reductions in sea ice cover*".

Fig. 4: Thank you for the explanation. You write that ‘during MHWs the advective export of OC is reduced’. In my view, Fig. 4 shows the contrary: the ‘Δadv exp OC’ bars are positive, i.e. larger.

Why not simply using the same sign convention for both advection terms? Their labels could be simplified to ‘Δadv IC’ and ‘Δadv OC’ and the plot would, in my view, be more consistent with the given ‘gain’ and ‘loss’ labels.

Response: We thank the reviewer for pointing this out. What we intended to convey in our previous response was that reduced advective export of OC leads to positive OC retention.

We agree that simplifying the labels to “Δadv IC” and “Δadv OC” will reduce potential confusion and make the figure more consistent with the gain/loss framework.

We have revised Fig. 4 as follows:

“

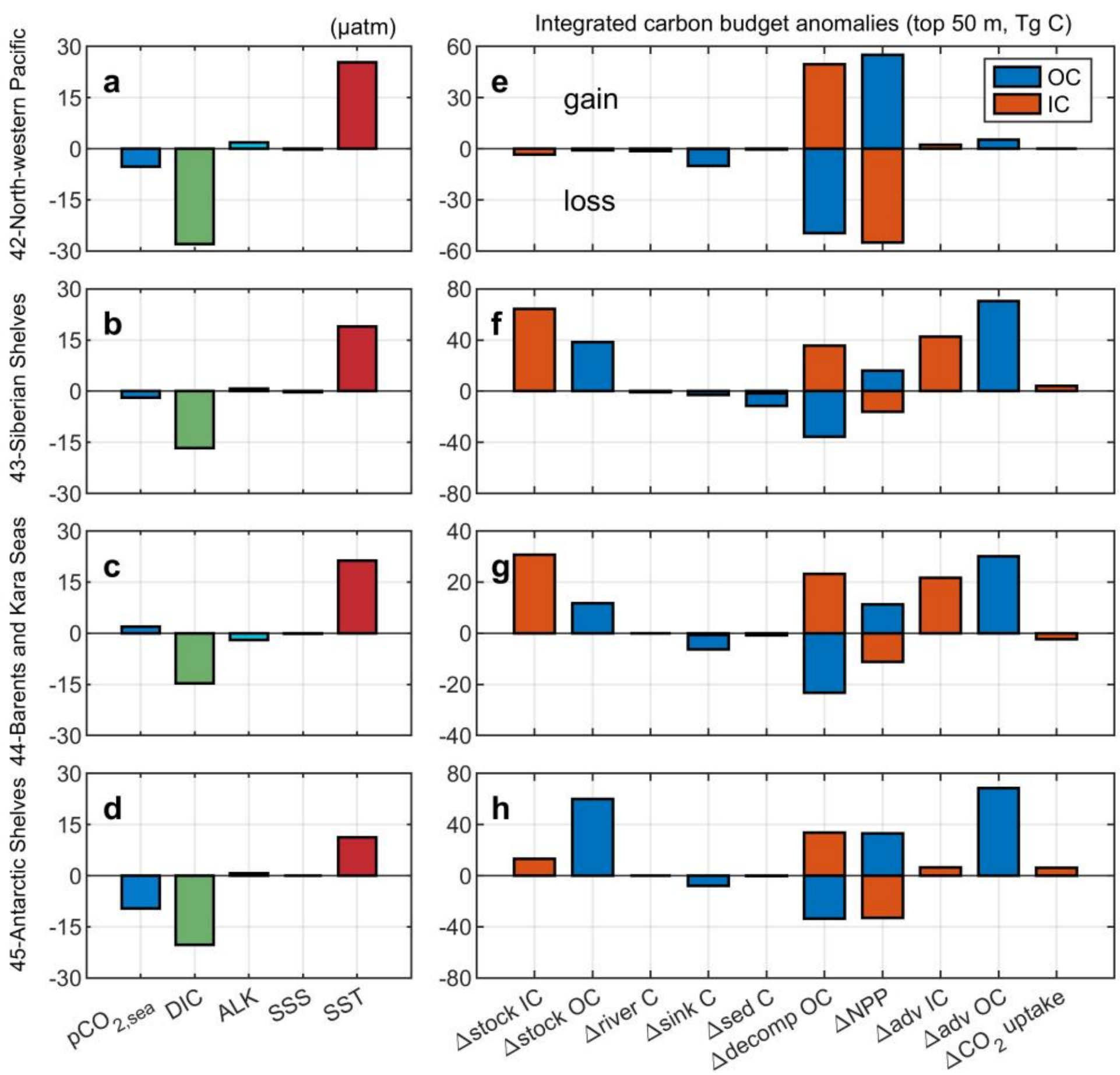

Fig. 4 | Mechanisms driving non-thermal $pCO_{2,sea}$ anomalies and coastal carbon budget during MHW months from 1985 to 2020. a–d*, Relative contributions of DIC, ALK, and SSS to non-thermal $pCO_{2,sea}$ anomalies in four typical coastal regions: (**a**) the North-western Pacific, (**b**) the Siberian Shelves, (**c**) the Barents and Kara Seas, and (**d**) the Antarctic Shelves, derived from the observation-based CMEMS-LSCE dataset[62]. The SST contributions are also shown in the fifth column. **e–h**, Integrated anomalies (Tg C) in the upper-ocean (top 50 m) carbon budget of inorganic carbon (IC) and organic carbon (OC) during MHW months for the same regions, based on the ICON-Coast model[52].*"

L247: 'While simulated … is largely consistent with the observed …'

To me this formulation implies that you compare model results against obervations/data products, which is not the case. Please reformulate.

Response: We agree that the original wording could imply a direct comparison between the model results and the observation, which was not our intention. We have therefore revised the sentence as follows (Page 9, Lines 247–248): "*While simulated surface DIC anomalies broadly exhibit negative anomalies that agree qualitatively with the observed negative DIC anomalies*".

L248: 'changes in the carbon budget' → 'changes in the vertically integrated carbon budget'

Response: Revised as suggested.

L366: Thanks for explicitly saying that you apply detrending prior to extreme event detection to focus on internal climate variability. However, I would have wished that you simply state the effect of the detrending on the estimate of global coastal carbon uptake during MHW months: 11% versus 4.3% (from 1st version of manuscript without detrending). I think it's a valuable piece of information for the community. Please add it.

Response: We thank the reviewer for this helpful suggestion. We agree that explicitly stating the quantitative effect of detrending provides valuable information for the community and helps readers interpret our results.

We have therefore added the following text to the Methods section (Page 14, Lines 378–381): "*Notably, applying detrending prior to MHW detection increased the estimated enhancement of global coastal CO_2 uptake during MHW months from 4.3% to 11.0%. This reflects the exclusion of relatively weak events associated with smaller FCO_2 anomalies and highlights the sensitivity of the estimated response to the choice of MHW detection approach.*"

L458: Thanks for the provided explanation. I still don't understand why you follow this complicated workflow A of Mignot et al., 2022 and Müller et al., 2025:

trend(t_i) = calc_linear_trend(data(t_i))	A1
detrended_data(t_i) = data(t_i) – trend(t_i)	A2
monthly_clim_of_detrended_data(mon_j) = calc_monthly_clim(detrended_data(t_i))	A3
monthly_clim(mon_j) = trend(t_i) + monthly_clim_of_detrended_data(mon_j)	A4
monthly_anomaly(mhw_mon_k) = data(mhw_mon_k) - monthly_clim(mon_j)	A5

, instead of the much simpler workflow B:

monthly_clim(mon_j) = calc_monthly_clim(data(t_i))	B1
monthly_anomaly(mhw_mon_k) = data(mhw_mon_k) - monthly_clim(mon_j)	B2

As far as I understand, both climatologies A4 and B1 are 'trend-preserving'. How do they, and thus A5 and B2, differ? I am not expecting that you apply workflow B, you can leave it as it is.

Also, now it's not clear to me how you applied step A4: Which t_i of trend(t_i) were used for mon_j? Thanks for clarification.

Response: We thank the reviewer for this question. To illustrate the difference between the two approaches, we provide an example of an FCO_2 time series at 132.125°W, 51.375°N derived from the ULB–SOM–FFN–coastalv2 dataset, as shown below. The trend-preserving climatology obtained from workflow A (A4) is shown as the blue line in panel a. In contrast, the traditional climatology obtained from workflow B (B1) is shown as the blue line in panel b. This approach represents a traditional climatology calculated directly from the original time series and does not preserve the time-varying background trend.

In step A4, monthly_clim_of_detrended_data(mon_j) is repeated for each corresponding month throughout the time series to obtain monthly_clim_of_detrended_data(**t_i**) (green line). The linear trend trend(t_i) is then added back at each time step to reconstruct the trend-preserving climatology (blue line).

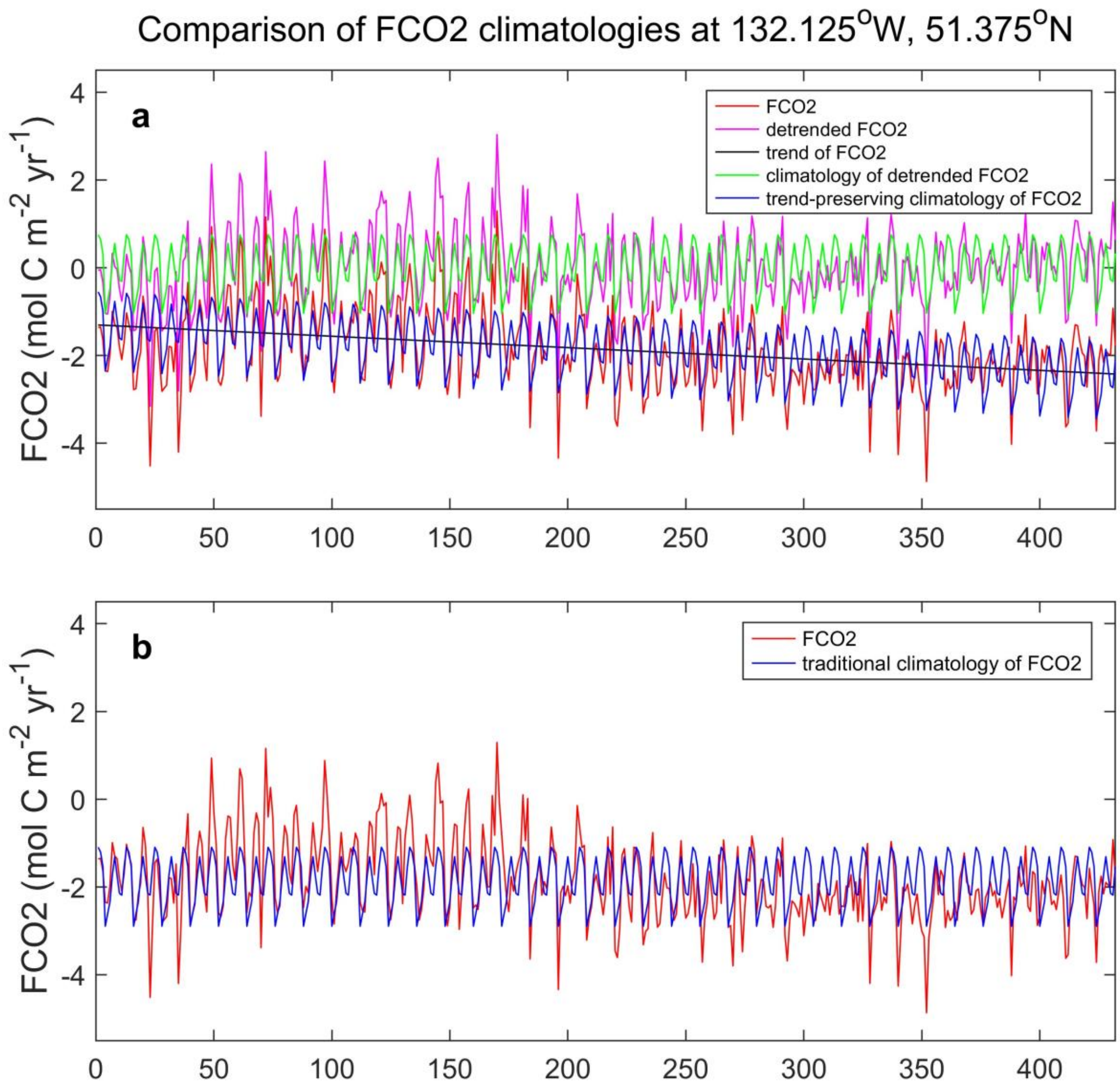

L474: Yes, your overbar denotes the (trend-preserving) monthly climatology. Using the notation above, the zero-order term from your eq. 3 is

$$\bar{a}\bar{b}\bar{c}: \qquad (1)$$

monthly_clim_a(mon_j) = calc_monthly_clim(data_a(t_i))<br>monthly_clim_b(mon_j) = calc_monthly_clim(data_b(t_i))<br>monthly_clim_c(mon_j) = calc_monthly_clim(data_c(t_i))<br>monthly_clim_a_times_b_times_c(mon_j) = monthly_clim_a(mon_j) *<br>monthly_clim_b(mon_j) * monthly_clim_c(mon_j)	
$\overline{\bar{a}\bar{b}\bar{c}}$:<br>monthly_clim_of_monthly_clim_a_times_b_times_c(mon_j) =<br>calc_monthly_clim(monthly_clim_a_times_b_times_c(mon_j) =<br>monthly_clim_a_times_b_times_c(mon_j)	(2)
$\bar{a}\bar{b}\bar{c} - \overline{\bar{a}\bar{b}\bar{c}}$:<br>monthly_clim_a_times_b_times_c(mon_j) –<br>monthly_clim_of_monthly_clim_a_times_b_times_c(mon_j) = 0	(3)
$\bar{a}\bar{b}\bar{c} - \overline{abc}$:<br>data_abc(t_i) = data_a(t_i) * data_b(t_i) * data_c(t_i)<br>monthly_clim_abc(mon_j) = calc_monthly_clim(data_abc(t_i))<br>monthly_clim_a_times_b_times_c(mon_j) – monthly_clim_abc(mon_j) ≠ 0	(4)

I think we agree on (1). But as far as I understand, (2) does not make sense since the monthly climatology of a monthly climatology is the monthly climatology itself, unchanged. Hence, (3) is zero. Note that (4), in contrast, is not zero. Did you calculate (4) instead of (3)? If yes, the correct notation would be $\bar{a}\bar{b}\bar{c} - \overline{abc}$.

Please provide a plot that shows the zero order term $\bar{a}\bar{b}\bar{c} - \overline{\bar{a}\bar{b}\bar{c}}$, i.e. (3), being different from numerical precision.

Response: We thank the reviewer for this question. For (1), $\overline{abc} \neq \bar{a}\bar{b}\bar{c}$, because the climatology of the product is not equivalent to the product of the individual climatologies.

Based on the figure we show in the response in L458, the trend-preserving climatology is reconstructed as the sum of the linear trend and the climatology calculated from detrended $F\mathrm{CO_2}$. Therefore, the monthly climatology of a monthly climatology is indeed the monthly climatology itself, unchanged.

To further illustrate the difference between $\bar{a}\bar{b}\bar{c}$ and $\overline{\bar{a}\bar{b}\bar{c}}$, We provide an example of the Reynolds decomposition of $F\mathrm{CO_2}$ anomalies at 109.375°W, 71.125°S derived from the ULB–SOM–FFN–coastalv2 dataset. For the zero-order term, $\bar{a}\bar{b}\bar{c} - \overline{\bar{a}\bar{b}\bar{c}}$, panel d shows that $\bar{a}\bar{b}\bar{c} \neq \overline{\bar{a}\bar{b}\bar{c}}$.

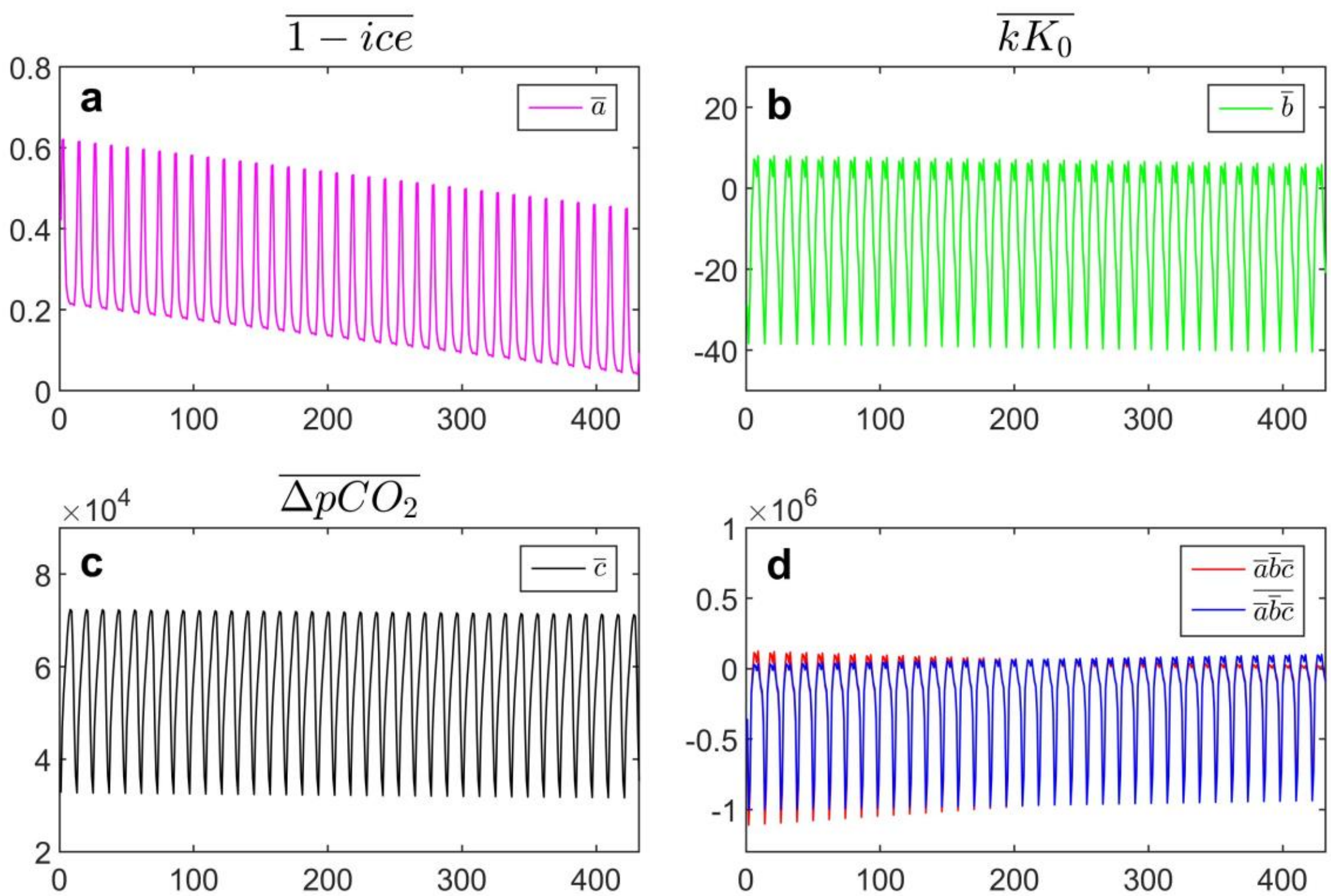

References

He et al., 2024: https://www.nature.com/articles/s41467-024-52760-1

Huang et al., 2021: https://agupubs.onlinelibrary.wiley.com/doi/10.1029/2021GL095590

Mignot et al., 2022: https://www.nature.com/articles/s41467-022-31983-0

Muller et al., 2025: https://www.nature.com/articles/s41558-025-02380-4

References for Responses to Reviewer #3

1. Huang, B. et al. Prolonged marine heatwaves in the Arctic: 1982−2020. *Geophys. Res. Lett.* **48**, e2021GL095590 (2021).
2. He, Y. et al. Arctic Amplification of marine heatwaves under global warming. *Nat. Commun.* **15**, 8265 (2024).

Response to the reviewer's comments (fourth round)

We sincerely thank the reviewer for the positive evaluation and helpful suggestions. We have implemented all of the suggested revisions. Detailed point-by-point responses to each comment (shown in black) are provided below. Our responses are presented in blue, and the corresponding revisions in the manuscript are highlighted in *italic* and underlined text.

Reviewer #3 (Remarks to the Author):

Review 4 of Nature Communications ID NCOMMS-25-92847-T: 'Polar and subpolar shelf seas dominate enhanced coastal CO_2 uptake during marine heatwaves' by Zhang et al.

Table of Contents

Summary..1

Summary

Thank you Zhang et al. for working on all comments of the 3rd review. I am happy to see this published.

Response: We sincerely thank the reviewer for the positive evaluation and encouraging comments.

Please add '… and hence differ between years.' or such to the end of sentence L488.

Response: We thank the reviewer for the suggestion. We have added "*and hence differ between years*" (Page 18, Line 490) as suggested.

Dear Editor, I believe it would be helpful to add the two figures from the authors' response to the third review round (those addressing my comments on lines 458 and 474) to the Supplementary Figures, and to reference them in the corresponding Methods sections. Thank you for consideration.

Response: We thank the reviewer for this helpful suggestion. We have added the two explanatory figures to the Supplementary Information (Supplementary Figs. 11 and 12) and referenced them in the corresponding Methods sections to facilitate understanding of the trend-preserving climatology calculation and the Reynolds decomposition.

The relevant text now reads: (Page 17, Line 464–465) "*For each variable and grid cell, a trend-preserving climatology was calculated over the 1985–2020 period*[47,53] *(see Supplementary Fig. 11 for an example).*" (Page 18, Line 503–504) "*Here, the zero-order term represents baseline contributions from mean states of the drivers (see Supplementary Fig. 12 for an example).*"

The corresponding Supplementary Figure legends are as follows:

(Page 12, Line 102–109 in Supplementary Information) "***Supplementary Fig. 11 | Comparison of trend-preserving and traditional FCO_2 climatologies. a***, *Example of the trend-preserving climatology calculation for the FCO_2 time series at 132.125°W, 51.375°N derived from the ULB–SOM–FFN–coastalv2 dataset. The original FCO_2 time series (red) is first detrended (magenta), and the linear trend (black) is subsequently added back to the climatology of the detrended time series (green) to reconstruct the trend-preserving climatology (blue).* ***b***, *Traditional climatology calculated directly from the original FCO_2 time series without detrending. This example illustrates the difference between the trend-preserving and traditional climatology approaches.*"

(Page 13, Line 111–116 in Supplementary Information) "***Supplementary Fig. 12 | Example illustrating the zero-order term in the Reynolds decomposition.*** *Example of the Reynolds decomposition for FCO_2 at 109.375°W, 71.125°S derived from the ULB–SOM–FFN–coastalv2 dataset.* ***a–c***, *Trend-preserving climatological states of $1 - ice$ ($\bar{a}$), kK_0 ($\bar{b}$), ΔpCO_2 ($\bar{c}$), respectively.* ***d***, *Comparison between the product of the individual climatological states ($\bar{a}\bar{b}\bar{c}$) and the climatology of their product ($\overline{\bar{a}\bar{b}\bar{c}}$), illustrating that $\bar{a}\bar{b}\bar{c} \neq \overline{\bar{a}\bar{b}\bar{c}}$.*"

Kind regards

Chris
